# Supplementary material for: Epigenome and interactome profiling uncovers principles of distal regulation in the barley genome
Source: Cell Genom. 2025 Oct 10;6(1):101037. doi: 10.1016/j.xgen.2025.101037 (PMC12926201; doi:10.1016/j.xgen.2025.101037)
Supplement: Document S1. Figures S1–S11 and Tables S1 and S4–S6 [file mmc1.pdf]

Cell Genomics, Volume *6*

Supplemental information

Epigenome and interactome profiling uncovers principles of distal regulation in the barley genome

Pavla Navratilova, Simon Pavlu, Zihao Zhu, Zuzana Tulpova, Ondrej Kopecky, Petr Novak, Nils Stein, and Hana Simkova

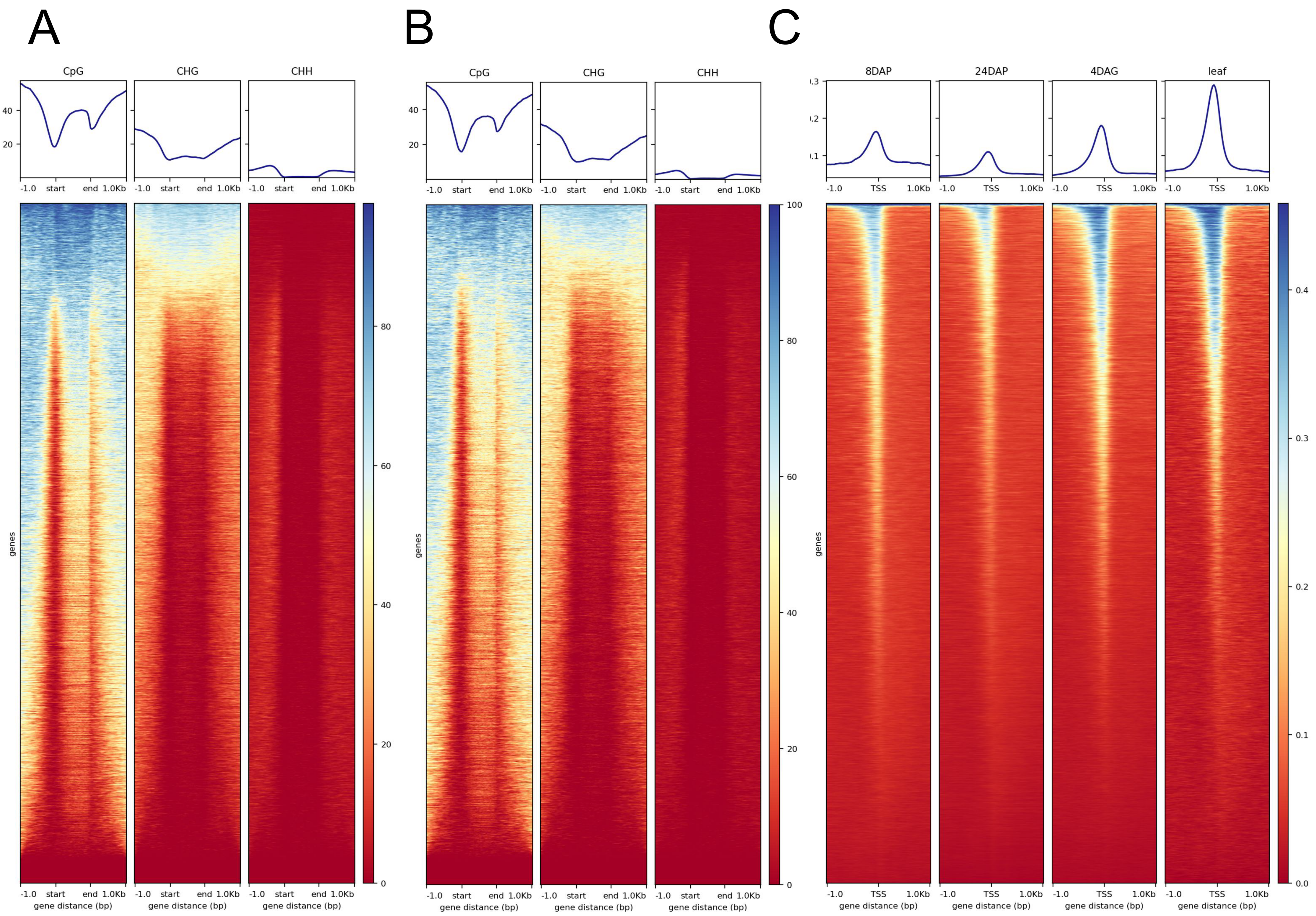

Figure S1. Profiles and heatmaps showing read coverages for two epigenetic features across MorexV3 high-confidence genes (related to Figure 1B). Data from bisulfite sequencing show cytosine methylation in three sequence contexts in (A) 24DAP embryo and (B) leaf. (C) Profiles of ATAC-seq coverages around transcription start sites (TSSs) in the four developmental stages used in the study.

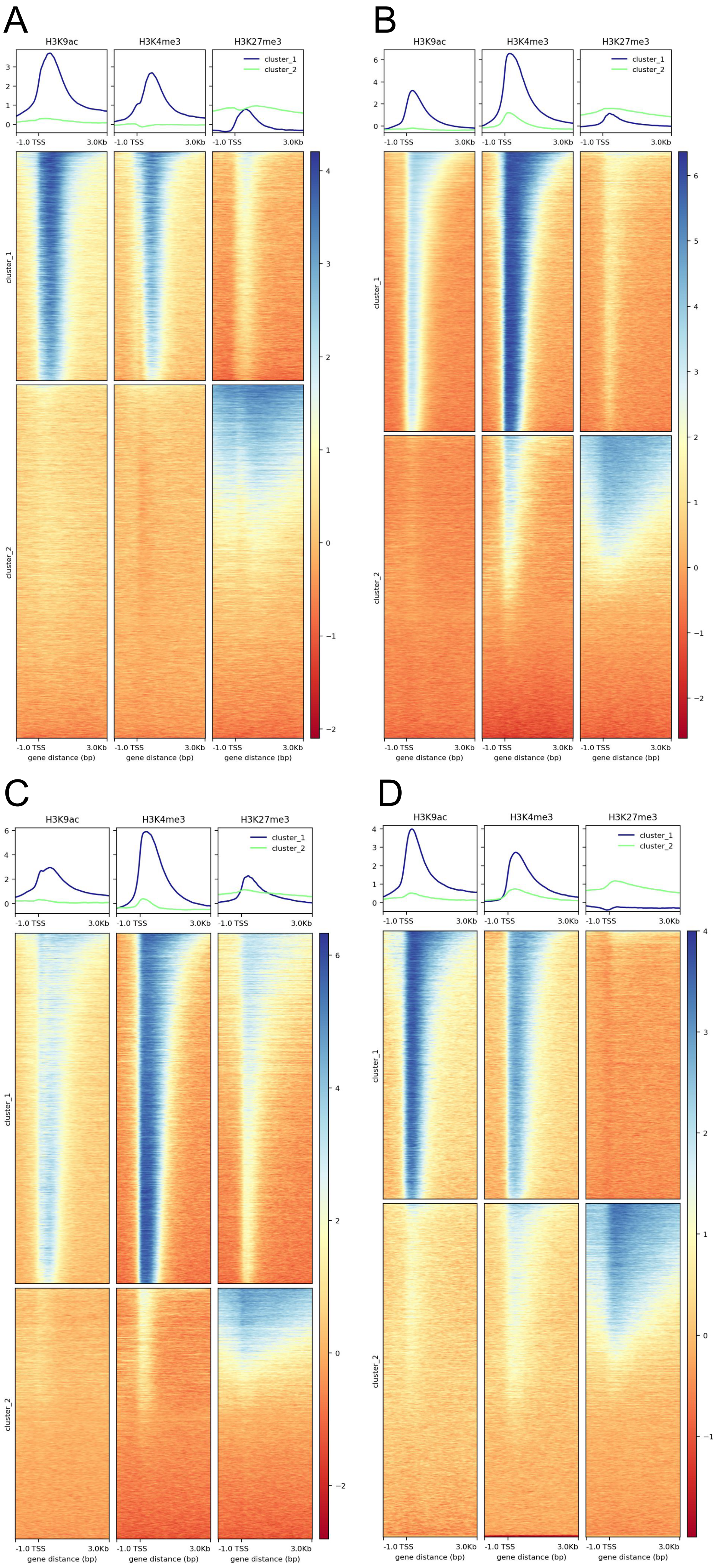

Figure S2. K-means clustered profiles of histone modifications around HC-gene TSSs from ChIP-seq (related to Figure 1B). (A) Profiles in 8DAP embryo. Cluster 1 (top) represents active genes while cluster 2 (bottom) shows profiles of silent genes. (B, C, D) Data from 24DAP (B), 4DAG (C) and leaf (D) samples. The k-means clustering separates two sub-profiles reflecting transcriptional activity.

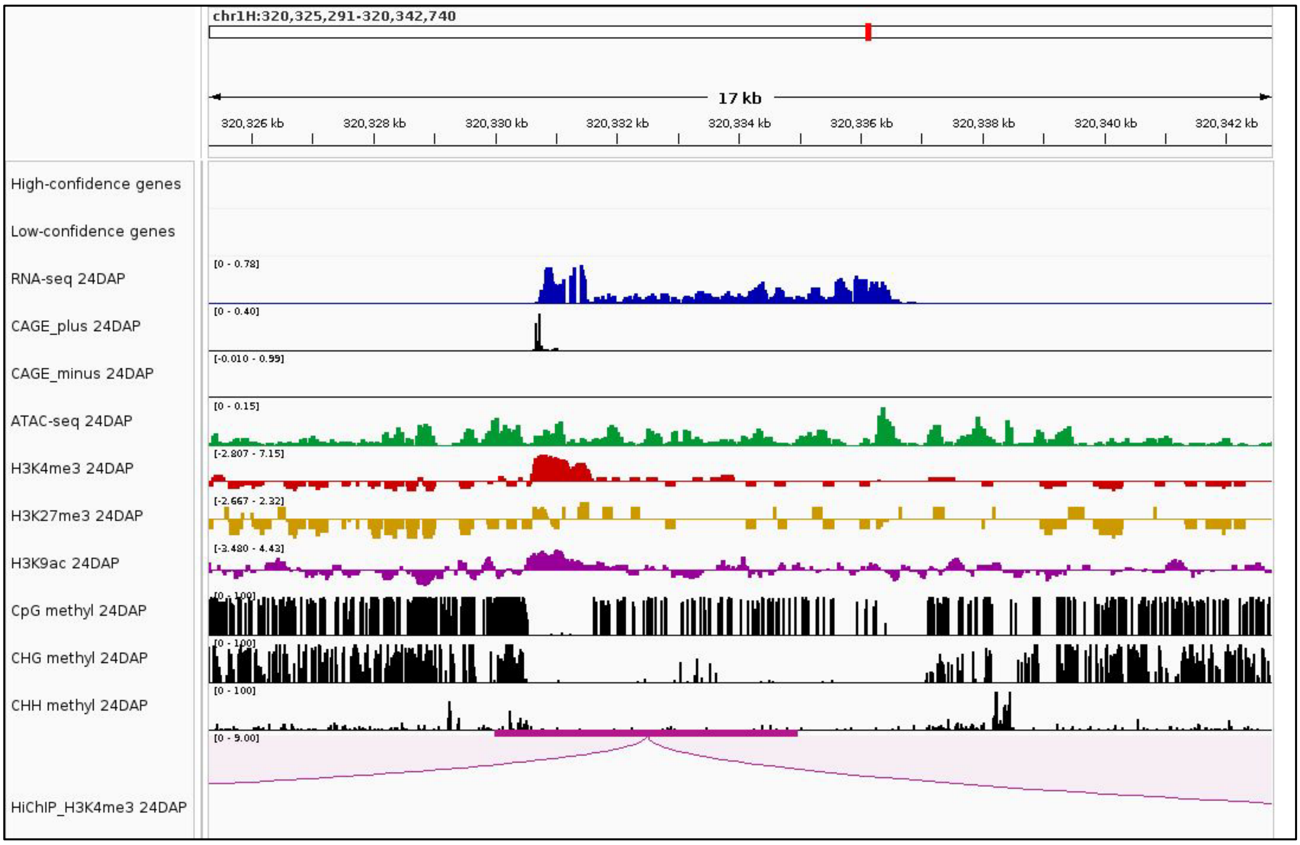

Figure S3. An example of a putative unannotated gene (related to Figure 1C). The occurrence of transcripts and features of active chromatin indicates the presence of an unannotated gene. To prevent misinterpretation of its promoter as an intergenic CRE, we excluded this and similar regions from the analysis of the non-coding genome.

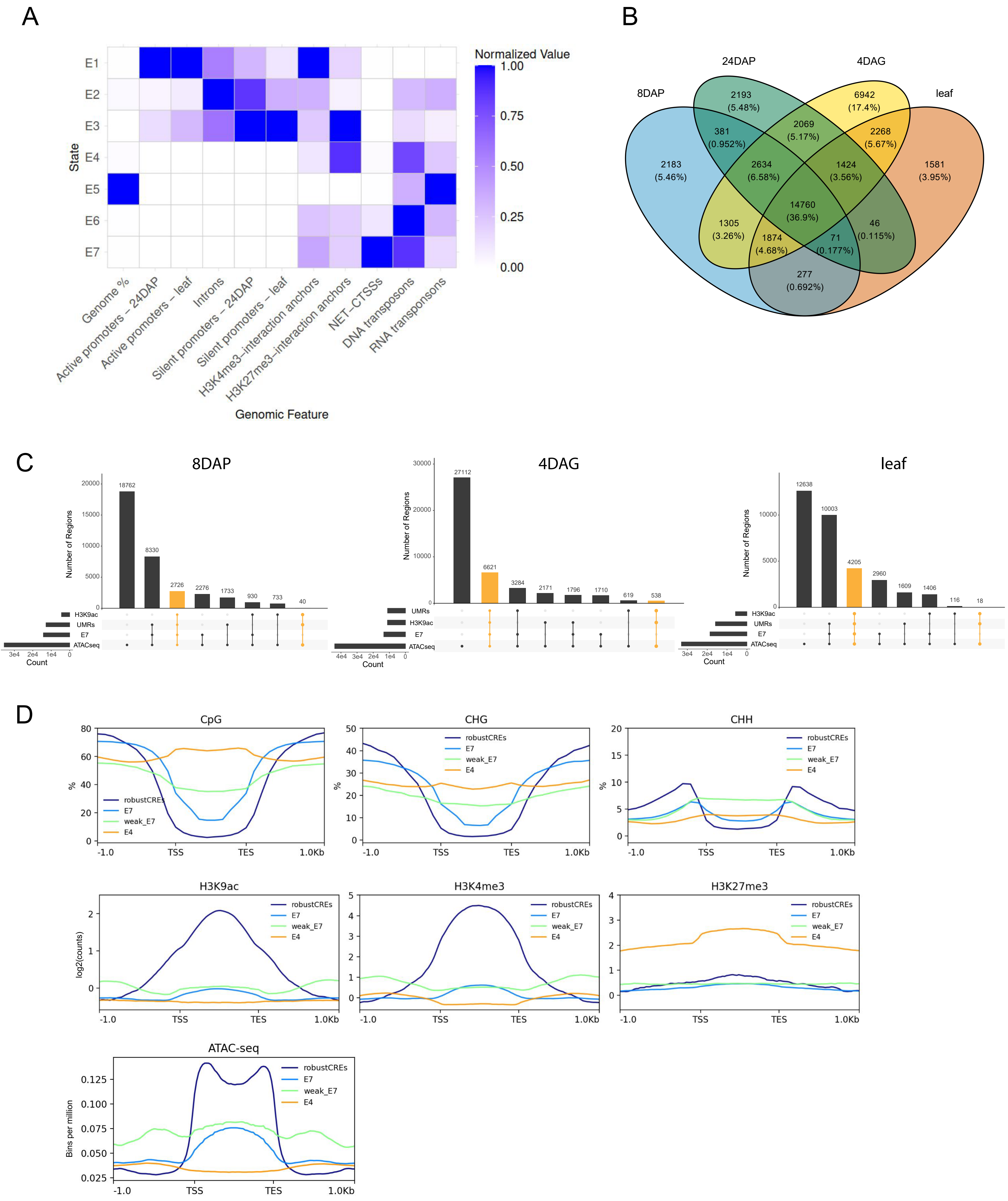

Figure S4. Chromatin state analysis is a sensitive method of cCREs detection (related to Figures 1C, D, and 2A, B). (A) An overlap enrichment of genomic features across 7 chromatin states in the leaf sample showing state dynamics following transcription dynamics between individual stages. (B) Dynamics of chromatin state-E4 segments, potentially comprising silenced cCREs. (C) The overlap of activating intergenic genomic features - peaks of ATAC-seq and H3K9ac and unmethylated regions (UMRs) - defines tissue-specific sets of robust cCREs (highlighted yellow), which shows a high overlap with E7 segments. 'Count' indicates the total number of peaks for ATAC-seq while for E7, H3K9ac and UMRs, overlaps of these datasets with ATAC-seq peaks are counted. (D) Comparison of main chromatin feature profiles across robust cCREs, states E4, E7 and the E7-state segments not overlapped with any of the epigenetic feature peaks (weak E7).

A

8DAP robust CRE motif	e-value	Transcription factor family
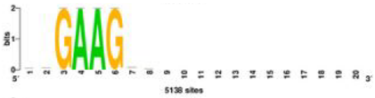	6.5e-20	BBR-BPC
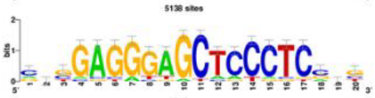		Palindrome of the GAGA motif
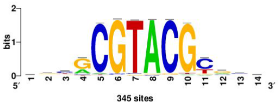	5.1e-07	SPL
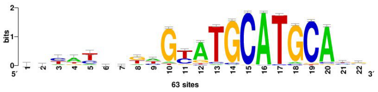	3.6e-06	B3 domain-containing

B

4DAG robust CRE motif	e-value	Transcription factor family
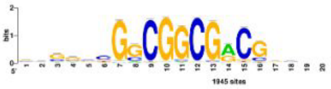	7.6e-101	ERF
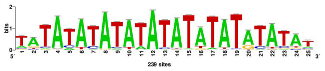	1.9e-63	ZFHD10-3
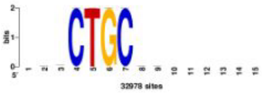	9.8e-07	NAC, ONAC
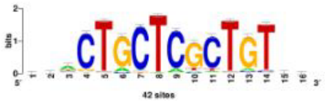	0	No hit

C

leaf robust CRE motif	e-value	Transcription factor family
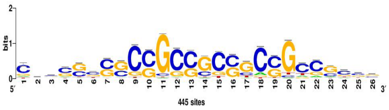	1e-300	ERF
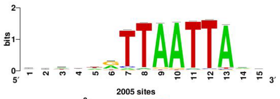	1.5e-32	WOX
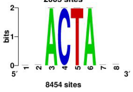	8.9e-04	AP2/ERF and B3 domain-containing

Figure S5. Transcription factor binding sites in robust cCREs (related to Figure 2D). Transcription factor binding sites enriched in (A) 8DAP, (B) 4DAG, and (C) leaf robust cCRE datasets.

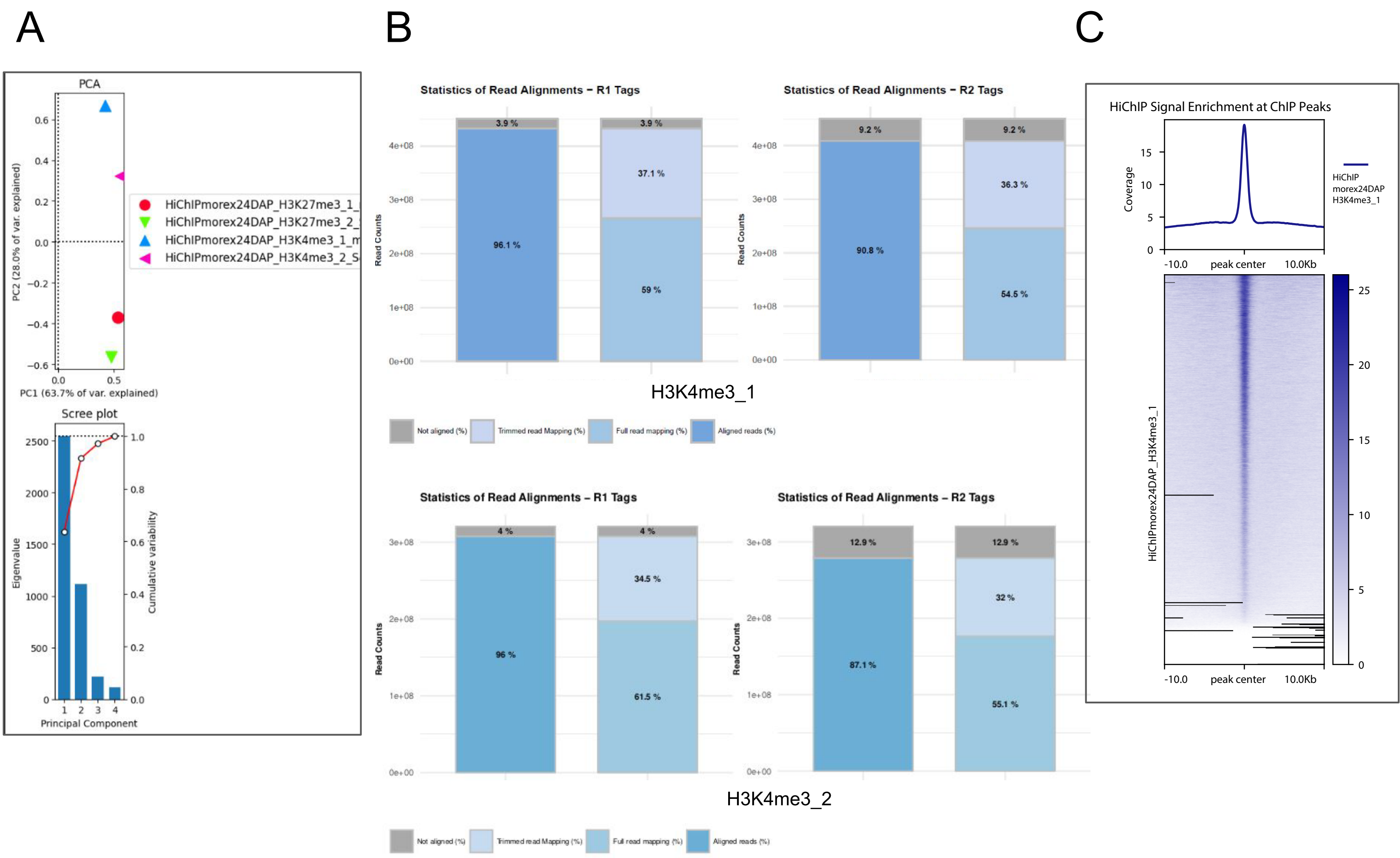

Figure S6. Quality Control of the 24DAP H3K4me3 HiChIP sequencing datasets (related to STAR Methods - HiChIP data analysis). (A) HiChIP replicate correlation of mapped data by Principal Component Analysis (PCA). (B) Mapping statistics of the two 24DAP H3K4me3 HiChIP replicates. (C) HiChIP signal enrichment at the H3K4me3 ChIP peaks as called from previously performed native ChIP-seq experiment.

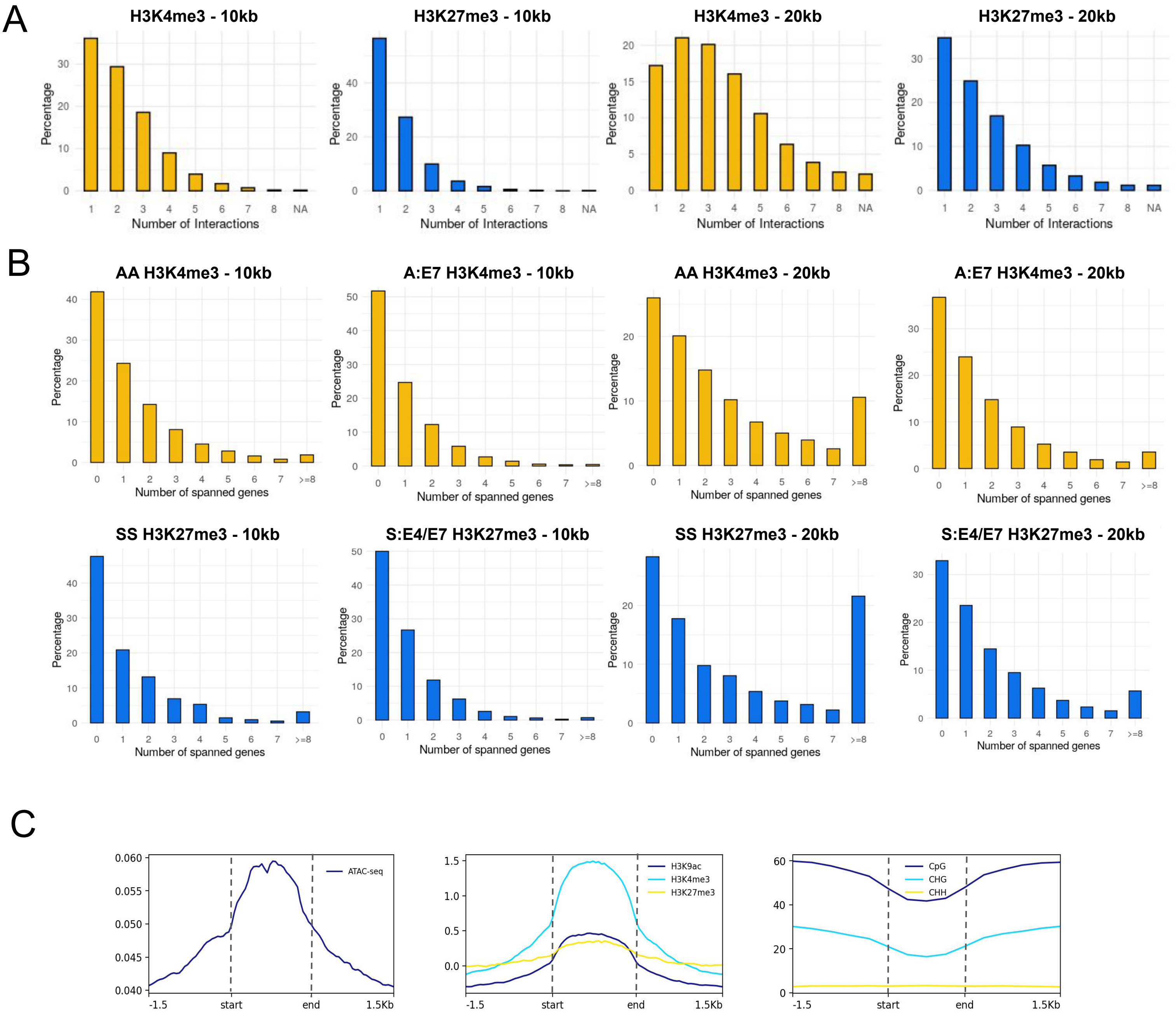

Figure S7. Analysis of the interactome in the 24DAP barley embryo (related to Figure 4). (A) Numbers of H3K4me3 and H3K27me3 HiChIP interactions per promoter at 10- and 20-kb resolution. (B) Numbers of genes spanned by H3K4me3 and H3K27me3 HiChIP interactions at 10- and 20-kb resolution. (C) Enrichments of selected epigenetic features across H3K4me3 HiChIP anchors at 5-kb resolution indicates that they are transcriptionally dense regions.

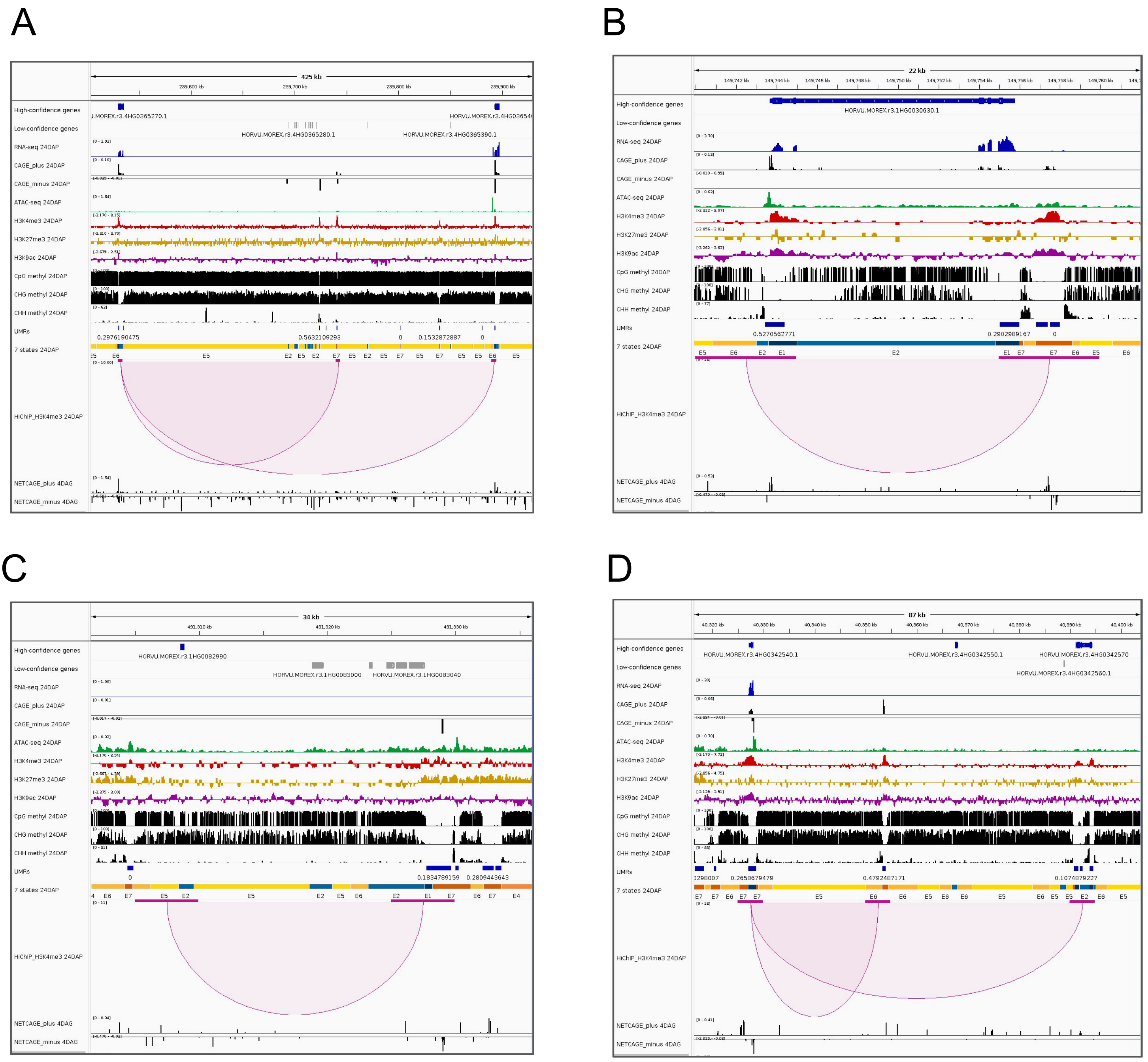

Figure S8. Examples of distinct interaction classes together with epigenomic features (related to Figure 4). (A) Active promoter-active promoter, (B) active self-gene loop, (C) silent promoter-CREc, (D) active promoter-CREc and active promoter-silent gene.

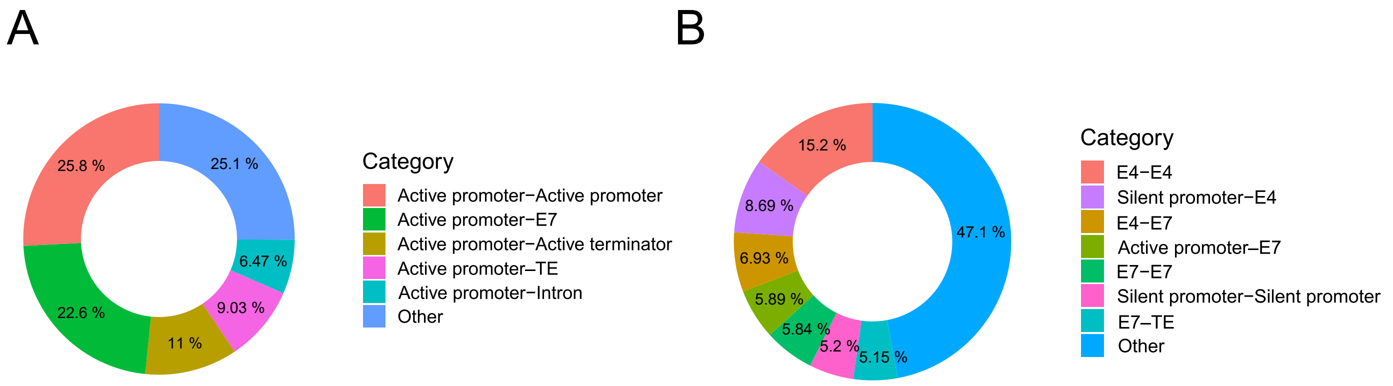

Figure S9. Proportions of interaction classes identified by HiChIP at 5-kb resolution in 24DAP embryo, focusing on high-confidence genes (related to Figure 5). Annotation of all significant interactions associated with (A) activating (H3K4me3) and (B) repressive (H3K27me3) histone mark. The category „Other“ includes all interaction classes with <5% frequency.

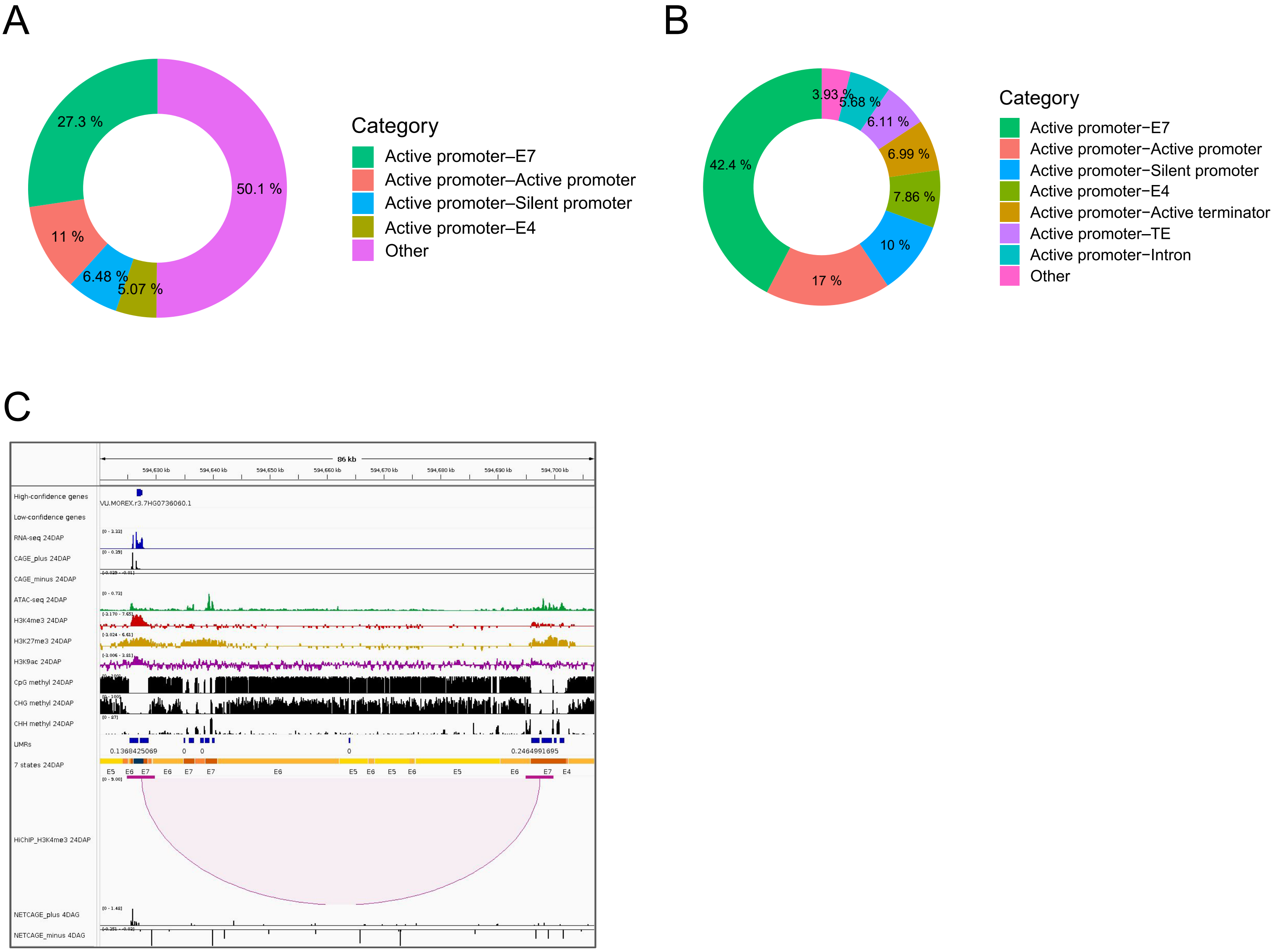

Figure S10. Features of bivalent interactions (related to Figure 5). Annotation of all bivalent interactions (A), and the same set from the active promoter-centric view (B). The category „Other“ includes all interaction classes with <5% frequency. (C) An example of a bivalent interaction.

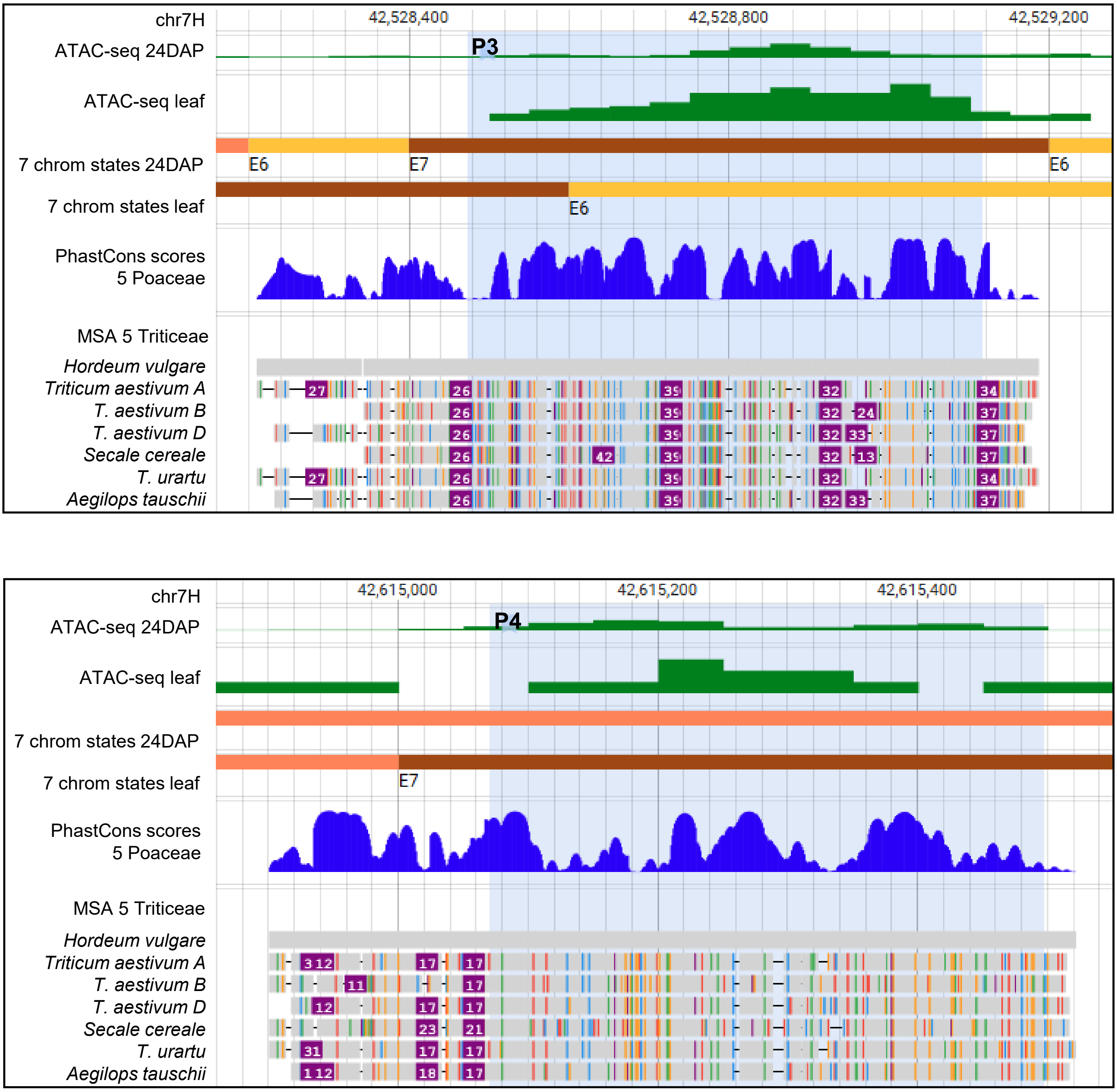

Figure S11. Regulome of the barley Vernalization 3 gene: evolutionary conservation (related to Figure 7). Regions of cCRE3 (top) and cCRE4 (bottom) with BLAST hits of bread wheat enhancers P3 and P4 (highlighted blue) show high sequence conservation (PhastCons scores, scale 0-1) among five *Poaceae* species (*H. vulgare*, *Triticum urartu*, *Aegilops tauschii*, *Secale cereale* and *Brachypodium distachyon*). Multiple sequence alignment (MSA) shows the conservation at single-base resolution.

Table S1. Peak numbers in barley embryo and leaf ChIP-seq and ATAC-seq datasets (related to STAR Methods - ATAC-seq and ChIP-seq data analysis)

ATAC-seq	8DAP
53303	Morex8DAP_ATACseq_1
55758	Morex8DAP_ATACseq_2
42899	Morex8DAP_ATACseq_intersect
66139	Morex8DAP_ATACseq_merged
	**24DAP**
145943	Morex24DAP_ATACseq_1
127166	Morex24DAP_ATACseq_2
93441	Morex24DAP_ATACseq_intersect
	**4DAG**
54098	Morex4DAG_ATACseq_1
29863	Morex4DAG_ATACseq_2
10627	Morex4DAG_ATACseq_intersect
73333	Morex4DAG_ATACseq_merged
	**Leaf**
64153	MorexLeaf_ATACseq_pooled

ChIP-seq	H3K9ac - 8DAP
22130	Morex8DAP_H3K9ac_1
26393	Morex8DAP_H3K9ac_2
15911	Morex8DAP_H3K9ac_intersect
32610	Morex8DAP_H3K9ac_merged
	**24DAP**
32119	Morex24DAG_H3K9ac_1
66104	Morex24DAG_H3K9ac_2
30159	Morex24DAG_H3K9ac_intersect
	**4DAG**
61885	Morex4DAG_H3K9ac_1
30178	Morex4DAG_H3K9ac_2
25994	Morex4DAG_H3K9ac_intersect
46140	Morex4DAG_H3K9ac_merged
	**Leaf**
35765	MorexLeaf_H3K9ac_intersect

ChIP-seq	H3K4me3 - 8DAP
12976	Morex8DAP_H3K4me3_1a
16198	Morex8DAP_H3K4me3_2
10075	Morex8DAP_H3K4me3_intersect
19099	Morex8DAP_H3K4me3_merged
	**24DAP**
47454	Morex24DAP_H3K4me3_1
51563	Morex24DAP_H3K4me3_2
46220	Morex24DAP_H3K4me3_intersect
	**4DAG**
53653	Morex4DAG_H3K4me3_1a
48396	Morex4DAG_H3K4me3_2a
46140	Morex4DAG_H3K4me3_intersect
	**Leaf**
19951	MorexLeaf_H3K4me3_pooled

ChIP-seq	H3K27me3 - 8DAP
46915	Morex8DAP_H3K27me3_1
43595	Morex8DAP_H3K27me3_2
38506	Morex8DAP_H3K27me3_intersect
	**24DAP**
59006	Morex24DAP_H3K27me3_1
51373	Morex24DAP_H3K27me3_2
48316	Morex24DAP_H3K27me3_intersect
	**4DAG**
71668	Morex4DAG_H3K27me3_1a
85421	Morex4DAG_H3K27me3_2a
70564	Morex4DAG_H3K27me3_intersect
	**Leaf**
37098	MorexLeaf_H3K9ac_intersect

Intergenic peaks Filtered using "Coding potential set of 77364 regions including HC and LC genes, lncRNA candidates and RNA-seq positive regions"

ATAC-seq	8DAP
35530	Intergenic_8DAP_ATACseq_intersect
	**24DAP**
55250	Intergenic_24DAP_ATACseq_intersect
	**4DAG**
43851	Intergenic_4DAG_ATACseq_merged
	**Leaf**
32955	Intergenic_Leaf_pooledReps

H3K9ac	8DAP
	8009
	**24DAP**
	14351
	**4DAG**
	27579
	**Leaf**
	8485

H3K4me3	8DAP
	4527
	**24DAP**
	11810
	**4DAG**
	11764
	**Leaf**
	1864

UMRs	
total	139283
intergenic	74614

Table S4. NET-CAGE statistics and quality control (related to STAR Methods - NET-CAGE data analysis)

Sample	Total	rRNA-filtered	BWA-Mapped		BWA-Others		Totally Mapped	Totally Used
				Total	HISAT2_mapped	HISAT2_Unique		
N.4DAG.1	49,565,707	49,565,707 (100.00%)	17,044,862 (34.39%)	35,287,860 (71.19%)	28,886,338 (81.86%)	4,349,102 (15.06%)	45,931,200 (92.67%)	21,393,964 (46.58%)
N.4DAG.2	42,976,826	42,976,826 (100.00%)	17,103,499 (39.80%)	31,220,523 (72.65%)	20,964,092 (67.15%)	3,143,385 (14.99%)	38,067,591 (88.58%)	20,246,884 (53.19%)

rRNA-filtered: the retained reads after the reads from rRNA were filtered.
BWA-Mapped: BWA-mapped read with MAPQ >= 20.
BWA_Others: the read with MAPQ < 20 in BWA mapping. These reads were mapped again by HISAT2.
HISAT2_Unique: HISAT2-mapped with the tag NH:i:1
Totally Mapped: sum of BWA-Mapped and HISAT2-mapped.
Totally Used: sum of BWA-Mapped and HISAT2_Unique. These reads were used for subsequent analysis.

Table S5. Numbers of interactions in all HiChIP samples as identified by FitHiChIP (related to STAR Methods - HiChIP data analysis)

Embryo stage	Histone modification	Resolution	FDR value	Interactions Counts	Single-feature Counts
24DAP	H3K27me3	20kb	0,05	46525	18588
24DAP	H3K27me3	20kb	0,1	47291	17811
24DAP	H3K27me3	10kb	0,1	19323	NA
24DAP	H3K27me3	5kb	0,05	1193	657
24DAP	H3K27me3	5kb	0,1	3751	2158
24DAP	H3K4me3	20kb	0,05	46213	13893
24DAP	H3K4me3	20kb	0,1	57476	18288
24DAP	H3K4me3	10kb	0,1	28578	NA
24DAP	H3K4me3	5kb	0,05	8038	4014
24DAP	H3K4me3	5kb	0,1	9991	5034

Table S6. Quantification of various types of interactions in the HiChIP significant-interaction set (related to Figure 5 and Figure S9)

(A) 24DAP_H3K4me3_Q0.1 interaction set at 5 kb resolution

category	count
TE-TE	124
TE-Active_promoter	902
TE-Active_terminator	112
TE-E7	337
TE-E4	22
TE-Intron	62
TE-Silent_promoter	56
TE-Silent_terminator	9
TE-Distal	4
Active_promoter-Active_promoter	2578
Active_promoter-Active_terminator	1104
Active_promoter-E7	2255
Active_promoter-E4	116
Active_promoter-Intron	646
Active_promoter-Silent_promoter	424
Active_promoter-Silent_terminator	72
Active_promoter-Distal	26
Active_terminator-Active_terminator	42
Active_terminator-E7	274
Active_terminator-E4	9
Active_terminator-Intron	47
Active_terminator-Silent_promoter	45
Active_terminator-Silent_terminator	6
Active_terminator-Distal	3
E7-E7	236
E7-E4	47
E7-Intron	135
E7-Silent_promoter	89
E7-Silent_terminator	16
E7-Distal	7
E4-E4	32
E4-Intron	10
E4-Silent_promoter	37
E4-Silent_terminator	4
E4-Distal	0
Intron-Intron	32
Intron-Silent_promoter	25
Intron-Silent_terminator	1
Intron-Distal	1
Silent_promoter-Silent_promoter	36
Silent_promoter-Silent_terminator	7
Silent_promoter-Distal	0
Silent_terminator-Silent_terminator	0
Silent_terminator-Distal	1
Distal-Distal	0

Table S6. Quantification of various types of interactions in the HiChIP significant-interaction set (related to Figure 5 and Figure S9)

(B) 24DAP_H3K27me3_Q0.1 interaction set at 5 kb resolution

category	count
Intron-Intron	45
Intron-Silent_promoter	59
Intron-Silent_terminator	21
Intron-E4	68
Intron-E7	77
Intron-Active_promoter	41
Intron-TE	30
Intron-Active_terminator	17
Intron-Distal	0
Silent_promoter-Silent_promoter	195
Silent_promoter-Silent_terminator	117
Silent_promoter-E4	326
Silent_promoter-E7	170
Silent_promoter-Active_promoter	108
Silent_promoter-TE	100
Silent_promoter-Active_terminator	33
Silent_promoter-Distal	0
Silent_terminator-Silent_terminator	3
Silent_terminator-E4	69
Silent_terminator-E7	28
Silent_terminator-Active_promoter	12
Silent_terminator-TE	13
Silent_terminator-Active_terminator	5
Silent_terminator-Distal	0
E4-E4	571
E4-E7	260
E4-Active_promoter	115
E4-TE	184
E4-Active_terminator	33
E4-Distal	3
E7-E7	219
E7-Active_promoter	221
E7-TE	193
E7-Active_terminator	78
E7-Distal	4
Active_promoter-Active_promoter	68
Active_promoter-TE	75
Active_promoter-Active_terminator	68
Active_promoter-Distal	1
TE-TE	86
TE-Active_terminator	23
TE-Distal	3
Active_terminator-Active_terminator	8
Active_terminator-Distal	1
Distal-Distal	0
