# Supplementary material for: Epigenome and interactome profiling uncovers principles of distal regulation in the barley genome
Source: Cell Genom. 2025 Oct 10;6(1):101037. doi: 10.1016/j.xgen.2025.101037 (PMC12926201; doi:10.1016/j.xgen.2025.101037)
Supplement: Document S2. Article plus supplemental information [file mmc4.pdf]

Cell Genomics

Article

Epigenome and interactome profiling uncovers principles of distal regulation in the barley genome

Graphical abstract

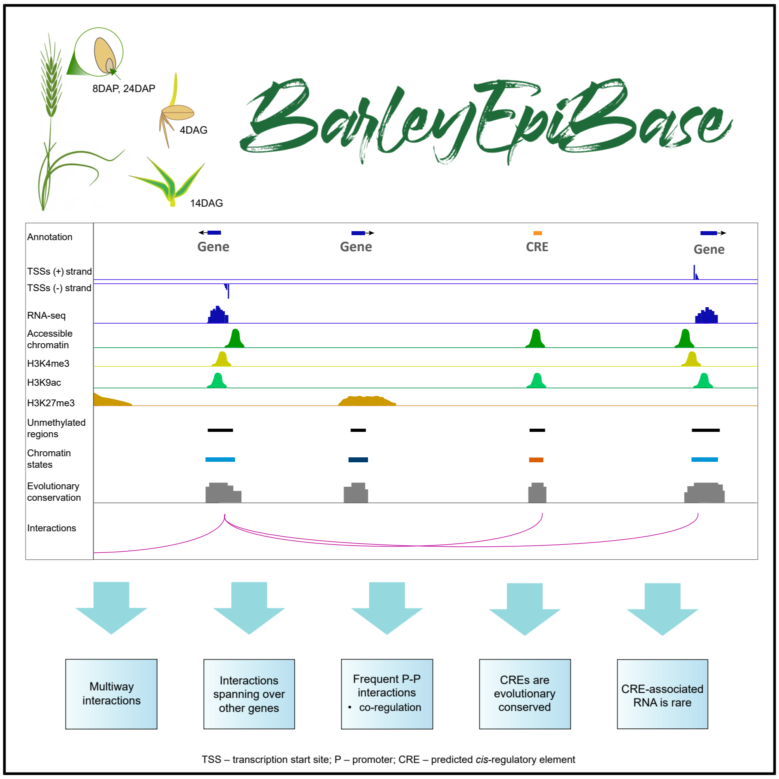

Highlights

- Epigenome profiling predicted regulatory potential for 1.4% of the barley genome
- Distal *cis*-regulatory elements are detectable even for inactive target genes
- Frequent promoter-promoter interactions are associated with gene co-regulation
- The generated data can be accessed via an interactive epigenome browser

Authors

Pavla Navratilova, Simon Pavlu, Zihao Zhu, ..., Petr Novak, Nils Stein, Hana Simkova

Correspondence

pavla.navratilova@ceitec.muni.cz (P.N.), simkovah@ueb.cas.cz (H.S.)

In brief

Navratilova et al. profiled several epigenetic features across three stages of barley embryo and leaves and predicted regulatory potential for 1.4% of the barley genome. Using HiChIP, they linked part of the predicted regulatory elements with their putative targets. The generated datasets can be viewed in an interactive epigenome browser.

Navratilova et al., 2026, Cell Genomics *6*, 101037
January 14, 2026 © 2025 The Authors. Published by Elsevier Inc.
https://doi.org/10.1016/j.xgen.2025.101037

CellPress

Cell Genomics

CellPress
OPEN ACCESS

Article

Epigenome and interactome profiling uncovers principles of distal regulation in the barley genome

Pavla Navratilova,[1,*] Simon Pavlu,[1,2] Zihao Zhu,[3] Zuzana Tulpova,[1] Ondrej Kopecky,[1,2] Petr Novak,[4] Nils Stein,[3,5] and Hana Simkova[1,6,*]
[1]Institute of Experimental Botany of the Czech Academy of Sciences, Olomouc, Czech Republic
[2]Department of Cell Biology and Genetics, Faculty of Science, Palacky University, Olomouc, Czech Republic
[3]Leibniz Institute of Plant Genetics and Crop Plant Research, Gatersleben, Germany
[4]Czech Academy of Sciences, Institute of Plant Molecular Biology, Ceske Budejovice, Czech Republic
[5]Crop Plant Genetics, Martin Luther University of Halle-Wittenberg, Halle (Saale), Germany
[6]Lead contact
*Correspondence: pavla.navratilova@ceitec.muni.cz (P.N.), simkovah@ueb.cas.cz (H.S.)
https://doi.org/10.1016/j.xgen.2025.101037

SUMMARY

Regulation of transcription initiation is the ground level of modulating gene expression during plant development. This process relies on interactions between transcription factors and *cis*-regulatory elements (CREs), which become promising targets for crop bioengineering. To annotate CREs in the barley genome and understand mechanisms of distal regulation, we profiled several epigenetic features across three stages of barley embryo and leaves and performed HiChIP to identify activating and repressive genomic interactions. Using machine learning, we integrated the data into seven chromatin states, predicting ∼77,000 CRE candidates, collectively representing 1.43% of the barley genome. Identified genomic interactions, often spanning multiple genes, linked thousands of predicted CREs with their putative targets and revealed notably frequent promoter-promoter contacts. Using the LEA gene family as an example, we discuss possible roles of these interactions in transcription regulation. On the *Vrn3* gene, we demonstrate the potential of our datasets to predict CREs for other developmental stages.

INTRODUCTION

Eukaryotic gene expression is regulated at multiple levels, with transcription initiation being a foundational level governed by a *cis*-regulome. This includes core promoters, binding RNA polymerase pre-initiation complex, and proximal and distal elements.[1] The distal *cis*-regulatory elements (CREs) can function as enhancers, silencers, or insulators. The ratio of proximal to distal CREs varies depending on the intergenic space, with larger genomes incorporating more distal, chromatin loop-mediated regulation.[2]

In this context, barley (*Hordeum vulgare* L.), one of the earliest domesticated crops, represents a good experimental model for small-grain temperate-zone cereals due to its diploid genome of almost 5 Gb.[3,4] Barley core promoters were analyzed in detail in our previous work,[5] but knowledge about the localization and function of proximal and distal CREs remains scarce. The barley genome has vast intergenic spaces, which are likely to be rich in distal CREs, as indicated by the distribution of epigenetic features associated with transcriptional activity.[6] Comprehensive studies, including whole-genome profiling of various epigenetic features, have been published for several cereal species, including for bread wheat,[7] maize,[8] and rice.[9] Such analyses, typically conducted using seedlings or leaves, provided insights into the general regulatory potential of CREs in terminally differentiated tissues. Given that *cis*-regulation is most intense during embryonic development,[10] current CRE collections are unlikely to be complete and need to be extended to include those from actively differentiating stages.

In contrast to promoters and proximal elements, located just upstream of transcription start sites (TSSs), distal CREs, lacking recognizable sequence signatures and residing at up to 1 Mb from their target,[11] are notoriously difficult to locate. Since their activity is closely linked to epigenetic features such as chromatin accessibility, histone modifications, and DNA methylation, the combination of these marks serves as a guide to their annotation. DNA methylation is a stable CRE characteristic in plants with many elements remaining unmethylated even outside of their activity time window.[12] In contrast, accessible chromatin regions (ACRs), identified by ATAC-seq, are more dynamic, reflecting immediate transcription factor (TF) binding, followed by histone H3/H4 acetylation, RNA polymerase association, and H3K4 methylation, as reviewed in Weber et al.[13] and Shlyueva et al.[14] Opposed to that, H3K27me3, a result of polycomb activity, decorates histones in facultatively repressed regions, as reviewed in Mozgova et al.[15] Collectively, specific combinations of histone

This is an open access article under the CC BY license (http://creativecommons.org/licenses/by/4.0/).

modifications, known as the histone code,[16] with ACRs and unmethylated regions are determinants of the current functional status of a given chromatin segment, efficiently integrated by computational methods based on machine learning.[17,18]

Only a handful of studies have comprehensively defined the CREs, including their targets.[8,19–22] When a CRE is distal to its target promoter, it establishes a transient physical interaction with the target gene via chromatin looping.[13,23–25] In polycomb gene silencing, H3K27me3-associated loops between genes and their enhancers, or "silencing hubs," gather genes and CREs to repress their activity during development.[26] Chromatin conformation capture (3C)-based techniques remain the only experimental methods to determine CRE target genes.[27] Their efficiency improves when combined with antibody subtraction (HiChIP) or promoter capture (Capture Hi-C),[28,29] as reviewed in Šimková et al.[30]

Chromatin loop mediation is one of the roles of unstable, small non-coding RNAs transcribed from animal enhancers (enhancer RNA [eRNA]).[31] Tens of thousands of these RNAs, transcribed uni- or bidirectionally, have been detected in mammalian cells, where they also facilitate chromatin remodeling and TF recruitment.[31,32] However, the existence and function of eRNAs in plants remain debated.[33] Plant CRE-associated RNAs are predominantly unidirectional, with bidirectional transcripts much less common compared to vertebrates.[34] Previously, we detected stable capped transcripts genome-wide in barley embryos by CAGE,[5] but the sensitivity for low-expressed, unstable RNA was insufficient, calling for a further study.

An increasing number of studies provide evidence of CREs overlapping with eQTL and their association with agronomically important traits in crops.[21,35] Understanding transcription *cis*-regulation during embryonic stages expands our knowledge of the establishment of valuable traits such as seed vigor and seed longevity.[36] LATE EMBRYOGENESIS ABUNDANT (LEA) proteins play a key role in these processes, as reviewed by Leprince et al.[36] Expressed in response to water loss during seed maturation as well as during vegetative growth, they contribute to desiccation tolerance and adaptation to drought stress.[37] LEA genes are evolutionarily conserved among angiosperms, with the LEA_5 family showing the highest conserved synteny, indicating evolutionary constraints on maintaining the integrity of their genomic context.[37] This, along with the clustered distribution of LEA_5 genes observed across the barley pangenome v2 collection,[38] highlights them as promising targets for investigating their interactome and epigenomic context.

Another fundamental process in plant development is the transition from the vegetative to the reproductive stage, regulated by vernalization-related genes and miRNAs, both developmentally and in response to environmental cues. The vernalization involves epigenetic mechanisms, including changes of histone modifications and 3D chromatin remodeling in winter wheat.[39,40] One of the crucial regulators, the product of *Vernalization3* (*Vrn3*), putatively orthologous to Arabidopsis *FLOWERING LOCUS T* (*FT*), functions as florigen in wheat.[41] Its expression is controlled not only by a vernalization but also by an age-related pathway, ensuring that flowering occurs in adulthood.[39] Two transcriptional enhancers were found upstream of *Vrn3* in winter wheat,[39] raising a question of whether the same CREs could control *Vrn3* transcription in spring barley.

Here, we predict the *cis*-regulatory landscape of the barley genome in the developing embryo, germinating embryo, and leaf. For each stage, we generated and integrated whole-genome profiles of several epigenetic features and complemented them by interactome data for the maturing embryo. Our study concluded with a comprehensive map of key epigenome features, predicted CRE candidates (cCREs), and genomic interactions. Analysis of these interactions assigned gene targets to multiple identified cCREs and revealed diverse interaction classes. Nascent RNA sequencing confirmed the minor role of non-coding transcription in CRE activity. On a cluster of LEA genes, we demonstrated interactions between promoters and discussed their role in transcriptional regulation. At the *Vrn3* locus, we illustrate the power of our datasets to predict distal CREs for other developmental stages. This integrative analysis advances the understanding of transcription regulation in large plant genomes and provides a valuable resource for bioengineering in barley. All data visualizations are available through an interactive genome browser (https://olomouc.ueb.cas.cz/en/resources/barleyepibase).

RESULTS

Profiles of epigenetic features enable the annotation of *cis*-regulatory elements

To enable comprehensive annotation of the barley *cis*-regulome, we followed a workflow outlined in Figure 1A. We selected a minimal set of epigenetic features and generated genomic profile datasets from three stages of barley embryo development—8 days after pollination (8DAP), 24 days after pollination (24DAP), and 4 days of germination (4DAG)—as well as from young leaf tissue. The high quality of our datasets was proven by standard profile distributions around annotated gene TSSs (Figure 1B) and documented through peak counts and replicate overlaps, summarized in Table S1. The active TSSs are enriched with histone modification marks and ATAC-seq signals, while being devoid of DNA methylation, as expected.[13]

A notable CRE feature that appears largely independent of cellular context is DNA methylation. We quantified the methylation levels in 24DAP embryos (Figures S1A and S1B) and leaf tissue (bisulfite sequencing [BS-seq] data for leaf are from[42]). Consistent with findings in other cereals, barley exhibits a highly methylated genome, with average genome-wide methylation levels of 88.6%, 58.1%, and 1.4% in the CpG, CHG, and CHH sequence contexts, respectively. We defined unmethylated regions (UMRs) in the 24DAP and leaf samples and subtracted all UMRs overlapping with genic regions, defined as described below, resulting in 102,287 and 102,362 UMRs, respectively. We then identified "permanent" UMRs as the overlap between these two sets, yielding a total of 74,614 intergenic UMRs.

As a cell-specific feature that serves as a useful proxy for functional sequences associated with transcriptional activity, we assessed open chromatin by ATAC-seq across all four stages (Figure S1C). Additionally, we immunoprecipitated chromatin using antibodies against three histone posttranslational modifications to capture actively transcribed regions (H3K4me3

CellPress
OPEN ACCESS

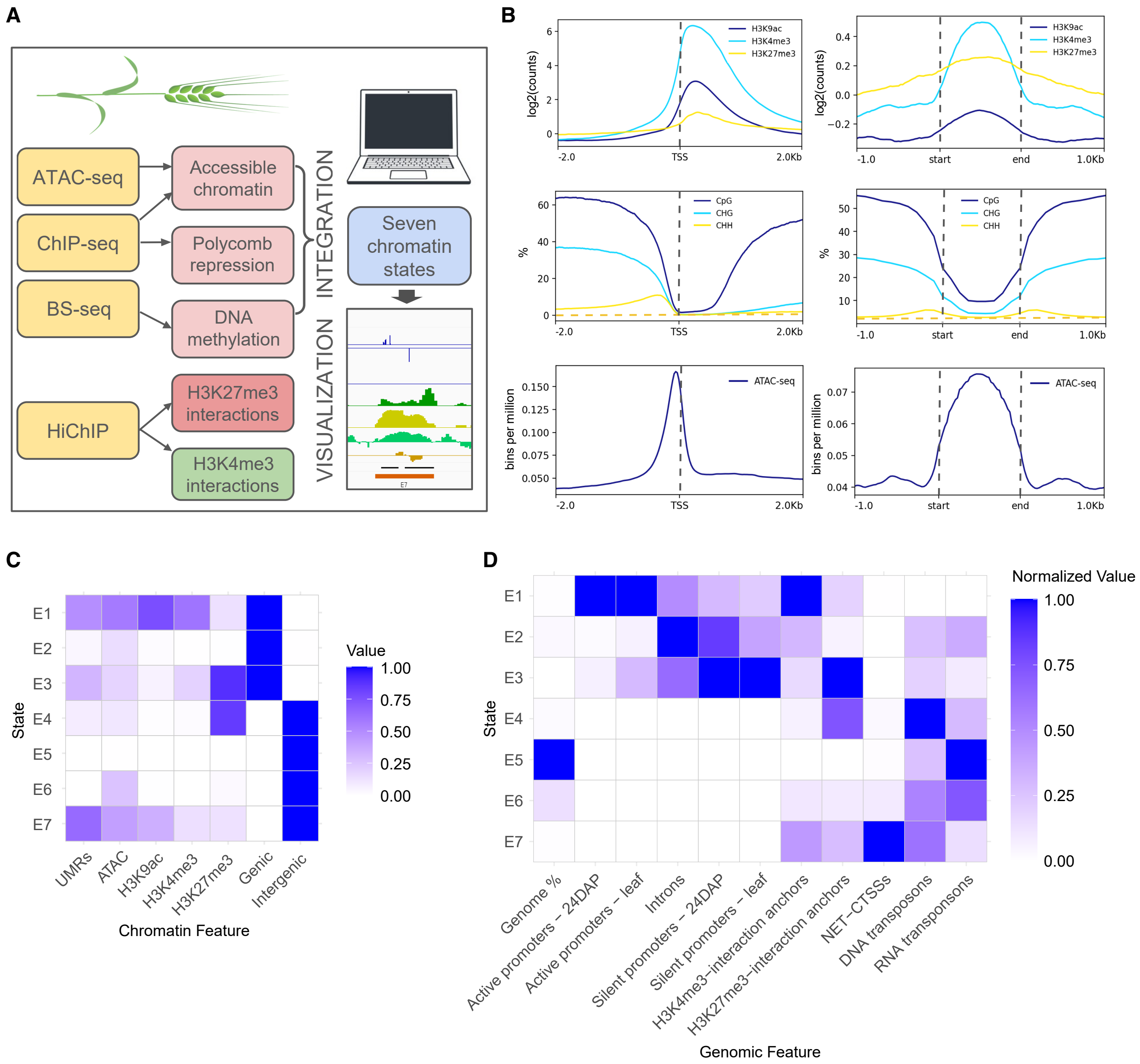

Figure 1. Key epigenetic chromatin features facilitate the annotation of *cis*-regulatory elements
(A) Components of the barley *cis*-regulome and interactome analysis.
(B) Profiles of coverages of key epigenetic features—key histone modifications (top), DNA methylation (middle), and open chromatin (bottom) around active TSSs (left) and across segments with chromatin state E7 (right) in the 24DAP embryo.
(C) A chromatin state emission values heatmap for the ChromHMM model distinguishing seven chromatin states. The intensity of the blue color corresponds to emission values, indicating the likelihood of a given state being associated with the chromatin feature.
(D) Overlap enrichment heatmap shows the fold enrichment of each state of the segmentation from (C) for a set of selected 24DAP genomic features. H3K4me3 and H3K27me3 interaction anchors correspond to 5-kb interaction bins from HiChIP analysis, while NET-CTSS corresponds to clusters of nascent capped transcript initiation used for eRNA detection.

and H3K9ac) and polycomb-repressed genomic regions (H3K27me3; Figure S2).

Importantly, to prevent the contamination of our *cis*-regulome analysis with unannotated genes (Figure S3) and to avoid false positives in defining CRE candidates, which resemble promoters in their epigenetic features, we carefully assessed the protein- and lncRNA-coding potential based on RNA-seq data and a previous publication.[43] Such transcribed regions, extended by 500 bp in both directions, along with the high- and low-confidence MorexV3 gene annotations, extended by 500 bp at the TSS, define the "genic" portion of the genome. The complementary genomic regions were classified as "intergenic," and both

categories, essential for the annotation of potential regulatory elements, were subsequently used to define the "coding potential" in the chromatin state model.

Discovering *de novo* the major recurring combinatorial and spatial chromatin patterns, known as "chromatin states," allows to narrow down the genome to putative regulatory elements. To achieve this, we integrated ATAC-seq, ChIP-seq, and UMR data from four stages of barley development with "coding-potential" segmentation and conducted chromatin state analysis using ChromHMM.[44] This approach binarizes the data and applies a multivariate hidden Markov model to learn a defined number of chromatin states. The resulting minimal model, which resolved both active and silent genic and intergenic situations at a 200-bp resolution, comprised seven chromatin states, referred to as E1–7 (Figure 1C). Following this, we conducted genomic feature overlap analysis for 24DAP (Figure 1D) and leaf (Figure S4A) samples to quantify normalized overlaps between individual states and various genomic features.

The resulting probability heatmaps confirmed the functional identity of genomic segments defined by the seven states and uncovered transcription-related dynamics of promoter states between stages. The active chromatin state E1, enriched in H3K4me3 and H3K9ac and characterized by open chromatin and UMRs, is predominantly associated with active gene promoters. In contrast, the repressed coding state E3 overlaps especially with polycomb-silenced promoters and genes, marked by H3K27me3. Importantly, our chromatin analysis identified largely unmethylated intergenic regions dominated by open chromatin and acetylated histones with a certain likelihood of histone H3K4 and H3K27 tri-methylations, corresponding to state E7, as confirmed by chromatin profile distributions across E7 segments (Figure 1B). Pool of 77,383 E7-state segments from four analyzed stages, likely enriched in active CREs, covers 1.43% of the barley MorexV3 genome. Stage-specific E7 segments (Table S2) were used for further analyses. The overlap of E7 segments across samples revealed their dynamics: 63.4% of all elements were commonly detected in all stages, whereas 2.4%–9.6% were stage specific (Figure 2A). The H3K27me3-enriched state E4 segments represent intergenic polycomb-silenced regions, with a mean size of 3.5 kb, compared to the 800-bp E7 segments. Approximately 76% of E4 segments embedded relatively small UMRs, with a mean size of 400 bp, suggesting their function in CRE silencing.

To narrow down a set of high-confidence active CRE candidates for each stage, we intersected stage-specific intergenic ATAC-seq peaks with UMRs and H3K9ac, as visualized by UpSet plots[45] (Figures 2B and S4C). The resulting stage-specific sets (Table S3), each covering ~0.1% of the MorexV3 genome, are referred to as "robust cCREs" and comprise 2,766–7,159 elements. The vast majority of the robust cCREs overlap with E7 segments, while subsets of the E7 segments lack an overlap with any of UMRs and ATAC-seq and H3K9ac peaks. These regions exhibit less pronounced profiles of all chromatin features characterizing CREs (Figure S4D), and therefore, we called them "weak E7." They point to the higher sensitivity of ChromHMM compared to peak calling in identifying regions with regulatory potential.

Evolutionary conservation of non-coding sequences is one of the key indicators for identifying regulatory elements.[46] To assess conservation of our predicted regulatory elements, we calculated the per-base sequence conservation—the PhastCons score, ranging between 0 and 1—from whole-genome multiple alignment of five grass species (*Hordeum vulgare*, *Triticum urartu*, *Secale cereale*, *Brachypodium distachyon*, and *Aegilops tauschii*). The average PhastCons scores of the robust cCREs and E7 elements were significantly higher (p values <2.2e−16, Wilcoxon test) compared with random genomic regions of the same size (Figure 2C) but still lower than those of exons, pointing to the functional importance of the predicted regulatory elements. However, this comparison could only be done for regions showing a certain level of sequence alignment, while non-aligning regions, which are scored as "missing data," are excluded from the analysis.[47] To give a more complete picture of the analyzed datasets, we also calculated average PhastCons scores for all cCREs/exons in the datasets by scoring the "missing data" as 0. In this calculation, the mean PhastCons scores were 0.274 for robust cCREs, 0.179 for E7 segments, 0.648 for exons, and 0.031 for the random genomic regions.

Another distinctive feature of CRE sequences is their enrichment in TF binding sites (TFBSs). We analyzed TFBS content in robust cCREs through motif enrichment analysis using RSAT,[48] identifying sets of developmental and hormone-responsive factor binding sites. At 24DAP, the ethylene-responsive factor (ERF) motif was the most significant, consistent with its overexpression in embryos and role in starch formation.[49] Other highly enriched motifs belonged to TFs crucial for embryonic development,[10] including Trihelix, HD-ZIP, MADS-family, bZIP, and B3 domain-containing LFL-like proteins (Figure 2D). In 8DAP-specific elements, the GAGA-motif binding TF BPC-like, required for seed development via homeotic TF regulation,[50] was highly significant (Figure S5A). In the 4DAG sample, the most significant motif was that of ERF (Figure S5B), followed by ZFHD10-3- and NAC factor-binding sequences. In leaf robust cCREs, ERF was again the most enriched, followed by Wuschel-like homeobox and B3 domain-containing protein (Figure S5C). These findings underscore the functional importance of the identified robust cCREs in barley growth and development.

Barley bidirectional and unidirectional unstable transcripts are an infrequent feature associated with cCREs

Active CREs may produce unstable transcription signals (eRNA), both unidirectional and bidirectional. To detect 5′ capped short unstable eRNA species in the 4DAG embryo, we utilized native elongating transcript–cap analysis of gene expression (NET-CAGE).[51] From the resulting data, summarized in Table S4, we identified 72,119 NET-CAGE tag clusters, with their dominant tags indicating positions of TSSs, which co-localized fairly well with the annotated gene TSSs positions (Figures 3A and 3C). The NET-CAGE cluster annotation (Figure 3D) defined 16,592 intergenic clusters. Filtering against the previously defined "genic" portion of the genome left us with 7,244 strictly intergenic NET-CAGE clusters. These were analyzed for transcript directionality, which merged part of the clusters into larger, bidirectional units. Among these enlarged 6,829 clusters, 5,160 were unidirectional

CellPress
OPEN ACCESS

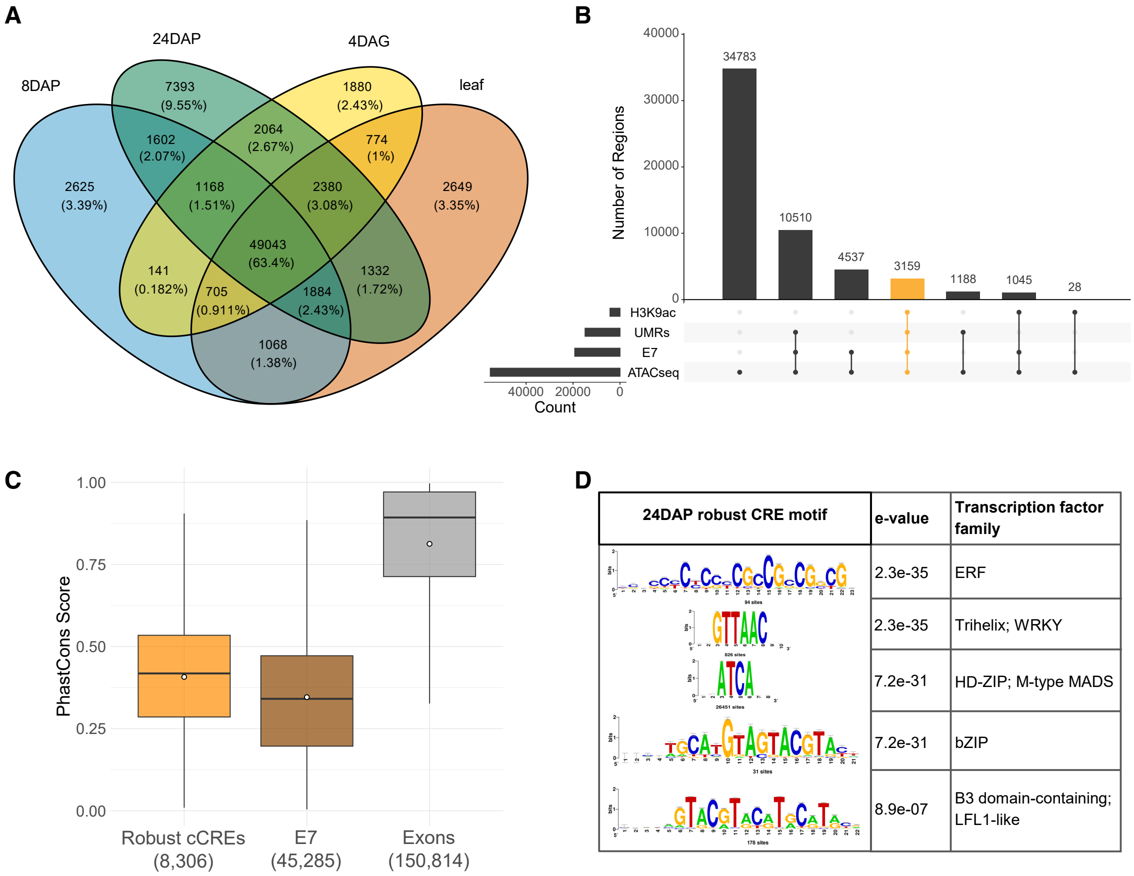

Figure 2. Characterization of candidate *cis*-regulatory elements

(A) Venn diagram showing the dynamics of E7 segments across four developmental stages—embryo at 8DAP, 24DAP, and 4DAG and young leaf tissue.

(B) The overlap of intergenic activating genomic features—peaks of ATAC-seq and H3K9ac and UMRs—defines the "robust cCRE" set (highlighted in yellow, shown here for the 24DAP embryo. At this stage, all robust cCREs overlap with E7 segments. "Count" indicates the total number of peaks for ATAC-seq while for E7, H3K9ac, and UMRs, overlaps of these datasets with ATAC-seq peaks are counted.

(C) Distribution of PhastCons scores (scale 0–1) across E7 segments, robust cCREs, and exons of high-confidence genes shows that both robust cCREs and E7 segments tend to be conserved across grass genomes. Significances of distribution differences compared to random genomic sequences were proved by the Wilcoxon test (all p values $<2.2e-16$). White dots indicate mean values for each set.

(D) Transcription factor binding sites enriched in the 24DAP-specific "robust cCREs" dataset.

(U), 1,669 bidirectional (B), and only 595 clusters were bidirectional and balanced—a configuration attributed to canonical eRNA in animals. The remaining B clusters appeared unbalanced (BU), with tags per million (TPM) values different for the signal pairs (Figure 3B). We found that particularly, the BU and U NET-CAGE clusters were highly overrepresented (randomization test) in our robust cCRE set as well as in state E7, unlike in the E4 genomic segments (Figure 3E). Nevertheless, the 4DAG NET-CAGE signal was observed only in ~9% (634/7,159) 4DAG robust cCREs and 3% (1,748/60,605) E7 segments, suggesting that the transcription of eRNA is not a typical attribute of barley CREs.

Taking advantage of the single-base precision of the dominant TSS positions, we addressed sequences flanking transcription initiators within the 7,244 strictly intergenic clusters. Using the seqArchR clustering algorithm, we identified four distinct sequence architectures at the TSSs (Figure 3F) and contextualized them with interquantile width (IQW) values, which are a measure of promoter width, TPM values, and the three directionality groups. The first cluster (C1) resembles coding-gene core promoters with a canonical CA initiator and a hint of a TATA-box approximately 35 bp upstream but exhibits TPM values < 1. This suggests that these may be promoters of lowly expressed unannotated genes or enhancers with a promoter-like architecture. The second most abundant cluster (C2) resembles a promoter starting from a non-canonical G-initiator while lacking other conserved motifs. The remaining two clusters (C3 and C4) represent low-complexity, microsatellite-like sequences. These clusters show a slightly higher tendency for bidirectional transcription compared to the promoter-like clusters, which are predominantly transcribed in one direction.

Histone modification-centric chromatin conformation capture detects interactions of active and silent regions

To identify spatial chromatin interactions indicative of contacts between both active and silent genes and their CREs, we

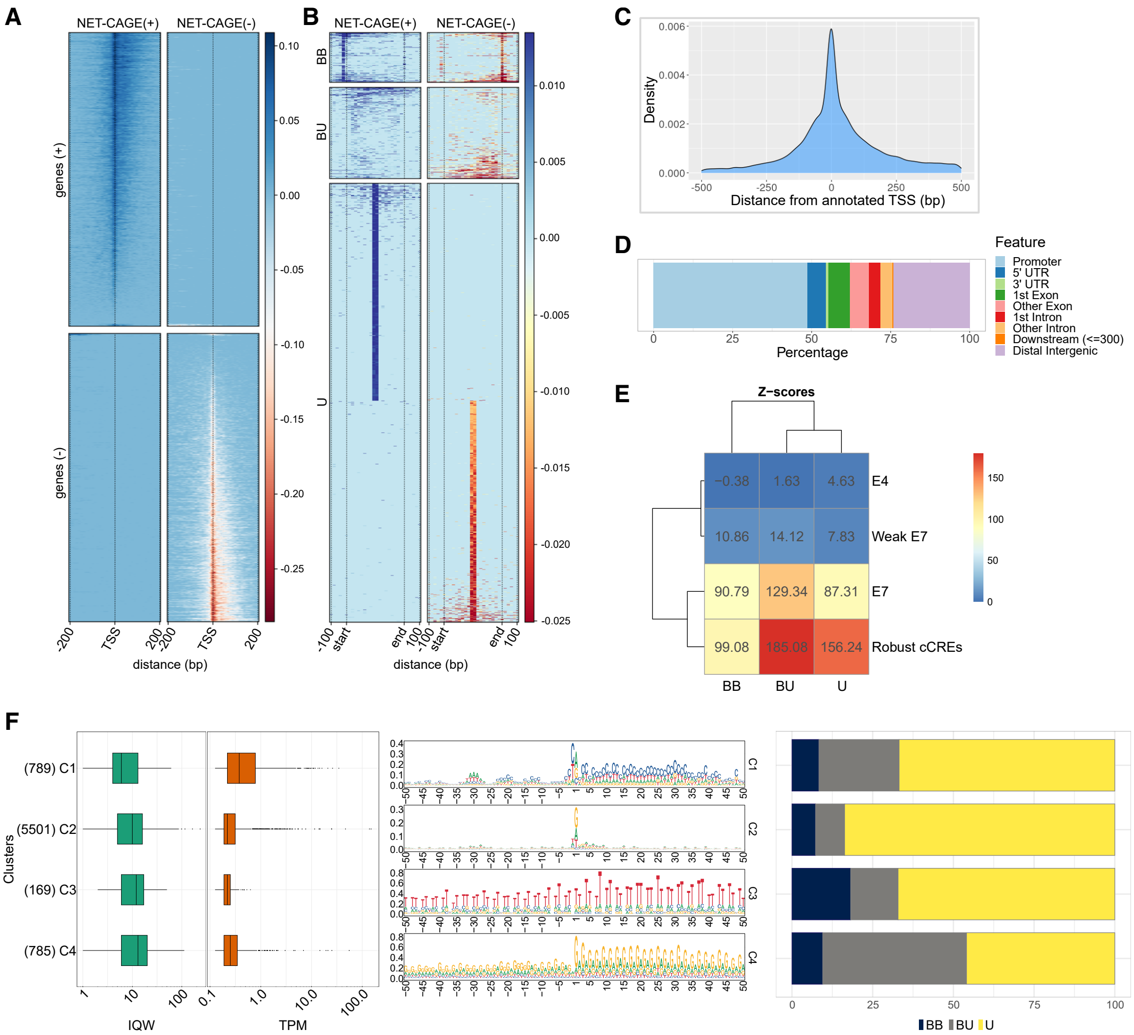

Figure 3. Features of native non-genic transcripts identified by NET-CAGE in the 4DAG embryo

(A) Heatmap of NET-CAGE cluster distributions on both DNA strands around annotated TSSs, indicating the absence of upstream antisense RNA signals. The heat map shows the amount of transcription signals. Transcription from the (+) and (−) DNA strands is represented by positive and negative values, respectively.

(B) Heatmap showing three directionality patterns of the non-coding RNA (ncRNA): BB, bidirectional balanced; BU, bidirectional unbalanced; U, unidirectional. For the bidirectional transcripts, the start/end relates to genome segments delimited by a pair of dominant TSSs from the opposite strands. The most abundant category consists of unidirectional transcripts resembling promoters of protein-coding genes.

(C) Distribution of dominant NET-CAGE tags around annotated (MorexV3) gene TSSs.

(D) Genomic feature annotation of NET-CAGE clusters.

(E) Overlap of the three ncRNA-directionality patterns with E4, E7, and "weak E7" segments and robust cCREs. The resulting Z-scores from the randomization test of feature overlap reveal a strong overrepresentation of ncRNA in the robust cCRE and E7 sets.

(F) Four major clusters of sequence architectures (cluster sizes in brackets), together with interquantile width (IQW) and tags per million (TPM) values. Dark blue, gray, and yellow bars show the representation (%) of the directionality patterns in each cluster.

performed HiChIP using anti-H3K4me3 and anti-H3K27me3 antibodies. The experiments were conducted on G1-phase nuclei prepared from 24DAP embryos in two replicates, which yielded highly correlating data (Figure S6A). The HiChIP data mapping rate and HiChIP signal enrichment at ChIP-seq peaks indicated good sample quality (Figures S6B and S6C). The resulting valid interaction pool was analyzed using FitHiChIP[52] to identify pairs of regions ("anchors") with a significant number of reads mapping between them, representing biologically meaningful chromatin interactions (Figures 4A and 4B). The FitHiChIP "peak-to-all" mode enabled the identification of cCREs lacking H3K4me3/H3K27me3 that interacted with marked promoters. For H3K4me3 at the 5-kb resolution, which is the dataset used for the majority of analyses, the average and median interaction distances are 119 and 60 kb, respectively. At these distances, the genomic segments between the anchors often contain unrelated gene(s). The number of spanned genes (Figure 4C) cautions against the assumption that the cCREs typically regulate the neighboring gene. We also counted the number of interactions associated with a single promoter and found that one

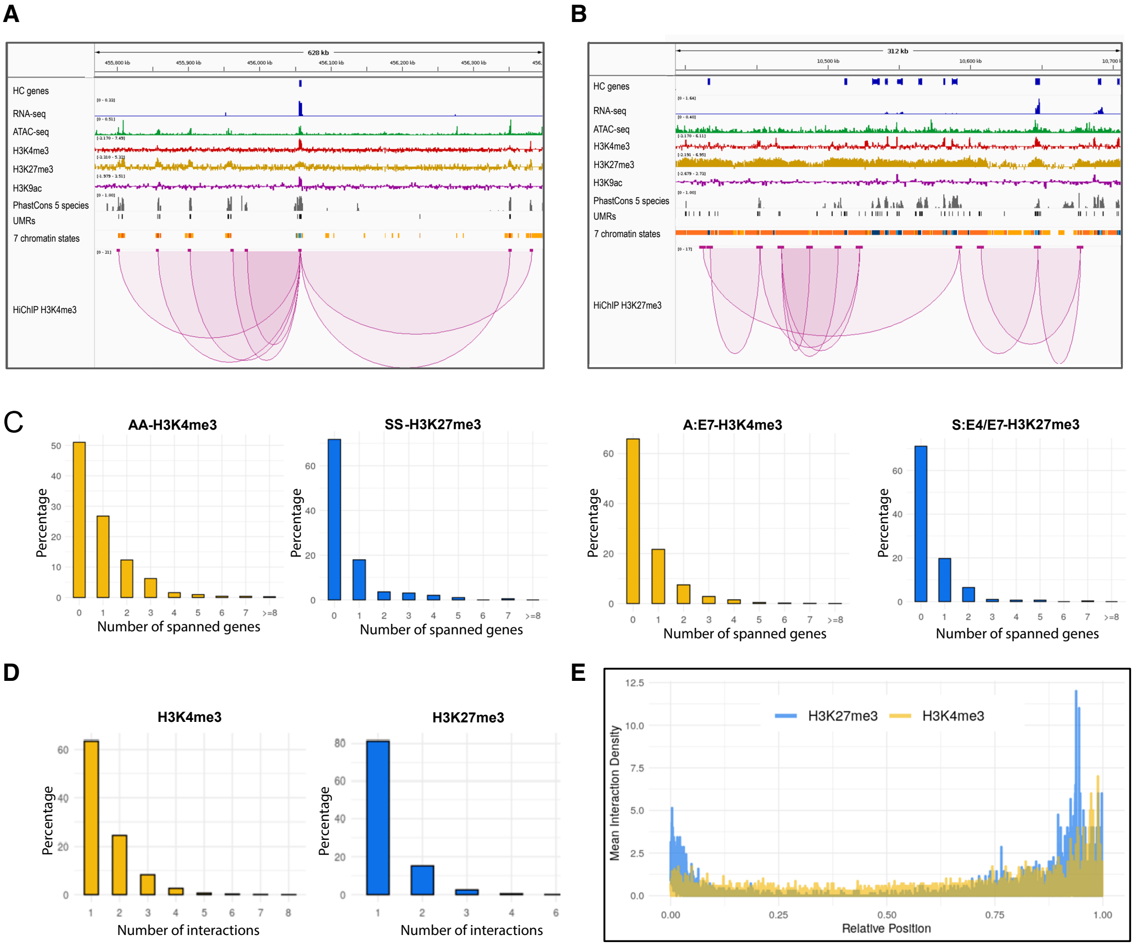

Figure 4. High-resolution analysis of the interactome in the 24DAP embryo

HiChIP data enriched in activating (H3K4me3) or silencing (H3K27me3) histone marks were analyzed at 5-kb resolution.

(A and B) Examples of multiway HiChIP interactions involving (A) an active promoter and (B) a polycomb-silenced region.

(C) Numbers of genes spanned by specific types of interactions: Active promoter-active promoter (AA), silent promoter-silent promoter (SS), active promoter-E7 state segment (A:E7), and silent promoter-E4 or E7 segment (S:E4/E7).

(D) Numbers of interactions per active promoter (H3K4me3 interactions) and per silent promoter (H3K27me3 interactions).

(E) Generalized chromosomal distribution of equal numbers of H3K27me3 and H3K4me3 interactions.

promoter can interact with up to eight targets (Figures 4A and 4D). At lower resolutions, both the number of genes spanned by an interaction and the numbers of significant interactions per promoter (Table S5) increase (Figures S7A and S7B), because low-resolution analyses cannot resolve short distance and favor longer-distance contacts. The chromosomal distribution of all interactions was skewed toward gene-rich sub-telomeric regions (Figures 4E; S7C), as expected. Interestingly, the enrichment in H3K27me3 interactions was higher than that in H3K4me3 interactions, possibly due to the prevalence of genes regulated by polycomb in distal chromosomal regions, related to chromosome partitioning.[53]

Annotation of genomic interactions determines the composition of the primary interaction classes

To gain a deeper understanding of these interactions and the promoters involved, we hierarchically annotated the interaction datasets. Given that H3K4me3 and H3K27me3 predominantly mark active and silent promoters, respectively, these two genomic features were given top priorities in the annotation, followed by other genic features as terminators and introns. Chromatin states E7 and E4, including cCREs and intergenic polycomb regions, respectively, ranked further down in the annotation hierarchy, completed with TEs. We identified diverse genomic feature pairs at anchors, defining distinct classes of interactions (Figure S8). Quantification of all possible interaction class proportions (Figure S9; Table S6) and those involving only active promoters (Figure 5A) revealed that H3K4me3 HiChIP primarily captures interactions between two active promoters (25.8/31.7% for all-interactions and active-promoter-centric analysis, respectively) or active promoter-E7 interactions (22.6/27.8%). Additionally, 7.95% of active promoters interact with introns and 5.22% with silent promoters. For H3K27me3 silent promoter-centered interactions (Figure 5B), E4 and E7

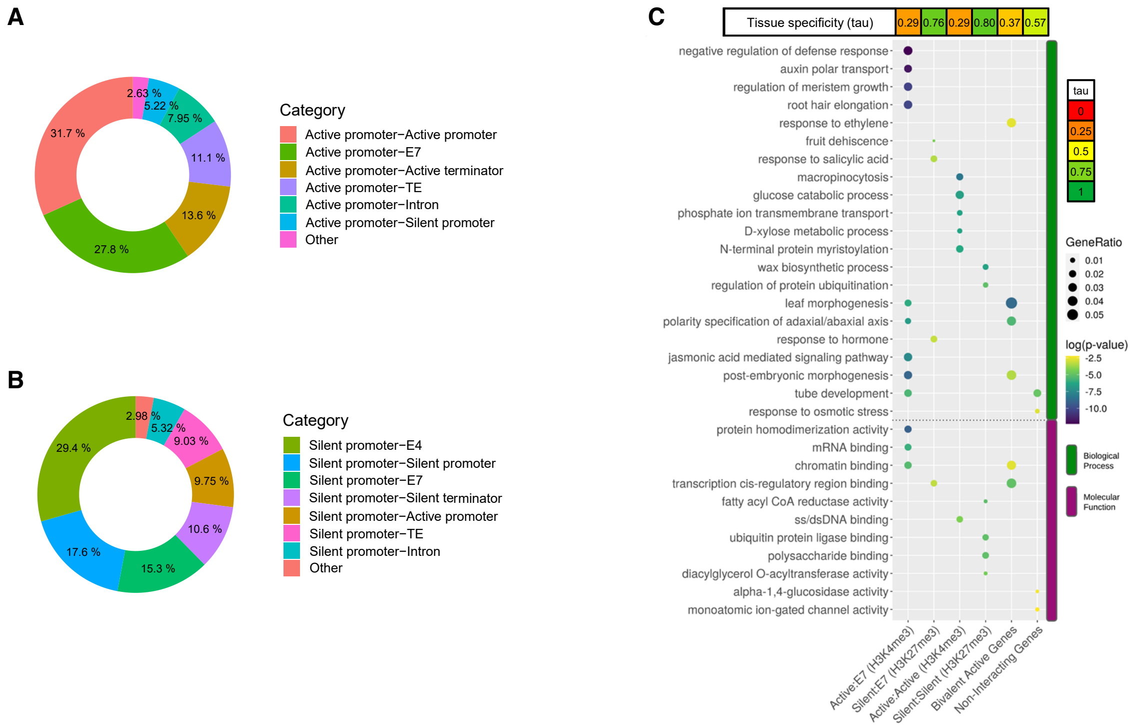

Figure 5. Annotation of HiChIP interactions in the 24DAP embryo

(A) Annotation of significant active promoter-centered (H3K4me3) interaction classes.

(B) Annotation of significant silent promoter-centered (H3K27me3) interaction classes. Active promoters, silent promoters, terminators, introns, CRE candidates, and transposable elements were used in this order as genomic features for hierarchical annotation. "Other" includes all interactions that do not exceed 5% of the total.

(C) GO term enrichment and tissue specificity analysis of genes involved in specific classes of interactions: Active:E7 (H3K4me3) = active promoter-E7 genomic segment, H3K4me3 interactions; silent:E7 (H3K27me3) = silent promoter-E7 genomic segment, H3K27me3 interactions; active:active = active promoter-active promoter; silent:silent = silent promoter-silent promoter.

interactions account for 29.4% and 15.3%, respectively, while silent promoter-silent promoter interactions make up 17.6%. We also identified a category of long self-looped genes (Figure S8B), comprising 232 active and 137 silent promoter-terminator pairs within the same gene.

Pairs of interacting genes tend to be co-expressed

Given that about a third of the H3K4me3 interaction set comprises interactions between two active genes (Figure S9A; Table S6), we investigated whether these interactions reflect the physical proximity of genes that are co-regulated during plant development. To test this hypothesis, we assessed whether gene expression changes between stages are more concordant within these interaction pairs than expected by chance. We assigned interacting genes to expression clusters generated by k-means clustering from public gene expression datasets[54,55] and evaluated the significance of both interaction partner genes falling into the same cluster. Out of 1,988 active promoter-active promoter interacting pairs with a single-gene annotation in each interacting bin, 502 pairs belonged to the same expression cluster. The low p value of this analysis (chi-square test, 4.807e−09) indicates that interacting genes are significantly more likely to belong to the same expression cluster than would be expected by random chance, implying their co-expression and co-regulation. Gene Ontology (GO) analysis suggests that products of these genes play roles in fundamental cellular processes, such as metabolism of monosaccharides and ss/dsDNA binding (Figure 5C).

Promoter-CRE interactions and bivalent chromatin predominantly involve genes encoding TFs

GO annotation of genes involved in active promoter-E7 interactions revealed that this group is dominated by genes encoding proteins involved in morphogenesis, defense response—known to be associated with seed maturation[36]—and chromatin binding that have low tissue specificity (Figure 5C), consistent with the known pleiotropic and multifunctional nature of developmental TFs.[56] To support this observation, we compared genes interacting with E7 segments to a group of non-interacting expressed genes. The first group overlapped significantly more than expected with a set of 2,060 barley developmental TFs defined by[55] (p value = 0.00356, hypergeometric test), whereas the non-interacting group showed no overlap (p value = 0.99). This provides additional evidence that TF genes, in particular, are targets of distal CREs, similar to findings in animals.[57]

Polycomb-silenced genes form loops with other silent promoters as well as E7 and E4 segments. GO term analysis of silent gene-E7 interactions also pointed to TFs (DNA binding

CellPress
OPEN ACCESS

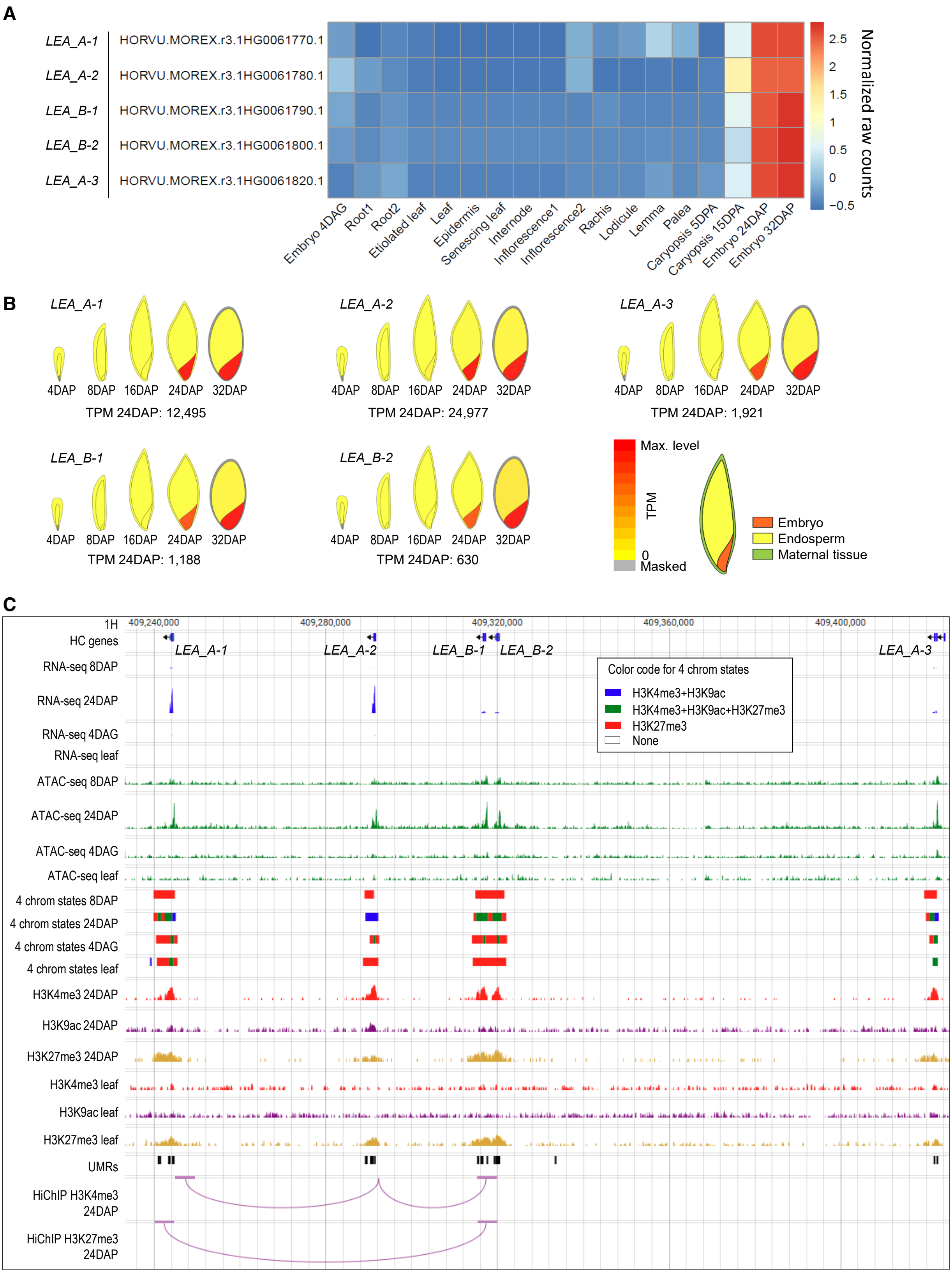

(legend on next page)

cis-regulatory activity, response to hormones); however, this set of genes appears highly tissue specific (values ~0.8). Additionally, silent promoter-silent promoter H3K27me3 loops were associated with tissue-specific genes, although these genes were clearly linked to metabolic functions. Thus, polycomb loops may act as important determinants of developmental and tissue-specific regulation.

We further focused on a subset of 355 interactions where both anchors simultaneously overlapped in H3K4me3 and H3K27me3 HiChIP datasets at 5-kb resolution. Interestingly, these interactions involve both active and silent genes. The largest group (97; 27.3%) of these bivalently marked interactions involve active promoters interacting predominantly with the E7 class (Figure S10). This group includes tissue-unspecific genes functionally annotated as TFs, enriched in GO terms such as morphogenesis and chromatin binding (Figure 5C).

Late embryogenesis abundant genes interact and are co-regulated

To exemplify the dynamics of the epigenomic landscape and its impact on gene transcription, we focused on a cluster of genes encoding late embryogenesis abundant proteins from the LEA_5 group. This cluster consists of two groups of paralogous genes (Ensembl Plants)[58]: *HORVU.MOREX.r3.1HG0061770* (named *LEA_A-1*), *HORVU.MOREX.r3.1HG0061780* (*LEA_A-2*), and *HORVU.MOREX.r3.1HG0061820* (*LEA_A-3*), forming the paralogous group LEA_A, and *HORVU.MOREX.r3.1HG0061790* (*LEA_B-1*) and *HORVU.MOREX.r3.1HG0061800* (*LEA_B-2*), forming the group LEA_B (Figure 6). While these genes exhibit negligible or no transcription in most tissues analyzed by Mascher et al.[53] or Kovacik et al.[55] (Figures 6A and 6B), they show high-to-ultrahigh transcription levels (630–24,977 TPM) in the maturing embryo at 24DAP and 32DAP[55] (Figure 6B). Despite these differences, all LEA genes in the cluster share a similar epigenetic landscape during development. Genic and adjacent regions are unmethylated, with minimal differences between 24DAP embryo and leaf tissue. The burst of transcription at 24DAP is preceded by chromatin opening in promoter regions, which is already apparent in the 8DAP embryo (Figure 6C). Later in embryo development, part of the silencing H3K27me3 marks loaded on broader genic regions is replaced by activating H3K4me3 and H3K9ac marks. This process is gradually reverted during seed germination and further development, concurrent with transcription silencing (Figure 6C). Collectively, the dynamics of epigenomic features across four stages indicate that the ratio of activating versus silencing histone marks in a gene promoter region is the strongest determinant of transcription activity in these developmentally regulated genes.

Exploration of H3K4me3-enriched interactions for the 24DAP embryo indicated that coordination of epigenetic changes, and consequently transcription within the *LEA* cluster, could be mediated by chromatin interactions involving the genes. Analysis at 5-kb resolution revealed highly significant contacts between the centrally located *LEA_A-2* gene and its closest neighbors, while no contact was identified between the most distal and the least expressed of the LEA_A paralogs—*LEA_A-3* (Figure 6C). Besides the H3K4me3-mediated contact, the downstream region and/or gene body of *LEA_A-1* is connected through an H3K27me3-mediated interaction with the paralog pair *LEA_B-1* and *LEA_B-2*, confirming spatial contacts of the clustered genes. Interestingly, *LEA_A-2*, occupying a central position in the local interactome, has the highest transcription level (24,977 TPM; Figure 6B), suggesting a potential positive effect of the spatial organization of the *LEA* locus on the gene's transcription. This hypothesis is supported by the fact that promoter regions of *LEA_A-2* interactors—*LEA_A-1*, *LEA_B-1*, and, possibly, *LEA_B-2*—contain binding sites for ABI5 homolog (*HORVU.MOREX.r3.3HG0300770*, motif ACGTGTC), a bZIP TF known to regulate LEA genes,[36] while no such site was identified by the PlantTFDB prediction tool[60] within 1,500 bp upstream of the TSS of *LEA A-2*, as identified by CAGE.[5] Experimental validation will be required to confirm the potential collaboration of these promoters in transcription regulation.

Regulatory elements of *Vrn3* are detectable in non-expressing tissues and without vernalization treatment

To test the predictive power of our data, we attempted to identify previously described enhancers using our datasets. Recently, two enhancers of *Vrn3*, located within 30 kb upstream of the gene, were identified in winter wheat through differential analysis of open chromatin after vernalization treatment.[39] Here, we used spring barley cv. Morex, which does not require vernalization to flower, and explored the epigenetic landscape and interactome in the upstream region of barley *Vrn3* ortholog, *HORVU.MOREX.r3.7HG0653910*. Despite using embryonal and leaf tissues, with no (embryo) or minimal (leaf) transcription, we were able to predict four cCRE regions, showing an overlap of evolutionary conserved sequences with chromatin features corresponding to active or silenced CREs, within 330 kb upstream of the gene (Figure 7A). A cCRE located 242 kb upstream of *Vrn3*, designated cCRE3, exhibited a high-confidence (FDR <0.05, 5-kb resolution) H3K27me3-associated interaction with the gene. A BLAST search for the published wheat enhancers P3 and P4[39] provided hits overlapping with barley cCRE3 and cCRE4, respectively (Figures 7B and 7C; Figure S11). These CRE candidates are characterized by a bivalent epigenetic state, just like the *Vrn3* promoter, suggesting that this developmental gene is primed but remains silenced until it receives the final trigger

Figure 6. Transcription, epigenetic landscape, and chromatin interactions in a cluster of barley LEA_5 genes

(A and B) Results of transcriptomic analysis displayed as (A) differential analysis heatmap showing normalized raw counts for five LEA genes across 18 barley tissues (data from Mascher et al.[53] and Kovacik et al.[55]), and (B) expression values of the LEA genes across tissues of five stages of a developing barley grain (data from Kovacik et al.[55]; visualization from barley ePlant database[59]).

(C) Visualization of the gene cluster in a genome browser, integrating RNA-seq data[53,55] and information about open chromatin (ATAC-seq), histone modifications (ChIP-seq and four chromatin states generated by ChromHMM[5]), and unmethylated regions (UMRs). H3K4me3- and H3K27me3-enriched chromatin interactions (HiChIP) were analyzed at 5-kb resolution.

CellPress
OPEN ACCESS

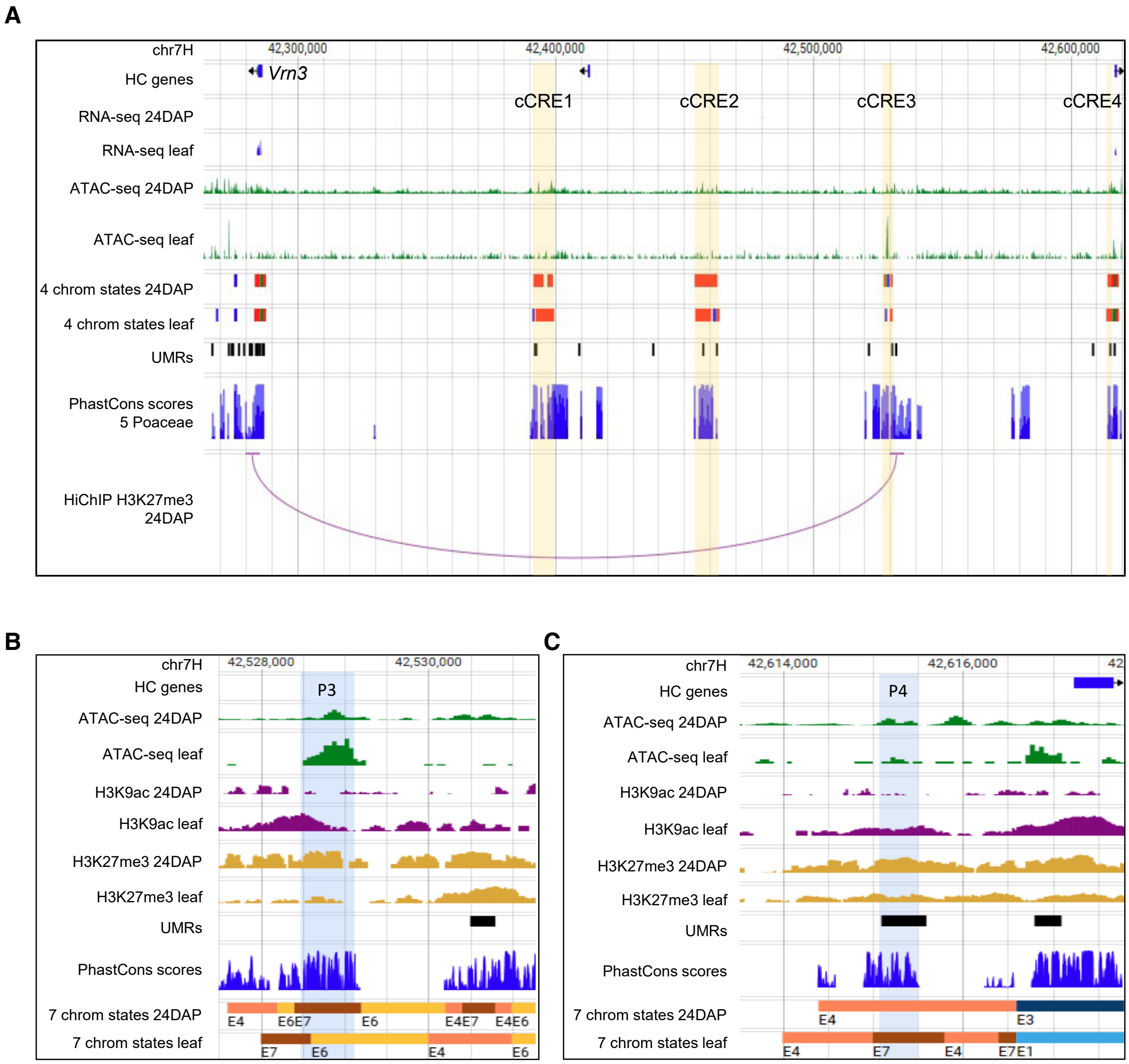

Figure 7. Regulome of the barley *Vernalization3* gene

(A) Barley *Vrn3* (*HORVU.MOREX.r3.7HG0653910*) locus with cCRE regions (yellow bars), predicted based on an overlap of evolutionary conserved sequences (PhastCons scores) with chromatin features associated with active or silenced CREs. Note the long-range (242-kb) contact between *Vrn3* and the cCRE3 region. The color code for four chromatin states is the same as in Figure 6.

(B and C) Zoom-in views of the cCRE3 (B) and cCRE4 (C) regions with BLAST hits of bread wheat P3 and P4 enhancers[39] (blue bars).

for expression, likely through the age-related pathway involving SPL7/15.[39]

Dataset visualization in public databases enables application in barley research

Our comprehensive analysis of barley epigenomic landscapes and chromatin interactions highlights the functional significance of CREs in regulating transcription during development. The resulting barley cCRE and interactome collections, together with profiles of several epigenetic marks and evolutionary conservation, complemented by published transcriptomic data,[5,53,55] all based on the MorexV3 genome,[61] serves as a valuable resource for other researchers. To ensure accessibility, we made all processed data files and their visualizations available in a JBrowse interface through the Elixir platform (https://olomouc.ueb.cas.cz/en/resources/barleyepibase).

DISCUSSION

Earlier studies on cCREs within large cereal genomes identified their hallmarks, including a high degree of chromatin openness, histone acetylation, and low DNA methylation levels.[7,8,12] Building on this, we generated datasets of these epigenetic features for barley and integrated them using an overlap-based and a machine-learning approach. Comparison of resulting datasets demonstrated that peak overlap defines a narrower, robust cCRE set, while the ChromHMM approach offers a broader, more informative classification, overcoming limitations due to

variable quality of the integrated datasets.[62] To maximize cCRE identification while maintaining analytical simplicity, we selected a 7-state ChromHMM model, which identified 1.43% of the barley genome as having regulatory potential (E7 chromatin state), consistent with 1.5% reported for wheat seedlings.[7] The H3K27me3-enriched E4 state, reflecting intergenic polycomb-mediated silencing,[26] may contain inactive CREs often escaping detection, manifested as small UMRs embedded in the E4 blocks. The occasional presence of H3K27me3 in E7 (Figure 1C) indicates that E7 segments may include not only active cCREs but also elements in transitional or binary states, as well as those active in some cell types but repressed in others within the sample. Benchmarking chromatin state revealed that E7 could be further divided into sub-states, providing deeper insight into regulatory element dynamics. While E7 dynamics across the four barley stages may appear limited (Figure 2A), the large number of commonly detected elements aligns with the number of commonly active genes in transcriptome analyses, reflecting pleiotropy and combinatorial dependence of enhancers.[63,64] The cCREs predicted in our study, both the E7 segments and robust cCREs, are relatively large genomic segments, mostly in the range of hundreds of base pairs to several kilobases, typically containing a series of predicted TFBSs. Exploring the cCREs in the context of sequence conservation, facilitated by the multiple sequence alignment (MSA) for Triticeae species integrated in the generated genome browser (Figure S11), helps to identify cCREs or their parts that have the highest regulatory potential. Nevertheless, the functionality of the predicted elements has yet to be experimentally proved by a reporter assay or targeted editing, as described below.

The largest comparative study of unstable RNA across diverse plants and vertebrates by capped small RNA sequencing[33] found that unstable and bidirectional transcription is rare in plants. In contrast, Xie et al.[34] detected active transcription of thousands of predicted enhancers in the bread wheat genome using two independent techniques (pNET-seq and GRO-seq). Their observation that genes associated with transcribed enhancers are expressed at significantly higher levels lead them to elevating the predictive power of eRNA above other chromatin features. Using a yet-different technique, NET-CAGE, we failed to detect many bidirectionally transcribed cCREs or upstream antisense RNAs, common in animals. Still, a small subset of robust cCREs and E7 elements overlapped with unbalanced or unidirectional nascent capped RNA. Interestingly, transcripts belonging to cluster C4 (Figure 3F) initiated from low-complexity GA-microsatellite regions, which are described in wheat as BPC5/Ramosa TFBS and are overrepresented in wheat distal CREs.[7] Overall, we found for barley far less transcripts in cCREs (1,748 NET-CAGE clusters in E7 segments) than reported for wheat (12,687 and 11,484 TSS clusters in predicted enhancer regions,[7] identified by pNET-seq and GRO-seq, respectively[34]), though the lower number partly reflects the 3-fold smaller size of the barley genome. Our findings suggest that enhancer transcription plays a minor regulatory role in barley, consistent with the findings of McDonald et al.[33] While methodological differences among studies might contribute to these discrepancies,[65] they may also reflect fundamental differences in transcriptional regulation mechanisms between plants and animals[66] worth further exploration.

Previous *cis*-regulation studies without 3D structural assays often inferred CRE targets based on proximity or expression levels of neighboring genes. However, our counting of genes interposed between interacting partners demonstrates that this assumption can lead to false conclusions. Also, gene expression level alone may reflect the gene promoter activity rather than up-regulation by CRE(s). 3C-based techniques can discern enhancer-promoter interactions but require high resolution, which is challenging to reach in complex tissues and repeat-rich genomes with low mappability, such as that of barley. Our analysis at 5-kb resolution hindered the precise annotation of gene-proximal and intronic CREs. While hundreds of intronic CREs were observed in our study, consistent with findings in the human genome,[67] they are not presented due to potential gene-type bias. The resolution also affects interaction distances. The median distance of H3K4me3 barley HiChIP loops at 20-kb resolution (220 kb) is much greater than that at 5 kb (60 kb) but is consistent with results from wheat low-resolution RNA polymerase II-HiChIP (200–400 kb).[19] Notably, the long-distance interactions may reflect structural rather than functional loops, akin to topologically associating domains.

Our HiChIP data identified bivalent interactions of chromatin segments simultaneously marked by both the activating H3K4me3 and repressive H3K27me3, occurring typically at developmental regulatory genes that are poised for activation or silencing in mammals.[68] In plants, evidence of bivalent chromatin is emerging in both developmental and stress-responsive genes.[69,70] Our GO term and tissue-specificity analyses of interacting genes (Figure 5C) indicated that H3K27me3 silences genes for tissue-specific TFs via their interactions with intergenic polycomb regions, while pleiotropic developmental TFs engage in binary interactions. However, distinguishing true bivalent chromatin, with both marks on the same nucleosome, from allelic or sample heterogeneity remains challenging, requiring sequential ChIP for confirmation.[71]

We unveiled a rich promoter-promoter interactome, which is indicative of co-expression hubs and TFs, reported in other plant species.[19,72,73] Zhu et al.[72] proposed a model of active transcription hubs that unifies the roles of active promoters and enhancers, assigning general enhancer-like functions to active promoters, which was also supported by other studies.[74,75] Our exploration of TFBSs in promoters of interacting LEA genes led us to hypothesize that the ultrahigh transcription of *LEA_A-2* gene, positioned centrally in the local interactome, might be due to the enhancer-like function of the interacting promoters, bringing ABI5 TFs, whose binding site is missing in the *LEA_A-2* promoter. Alternatively, the promoter of *LEA_A-2* may be the core regulatory element in the gene cluster, supporting the transcription of its interacting partners. In this scenario, the trigger of the transcription burst would not be ABI5 but another upstream trans regulator. Analysis of structural variation in promoter regions of the interacting *LEA* genes across multiple genotypes and their correlation with transcription levels may shed light on this phenomenon.

Our investigation of the *Vrn3* region demonstrated the potential of our datasets to show signatures of distal CREs even in

non- or low-expressing tissues and without the environmental stimulus that led to their identification in winter wheat.[39] It also indicated a relationship between these enhancers and the age-related pathway in spring barley. A silencing long-range interaction in barley *Vrn3* region revealed a spatial contact between one of our cCREs and *Vrn3*, particularly remarkable given the 10-fold greater distance between them in barley than in wheat.[39] A study using ChIA-PET for H3K4me3[40] found no significant chromatin loop in the *Vrn3* region after vernalization in winter wheat. The authors suggested that this might reflect higher enrichment in H3K27me3 than H3K4me3 in the locus, which aligns with our findings and confirms the region's bivalent chromatin state.

To validate CRE candidates, large-scale enhancer activity assays such as massively parallel reporter assays or self-transcribing active regulatory region sequencing (STARR-seq) modified for plants (Plant STARR-seq[76,77]) could be used to assess enhancer functionality and promoter-enhancer compatibility. However, testing elements outside their genomic context may yield inconclusive results, necessitating targeted editing by CRISPR-based techniques within the native loci for definitive validation. Since transcriptional changes drive a range of advantageous traits, CREs hold significant promise for trait engineering. Advances in machine learning, neural networks, and applications of large language models[78,79] enable to leverage high-quality datasets, such as those generated here, for model training. These tools enhance understanding of plant regulomes and support their biotechnological applications.[80]

RESOURCE AVAILABILITY

Lead contact

Requests for further information and resources should be directed to and will be fulfilled by the lead contact, Hana Simkova (simkovah@ueb.cas.cz).

Materials availability

The study did not generate any new reagents.

Data and code availability

The datasets generated during this study include outputs from ATAC-seq (four stages), ChIP-seq (leaf), NET-CAGE-seq (4DAG embryo), HiChIP (24DAP embryo), and BS-seq (24DAP embryo). Raw data have been deposited at the SRA: PRJNA1177611 and are publicly available as of the date of publication. In addition, processed datasets derived from these data are available for download and visualization in the Jbrowse interface at https://olomouc.ueb.cas.cz/en/resources/barleyepibase. Supplementary information relating to the article and original codes used in the bioinformatic analyses are deposited at the GitHub repository: https://github.com/MorexV3CAGE/Barley_distal_regulome and Zenodo: https://doi.org/10.5281/zenodo.14723229.

ACKNOWLEDGMENTS

The project was supported by the Czech Science Foundation (grant no. 21-18794S) and from the project TowArds Next GENeration Crops, reg. no. CZ.02.01.01/00/22_008/0004581 of the ERDF Programme Johannes Amos Comenius. Computational resources were provided by the e-INFRA CZ project (ID:90254), supported by the Ministry of Education, Youth and Sports of the Czech Republic. We thank Jitka Weiserova for technical assistance in flow sorting; Zdenka Bursova for plant maintenance; and Katerina Holusova, Helena Tvardikova, Pascal Jaroschinsky, Axel Himmelbach, and Jörg Fuchs for their assistance with library preparation and sequencing. The work performed at IPK Gatersleben was part of the p-epBAR project, supported by the German Ministry of Education and Research (BMBF) (grant no. FKZ 031B1224). Jbrowse genomic browser is provided by ELIXIR-CZ Research Infrastructure Project (LM2023055).

AUTHOR CONTRIBUTIONS

H.S., P. Navratilova, and N.S.: project conceptualization; P. Navratilova, Z.T., and Z.Z.: data acquisition; P. Navratilova, S.P., O.K., H.S., P. Novak, and Z.T.: formal analysis and data management; P. Navratilova, S.P., O.K., and H.S.: writing – original draft; P. Navratilova, H.S., S.P., and Z.Z.: writing; H.S., P. Navratilova, Z.Z., and N.S.: review & editing; and H.S. and N.S.: funding acquisition.

DECLARATION OF INTERESTS

H.S. has the Department of Cell Biology and Genetics at Palacky University in Olomouc as her second affiliation not listed on the title page.

DECLARATION OF GENERATIVE AI AND AI-ASSISTED TECHNOLOGIES IN THE WRITING PROCESS

During the preparation of this work, the authors used ChatGPT4o in order to refine English language and remove possible redundancies, without changing the information content and meaning. After using this tool or service, the authors reviewed and edited the content as needed and take full responsibility for the content of the publication.

STAR★METHODS

Detailed methods are provided in the online version of this paper and include the following:

- KEY RESOURCES TABLE
- EXPERIMENTAL MODEL AND SUBJECT DETAILS
- METHOD DETAILS
  - ATAC-seq
  - ChIP-seq
  - BS-seq
  - HiChIP
  - NET-CAGE
- QUANTIFICATION AND STATISTICAL ANALYSIS
  - ATAC-seq and ChIP-seq data analysis
  - BS-seq data analysis
  - HiChIP data analysis
  - RNA-seq data processing
  - NET-CAGE data analysis
  - Sequence conservation analysis
  - ChromHMM chromatin state analysis
  - RSAT motif analysis
  - GO term analysis

SUPPLEMENTAL INFORMATION

Supplemental information can be found online at https://doi.org/10.1016/j.xgen.2025.101037.

Received: February 16, 2025
Revised: July 4, 2025
Accepted: September 12, 2025
Published: October 10, 2025

REFERENCES

1. Schmitz, R.J., Grotewold, E., and Stam, M. (2022). Cis-regulatory sequences in plants: Their importance, discovery, and future challenges. Plant Cell *34*, 718–741.

2. Lu, Z., Marand, A.P., Ricci, W.A., Ethridge, C.L., Zhang, X., and Schmitz, R.J. (2019). The prevalence, evolution and chromatin signatures of plant regulatory elements. Nat. Plants *5*, 1250–1259.

3. Doležel, J., Čížková, J., Šimková, H., and Bartoš, J. (2018). One Major Challenge of Sequencing Large Plant Genomes Is to Know How Big They Really Are. Int. J. Mol. Sci. *19*, 3554.

4. Navrátilová, P., Toegelová, H., Tulpová, Z., Kuo, Y.T., Stein, N., Doležel, J., Houben, A., Šimková, H., and Mascher, M. (2022). Prospects of telomere-to-telomere assembly in barley: Analysis of sequence gaps in the MorexV3 reference genome. Plant Biotechnol. J. *20*, 1373–1386.

5. Pavlu, S., Nikumbh, S., Kovacik, M., An, T., Lenhard, B., Simkova, H., and Navratilova, P. (2024). Core promoterome of barley embryo. Comput. Struct. Biotechnol. J. *23*, 264–277.

6. Baker, K., Dhillon, T., Colas, I., Cook, N., Milne, I., Milne, L., Bayer, M., and Flavell, A.J. (2015). Chromatin state analysis of the barley epigenome reveals a higher-order structure defined by H3K27me1 and H3K27me3 abundance. Plant J. *84*, 111–124.

7. Li, Z., Wang, M., Lin, K., Xie, Y., Guo, J., Ye, L., Zhuang, Y., Teng, W., Ran, X., Tong, Y., et al. (2019). The bread wheat epigenomic map reveals distinct chromatin architectural and evolutionary features of functional genetic elements. Genome Biol. *20*, 139.

8. Ricci, W.A., Lu, Z., Ji, L., Marand, A.P., Ethridge, C.L., Murphy, N.G., Noshay, J.M., Galli, M., Mejía-Guerra, M.K., Colomé-Tatché, M., et al. (2019). Widespread long-range cis-regulatory elements in the maize genome. Nat. Plants *5*, 1237–1249.

9. Xie, L., Liu, M., Zhao, L., Cao, K., Wang, P., Xu, W., Sung, W.K., Li, X., and Li, G. (2021). RiceENCODE: A comprehensive epigenomic database as a rice Encyclopedia of DNA Elements. Mol. Plant *14*, 1604–1606.

10. Zhao, L., Yang, Y., Chen, J., Lin, X., Zhang, H., Wang, H., Wang, H., Bie, X., Jiang, J., Feng, X., et al. (2023). Dynamic chromatin regulatory programs during embryogenesis of hexaploid wheat. Genome Biol. *24*, 7–29.

11. Lettice, L.A., Heaney, S.J.H., Purdie, L.A., Li, L., de Beer, P., Oostra, B.A., Goode, D., Elgar, G., Hill, R.E., and de Graaff, E. (2003). A long-range Shh enhancer regulates expression in the developing limb and fin and is associated with preaxial polydactyly. Hum. Mol. Genet. *12*, 1725–1735.

12. Crisp, P.A., Marand, A.P., Noshay, J.M., Zhou, P., Lu, Z., Schmitz, R.J., and Springer, N.M. (2020). Stable unmethylated DNA demarcates expressed genes and their cis-regulatory space in plant genomes. Proc. Natl. Acad. Sci. USA *117*, 23991–24000.

13. Weber, B., Zicola, J., Oka, R., and Stam, M. (2016). Plant Enhancers: A Call for Discovery. Trends Plant Sci. *21*, 974–987.

14. Shlyueva, D., Stampfel, G., and Stark, A. (2014). Transcriptional enhancers: from properties to genome-wide predictions. Nat. Rev. Genet. *15*, 272–286.

15. Mozgova, I., Köhler, C., and Hennig, L. (2015). Keeping the gate closed: functions of the polycomb repressive complex PRC2 in development. Plant J. *83*, 121–132.

16. Margueron, R., Trojer, P., and Reinberg, D. (2005). The key to development: interpreting the histone code? Curr. Opin. Genet. Dev. *15*, 163–176.

17. Chen, L., Liu, G., and Zhang, T. (2024). Integrating machine learning and genome editing for crop improvement. aBIOTECH *5*, 262–277.

18. Peleke, F.F., Zumkeller, S.M., Gültas, M., Schmitt, A., and Szymański, J. (2024). Deep learning the cis-regulatory code for gene expression in selected model plants. Nat. Commun. *15*, 3488.

19. Concia, L., Veluchamy, A., Ramirez-Prado, J.S., Martin-Ramirez, A., Huang, Y., Perez, M., Domenichini, S., Rodriguez Granados, N.Y., Kim, S., Blein, T., et al. (2020). Wheat chromatin architecture is organized in genome territories and transcription factories. Genome Biol. *21*, 104.

20. Peng, Y., Xiong, D., Zhao, L., Ouyang, W., Wang, S., Sun, J., Zhang, Q., Guan, P., Xie, L., Li, W., et al. (2019). Chromatin interaction maps reveal genetic regulation for quantitative traits in maize. Nat. Commun. *10*, 2632.

21. Zhao, L., Wang, S., Cao, Z., Ouyang, W., Zhang, Q., Xie, L., Zheng, R., Guo, M., Ma, M., Hu, Z., et al. (2019). Chromatin loops associated with active genes and heterochromatin shape rice genome architecture for transcriptional regulation. Nat. Commun. *10*, 3640.

22. Li, E., Liu, H., Huang, L., Zhang, X., Dong, X., Song, W., Zhao, H., and Lai, J. (2019). Long-range interactions between proximal and distal regulatory regions in maize. Nat. Commun. *10*, 2633.

23. Popay, T.M., and Dixon, J.R. (2022). Coming full circle: On the origin and evolution of the looping model for enhancer-promoter communication. J. Biol. Chem. *298*, 102117.

24. Hsieh, T.-H.S., Cattoglio, C., Slobodyanyuk, E., Hansen, A.S., Darzacq, X., and Tjian, R. (2022). Enhancer-promoter interactions and transcription are largely maintained upon acute loss of CTCF, cohesin, WAPL or YY1. Nat. Genet. *54*, 1919–1932.

25. Rao, S.S.P., Huang, S.C., Glenn St Hilaire, B., Engreitz, J.M., Perez, E.M., Kieffer-Kwon, K.R., Sanborn, A.L., Johnstone, S.E., Bascom, G.D., Bochkov, I.D., et al. (2017). Cohesin Loss Eliminates All Loop Domains. Cell *171*, 305–320.e24.

26. Kraft, K., Yost, K.E., Murphy, S.E., Magg, A., Long, Y., Corces, M.R., Granja, J.M., Wittler, L., Mundlos, S., Cech, T.R., et al. (2022). Polycomb-mediated genome architecture enables long-range spreading of H3K27 methylation. Proc. Natl. Acad. Sci. USA *119*, e2201883119.

27. Dekker, J., Marti-Renom, M.A., and Mirny, L.A. (2013). Exploring the three-dimensional organization of genomes: interpreting chromatin interaction data. Nat. Rev. Genet. *14*, 390–403.

28. Mumbach, M.R., Rubin, A.J., Flynn, R.A., Dai, C., Khavari, P.A., Greenleaf, W.J., and Chang, H.Y. (2016). HiChIP: efficient and sensitive analysis of protein-directed genome architecture. Nat. Methods *13*, 919–922.

29. Mifsud, B., Tavares-Cadete, F., Young, A.N., Sugar, R., Schoenfelder, S., Ferreira, L., Wingett, S.W., Andrews, S., Grey, W., Ewels, P.A., et al. (2015). Mapping long-range promoter contacts in human cells with high-resolution capture Hi-C. Nat. Genet. *47*, 598–606.

30. Šimková, H., Câmara, A.S., and Mascher, M. (2024). Hi-C techniques: from genome assemblies to transcription regulation. J. Exp. Bot. *75*, 5357–5365.

31. de Lara, J.C.-F., Arzate-Mejía, R.G., and Recillas-Targa, F. (2019). Enhancer RNAs: Insights Into Their Biological Role. Epigenet. Insights *12*, 2516865719846093.

32. Core, L.J., Martins, A.L., Danko, C.G., Waters, C.T., Siepel, A., and Lis, J. T. (2014). Analysis of nascent RNA identifies a unified architecture of initiation regions at mammalian promoters and enhancers. Nat. Genet. *46*, 1311–1320.

33. McDonald, B.R., Picard, C.L., Brabb, I.M., Savenkova, M.I., Schmitz, R. J., Jacobsen, S.E., and Duttke, S.H. (2024). Enhancers associated with unstable RNAs are rare in plants. Nat. Plants *10*, 1246–1257.

34. Xie, Y., Chen, Y., Li, Z., Zhu, J., Liu, M., Zhang, Y., and Dong, Z. (2022). Enhancer transcription detected in the nascent transcriptomic landscape of bread wheat. Genome Biol. *23*, 109.

35. Liu, C., Zhu, X., Zhang, J., Shen, M., Chen, K., Fu, X., Ma, L., Liu, X., Zhou, C., Zhou, D.X., and Wang, G. (2022). eQTLs play critical roles in regulating gene expression and identifying key regulators in rice. Plant Biotechnol. J. *20*, 2357–2371.

36. Leprince, O., Pellizzaro, A., Berriri, S., and Buitink, J. (2017). Late seed maturation: drying without dying. J. Exp. Bot. *68*, 827–841.

37. Artur, M.A.S., Zhao, T., Ligterink, W., Schranz, E., and Hilhorst, H.W.M. (2019). Dissecting the Genomic Diversification of Late Embryogenesis Abundant (LEA) Protein Gene Families in Plants. Genome Biol. Evol. *11*, 459–471.

38. Jayakodi, M., Lu, Q., Pidon, H., Rabanus-Wallace, M.T., Bayer, M., Lux, T., Guo, Y., Jaegle, B., Badea, A., Bekele, W., et al. (2024). Structural

variation in the pangenome of wild and domesticated barley. Nature *636*, 654–662.

39. Liu, Y., Liu, P., Gao, L., Li, Y., Ren, X., Jia, J., Wang, L., Zheng, X., Tong, Y., Pei, H., and Lu, Z. (2024). Epigenomic identification of vernalization cis-regulatory elements in winter wheat. Genome Biol. *25*, 200.
40. Liu, Y., Xu, X., He, C., Jin, L., Zhou, Z., Gao, J., Guo, M., Wang, X., Chen, C., Ayaad, M.H., et al. (2024). Chromatin loops gather targets of upstream regulators together for efficient gene transcription regulation during vernalization in wheat. Genome Biol. *25*, 306.
41. Yan, L., Fu, D., Li, C., Blechl, A., Tranquilli, G., Bonafede, M., Sanchez, A., Valarik, M., Yasuda, S., and Dubcovsky, J. (2006). The wheat and barley vernalization gene VRN3 is an orthologue of FT. Proc. Natl. Acad. Sci. USA *103*, 19581–19586.
42. Wicker, T., Schulman, A.H., Tanskanen, J., Spannagl, M., Twardziok, S., Mascher, M., Springer, N.M., Li, Q., Waugh, R., Li, C., et al. (2017). The repetitive landscape of the 5100 Mbp barley genome. Mob. DNA *8*, 22.
43. Gasparis, S., Przyborowski, M., and Nadolska-Orczyk, A. (2021). Genome-Wide Identification of Barley Long Noncoding RNAs and Analysis of Their Regulatory Interactions during Shoot and Grain Development. Int. J. Mol. Sci. *22*, 5087.
44. Ernst, J., and Kellis, M. (2012). ChromHMM: automating chromatin-state discovery and characterization. Nat. Methods *9*, 215–216.
45. Conway, J.R., Lex, A., and Gehlenborg, N. (2017). UpSetR: an R package for the visualization of intersecting sets and their properties. Bioinformatics *33*, 2938–2940.
46. Hardison, R.C. (2000). Conserved noncoding sequences are reliable guides to regulatory elements. Trends Genet. *16*, 369–372.
47. Miller, W., Rosenbloom, K., Hardison, R.C., Hou, M., Taylor, J., Raney, B., Burhans, R., King, D.C., Baertsch, R., Blankenberg, D., et al. (2007). 28-way vertebrate alignment and conservation track in the UCSC Genome Browser. Genome Res. *17*, 1797–1808.
48. Castro-Mondragon, J.A., Rioualen, C., Contreras-Moreira, B., and van Helden, J. (2016). RSAT::Plants: Motif discovery in ChIP-seq peaks of plant genomes. Methods Mol. Biol. *1482*, 297–322.
49. Ding, J., Karim, H., Li, Y., Harwood, W., Guzmán, C., Lin, N., Xu, Q., Zhang, Y., Tang, H., Jiang, Y., et al. (2021). Re-examination of the Gene Family in Barley (L.) Indicates a Role in the Regulation of Starch Synthesis. Front. Plant Sci. *12*, 791584.
50. Wani, S.H., and et al.. (2023). Mechanisms of Abiotic Stress Responses and Tolerance in Plants: Physiological, Biochemical and Molecular Interventions, Volume II. Front. Plant Sci. *14*, 1272255.
51. Hirabayashi, S., Bhagat, S., Matsuki, Y., Takegami, Y., Uehata, T., Kanemaru, A., Itoh, M., Shirakawa, K., Takaori-Kondo, A., Takeuchi, O., et al. (2019). NET-CAGE characterizes the dynamics and topology of human transcribed cis-regulatory elements. Nat. Genet. *51*, 1369–1379.
52. Bhattacharyya, S., Chandra, V., Vijayanand, P., and Ay, F. (2019). Identification of significant chromatin contacts from HiChIP data by FitHiChIP. Nat. Commun. *10*, 4221.
53. Mascher, M., Gundlach, H., Himmelbach, A., Beier, S., Twardziok, S.O., Wicker, T., Radchuk, V., Dockter, C., Hedley, P.E., Russell, J., et al. (2017). A chromosome conformation capture ordered sequence of the barley genome. Nature *544*, 427–433.
54. Milne, L., Bayer, M., Rapazote-Flores, P., Mayer, C.D., Waugh, R., and Simpson, C.G. (2021). EORNA, a barley gene and transcript abundance database. Sci. Data *8*, 90.
55. Kovacik, M., Nowicka, A., Zwyrtková, J., Strejčková, B., Vardanega, I., Esteban, E., Pasha, A., Kaduchová, K., Krautsova, M., Červenková, M., et al. (2024). The transcriptome landscape of developing barley seeds. Plant Cell *36*, 2512–2530.
56. Chesmore, K.N., Bartlett, J., Cheng, C., and Williams, S.M. (2016). Complex Patterns of Association between Pleiotropy and Transcription Factor Evolution. Genome Biol. Evol. *8*, 3159–3170.
57. Kikuta, H., Fredman, D., Rinkwitz, S., Lenhard, B., and Becker, T.S. (2007). Retroviral enhancer detection insertions in zebrafish combined with comparative genomics reveal genomic regulatory blocks - a fundamental feature of vertebrate genomes. Genome Biol. *8*, S4.
58. Harrison, P.W., Amode, M.R., Austine-Orimoloye, O., Azov, A.G., Barba, M., Barnes, I., Becker, A., Bennett, R., Berry, A., Bhai, J., et al. (2024). Ensembl 2024. Nucleic Acids Res. *52*, D891–D899.
59. Waese, J., Fan, J., Pasha, A., Yu, H., Fucile, G., Shi, R., Cumming, M., Kelley, L.A., Sternberg, M.J., Krishnakumar, V., et al. (2017). ePlant: Visualizing and Exploring Multiple Levels of Data for Hypothesis Generation in Plant Biology. Plant Cell *29*, 1806–1821.
60. Tian, F., Yang, D.-C., Meng, Y.-Q., Jin, J., and Gao, G. (2020). PlantRegMap: charting functional regulatory maps in plants. Nucleic Acids Res. *48*, D1104–D1113.
61. Mascher, M., Wicker, T., Jenkins, J., Plott, C., Lux, T., Koh, C.S., Ens, J., Gundlach, H., Boston, L.B., Tulpová, Z., et al. (2021). Long-read sequence assembly: a technical evaluation in barley. Plant Cell *33*, 1888–1906.
62. Cheng, S., Miao, B., Li, T., Zhao, G., and Zhang, B. (2024). Review and Evaluate the Bioinformatics Analysis Strategies of ATAC-seq and CUT&Tag Data. Genom. Proteom. Bioinform. *22*, qzae054.
63. Singh, D., and Yi, S.V. (2021). Enhancer Pleiotropy, Gene Expression, and the Architecture of Human Enhancer-Gene Interactions. Mol. Biol. Evol. *38*, 3898–3909.
64. Thormann, V., Rothkegel, M.C., Schöpflin, R., Glaser, L.V., Djuric, P., Li, N., Chung, H.R., Schwahn, K., Vingron, M., and Meijsing, S.H. (2018). Genomic dissection of enhancers uncovers principles of combinatorial regulation and cell type-specific wiring of enhancer-promoter contacts. Nucleic Acids Res. *46*, 3258.
65. Yao, L., Liang, J., Ozer, A., Leung, A.K.Y., Lis, J.T., and Yu, H. (2022). A comparison of experimental assays and analytical methods for genome-wide identification of active enhancers. Nat. Biotechnol. *40*, 1056–1065.
66. Silver, B.D., Willett, C.G., Maher, K.A., Wang, D., and Deal, R.B. (2024). Differences in transcription initiation directionality underlie distinctions between plants and animals in chromatin modification patterns at genes and cis-regulatory elements. G3 *14*, jkae016.
67. Borsari, B., Villegas-Mirón, P., Pérez-Lluch, S., Turpin, I., Laayouni, H., Segarra-Casas, A., Bertranpetit, J., Guigó, R., and Acosta, S. (2021). Enhancers with tissue-specific activity are enriched in intronic regions. Genome Res. *31*, 1325–1336.
68. Macrae, T.A., Fothergill-Robinson, J., and Ramalho-Santos, M. (2023). Regulation, functions and transmission of bivalent chromatin during mammalian development. Nat. Rev. Mol. Cell Biol. *24*, 6–26.
69. Zhu, D., Wen, Y., Yao, W., Zheng, H., Zhou, S., Zhang, Q., Qu, L.J., Chen, X., and Wu, Z. (2023). Distinct chromatin signatures in the Arabidopsis male gametophyte. Nat. Genet. *55*, 706–720.
70. Gao, Z., Li, Y., Ou, Y., Yin, M., Chen, T., Zeng, X., Li, R., and He, Y. (2023). A pair of readers of bivalent chromatin mediate formation of Polycomb-based ‘memory of cold’ in plants. Mol. Cell *83*, 1109–1124.e4.
71. Seneviratne, J.A., Ho, W.W.H., Glancy, E., and Eckersley-Maslin, M.A. (2024). A low-input high resolution sequential chromatin immunoprecipitation method captures genome-wide dynamics of bivalent chromatin. Epigenetics Chromatin *17*, 3.
72. Zhu, I., Song, W., Ovcharenko, I., and Landsman, D. (2021). A model of active transcription hubs that unifies the roles of active promoters and enhancers. Nucleic Acids Res. *49*, 4493–4505.
73. Yuan, J., Sun, H., Wang, Y., Li, L., Chen, S., Jiao, W., Jia, G., Wang, L., Mao, J., Ni, Z., et al. (2022). Open chromatin interaction maps reveal functional regulatory elements and chromatin architecture variations during wheat evolution. Genome Biol. *23*, 34.
74. Carelli, F.N., Liechti, A., Halbert, J., Warnefors, M., and Kaessmann, H. (2018). Repurposing of promoters and enhancers during mammalian evolution. Nat. Commun. *9*, 4066.

75. Andersson, R., and Sandelin, A. (2020). Determinants of enhancer and promoter activities of regulatory elements. Nat. Rev. Genet. *21*, 71–87.

76. Jores, T., Tonnies, J., Mueth, N.A., Romanowski, A., Fields, S., Cuperus, J.T., and Queitsch, C. (2024). Plant enhancers exhibit both cooperative and additive interactions among their functional elements. Plant Cell *36*, 2570–2586.

77. Zhou, W., Shi, H., Wang, Z., Huang, Y., Ni, L., Chen, X., Liu, Y., Li, H., Li, C., and Liu, Y. (2024). Identification of Highly Repetitive Enhancers with Long-range Regulation Potential in Barley via STARR-seq. Genom. Proteom. Bioinform. *22*, qzae012.

78. Butt, A.H., Alkhalifah, T., Alturise, F., and Khan, Y.D. (2022). A machine learning technique for identifying DNA enhancer regions utilizing CIS-regulatory element patterns. Sci. Rep. *12*, 15183.

79. Liu, G., Chen, L., Wu, Y., Han, Y., Bao, Y., and Zhang, T. (2025). PDLLMs: A group of tailored DNA large language models for analyzing plant genomes. Mol. Plant *18*, 175–178. https://doi.org/10.1016/j.molp.2024.12.006.

80. Mendoza-Revilla, J., Trop, E., Gonzalez, L., Roller, M., Dalla-Torre, H., de Almeida, B.P., Richard, G., Caton, J., Lopez Carranza, N., Skwark, M., et al. (2024). A foundational large language model for edible plant genomes. Commun. Biol. *7*, 835.

81. Sebastian, A., and Contreras-Moreira, B. (2014). footprintDB: a database of transcription factors with annotated cis elements and binding interfaces. Bioinformatics. 30258265.

82. Langmead, B., and Salzberg, S.L. (2012). Fast gapped-read alignment with Bowtie 2. Nat. Methods *9*, 357–359.

83. Ramírez, F., Ryan, D.P., Grüning, B., Bhardwaj, V., Kilpert, F., Richter, A. S., Heyne, S., Dündar, F., and Manke, T. (2016). deepTools2: a next generation web server for deep-sequencing data analysis. Nucleic Acids Res. *44*, W160–W165.

84. Zhang, Y., Liu, T., Meyer, C.A., Eeckhoute, J., Johnson, D.S., Bernstein, B.E., Nusbaum, C., Myers, R.M., Brown, M., Li, W., and Liu, X.S. (2008). Model-based analysis of ChIP-Seq (MACS). Genome Biol. *9*, R137.

85. Krueger, F., and Andrews, S.R. (2011). Bismark: a flexible aligner and methylation caller for Bisulfite-Seq applications. Bioinformatics *27*, 1571–1572.

86. Li, G., Ruan, X., Auerbach, R.K., Sandhu, K.S., Zheng, M., Wang, P., Poh, H.M., Goh, Y., Lim, J., Zhang, J., et al. (2012). Extensive promoter-centered chromatin interactions provide a topological basis for transcription regulation. Cell *148*, 84–98.

87. Dobin, A., Davis, C.A., Schlesinger, F., Drenkow, J., Zaleski, C., Jha, S., Batut, P., Chaisson, M., and Gingeras, T.R. (2013). STAR: ultrafast universal RNA-seq aligner. Bioinformatics *29*, 15–21.

88. Li, B., and Dewey, C.N. (2011). RSEM: accurate transcript quantification from RNA-Seq data with or without a reference genome. BMC Bioinf. *12*, 323.

89. Quinlan, A.R., and Hall, I.M. (2010). BEDTools: a flexible suite of utilities for comparing genomic features. Bioinformatics *26*, 841–842.

90. Shumate, A., and Salzberg, S.L. (2021). Liftoff: accurate mapping of gene annotations. Bioinformatics *37*, 1639–1643.

91. Love, M.I., Huber, W., and Anders, S. (2014). Moderated estimation of fold change and dispersion for RNA-seq data with DESeq2. Genome Biol. *15*, 550.

92. Li, H. (2013). Aligning sequence reads, clone sequences and assembly contigs with BWA-MEM. Preprint at arXiv. https://doi.org/10.48550/arXiv.1303.3997.

93. Kim, D., Paggi, J.M., Park, C., Bennett, C., and Salzberg, S.L. (2019). Graph-based genome alignment and genotyping with HISAT2 and HISAT-genotype. Nat. Biotechnol. *37*, 907–915.

94. Haberle, V., Forrest, A.R.R., Hayashizaki, Y., Carninci, P., and Lenhard, B. (2015). CAGEr: precise TSS data retrieval and high-resolution promoterome mining for integrative analyses. Nucleic Acids Res. *43*, e51.

95. Yu, G., Wang, L.-G., and He, Q.-Y. (2015). ChIPseeker: an R/Bioconductor package for ChIP peak annotation, comparison and visualization. Bioinformatics *31*, 2382–2383.

96. Gel, B., Díez-Villanueva, A., Serra, E., Buschbeck, M., Peinado, M.A., and Malinverni, R. (2016). regioneR: an R/Bioconductor package for the association analysis of genomic regions based on permutation tests. Bioinformatics *32*, 289–291.

97. Plessy, C., Mansfield, M.J., Bliznina, A., Masunaga, A., West, C., Tan, Y., Liu, A.W., Grašič, J., Del Río Pisula, M.S., Sánchez-Serna, G., et al. (2024). Extreme genome scrambling in marine planktonic cryptic species. Genome Res. *34*, 426–440.

98. Hou, M. & Riemer, C. (2008). TOAST and ROAST. https://www.bx.psu.edu/∼cathy/toast-roast.tmp/README.toast-roast.html.

99. Hubisz, M.J., Pollard, K.S., and Siepel, A. (2011). PHAST and RPHAST: phylogenetic analysis with space/time models. Brief. Bioinform. *12*, 41–51.

100. Santana-Garcia, W., Castro-Mondragon, J.A., Padilla-Gálvez, M., Nguyen, N.T.T., Elizondo-Salas, A., Ksouri, N., Gerbes, F., Thieffry, D., Vincens, P., Contreras-Moreira, B., et al. (2022). RSAT 2022: regulatory sequence analysis tools. Nucleic Acids Res. *50*, W670–W676.

101. Wimalanathan, K., and Lawrence-Dill, C.J. (2021). Gene Ontology Meta Annotator for Plants (GOMAP). Plant Methods *17*, 54.

102. Wu, T., Hu, E., Xu, S., Chen, M., Guo, P., Dai, Z., Feng, T., Zhou, L., Tang, W., Zhan, L., et al. (2021). clusterProfiler 4.0: A universal enrichment tool for interpreting omics data. Innovation *2*, 100141.

103. Supek, F., Bošnjak, M., Škunca, N., and Šmuc, T. (2011). REVIGO summarizes and visualizes long lists of gene ontology terms. PLoS One *6*, e21800.

104. Kovacik, M., Nowicka, A., and Pecinka, A. (2020). Isolation of High Purity Tissues from Developing Barley Seeds. J. Vis. Exp. *26*. https://doi.org/10.3791/61681.

105. Doležel, J., Sgorbati, S., and Lucretti, S. (1992). Comparison of three DNA fluorochromes for flow cytometric estimation of nuclear DNA content in plants. Physiol. Plant. *85*, 625–631.

106. Ramirez-Prado, J.S., Latrasse, D., and Benhamed, M. (2021). Histone Modification ChIP-seq on Plantlets. Bio. Protoc. *11*, e4211.

107. Reske, J.J., Wilson, M.R., and Chandler, R.L. (2020). ATAC-seq normalization method can significantly affect differential accessibility analysis and interpretation. Epigenetics Chromatin *13*, 22.

108. Servant, N., Varoquaux, N., Lajoie, B.R., Viara, E., Chen, C.J., Vert, J.P., Heard, E., Dekker, J., and Barillot, E. (2015). HiC-Pro: an optimized and flexible pipeline for Hi-C data processing. Genome Biol. *16*, 259.

109. Ling, H.-Q., Ma, B., Shi, X., Liu, H., Dong, L., Sun, H., Cao, Y., Gao, Q., Zheng, S., Li, Y., et al. (2018). Genome sequence of the progenitor of wheat A subgenome Triticum urartu. Nature *557*, 424–428.

110. Rabanus-Wallace, M.T., Hackauf, B., Mascher, M., Lux, T., Wicker, T., Gundlach, H., Baez, M., Houben, A., Mayer, K.F.X., Guo, L., et al. (2021). Chromosome-scale genome assembly provides insights into rye biology, evolution and agronomic potential. Nat. Genet. *53*, 564–573.

111. International Brachypodium Initiative (2010). Genome sequencing and analysis of the model grass Brachypodium distachyon. Nature *463*, 763–768.

112. Kiełbasa, S.M., Wan, R., Sato, K., Horton, P., and Frith, M.C. (2011). Adaptive seeds tame genomic sequence comparison. Genome Res. *21*, 487–493.

113. International Wheat Genome Sequencing Consortium IWGSC (2018). Shifting the limits in wheat research and breeding using a fully annotated reference genome. Science *361*, eaar7191.

114. Sebastian, A., and Contreras-Moreira, B. (2014). footprintDB: a database of transcription factors with annotated cis elements and binding interfaces. Bioinformatics *30*, 258–265.

STAR★METHODS

KEY RESOURCES TABLE

REAGENT or RESOURCE	SOURCE	IDENTIFIER
Antibodies		
Anti-Histone H3 (tri methyl K4) antibody	Abcam	Cat# ab213224; RRID: AB_2923013
Anti-Histone H3 (tri methyl K27) antibody	Abcam	Cat# ab6002; RRID: AB_305237
Anti-Histone H3 (acetyl K9) antibody	Abcam	Cat# ab4441; RRID: AB_2118292
Anti-trimethyl-Histone H3 (Lys4) antibody, Clone MC315	Millipore	Cat# 04-745; RRID: AB_1163444
H3K27me3 antibody	Diagenode	Cat# C15410195; RRID: AB_2753161
Chemicals, peptides, and recombinant proteins		
Pierce^{TM} 16% Formaldehyde (w/v), Methanol-free	Thermo Fisher Scientific	Cat# 28906
Formaldehyde solution 37 wt. %, 10–15% methanol	Sigma-Aldrich	Cat# 252549
α-Amanitin	Sigma-Aldrich	Cat# A2263
cOmplete^{TM} Mini, EDTA-free Protease Inhibitor Cocktail	Roche	Cat# 11836170001
Halt^{TM} Protease Inhibitor Coctail	Thermo Fisher Scientific	Cat# 78429
RNaseOUT^{TM} Recombinant Ribonuclease Inhibitor	Invitrogen	Cat# 10777019
NEBNext High-Fidelity 2x PCR Master Mix	New England Biolabs	Cat# M0541S
VAHTS DNA Clean Beads	Vazyme	Cat# N411-01
Dynabeads^{TM} Protein A	Invitrogen	Cat# 10001D
Critical commercial assays		
ATAC-seq kit	Active Motif	Cat# 53150
Tagment DNA TDE1 Enzyme	Illumina	Cat# 20034197
MinElute PCR Purification kit	Qiagen	Cat# 28004
iPure kit v2	Diagenode	Cat# C03010015
NEBNext Ultra^{TM} II DNA Library Prep kit for Illumina	New England Biolabs	Cat# E7645S
NucleoSpin Plant II kit	Macherey-Nagel	Cat# 740770
Zymo-Seq WGBS Library kit	Zymo Research	Cat# D5465
Arima HiC+ kit	Arima Genomics	Cat# A101020
Accel-NGS 2S Plus DNA Library kit	Swift Biosciences	Cat# 210024
PureLink^{TM} miRNA Isolation kit	Invitrogen	Cat# K157001
Qubit^{TM} 1X dsDNA High Sensitivity Assay kit	Invitrogen	Cat# Q33230
Deposited data		
Raw data	This paper	SRA: PRJNA1177611
Processed data	This paper	https://olomouc.ueb.cas.cz/en/resources/barleyepibase
ChIP-seq data for three embryonal stages	Pavlu et al.[5]	GEO: GSE227218
BS-seq data for leaf	Wicker et al.[42]	SRA: PRJEB14349
RNA-seq data for 16 tissues	Mascher et al.[53]	SRA: PRJEB14349
RNA-seq data for 8DAP and 24DAP embryo	Kovacik et al.[55]	GEO: GSE233316
lncRNA	Gasparis et al.[43]	https://www.tobaccodb.org/plncdb/Download
footprintDB	Sebastian and Contreras-Moreira[81]	https://footprintdb.eead.csic.es/

(Continued on next page)

Continued		
REAGENT or RESOURCE	SOURCE	IDENTIFIER
MorexV3 genome	Mascher et al.[61]	https://doi.ipk-gatersleben.de/DOI/b2f47dfb-47ff-4114-89ae-bad8dcc515a1/7eb2707b-d447-425c-be7a-fe3f1fae67cb/2
Software and algorithms		
Original code	This paper	https://github.com/MorexV3CAGE/Barley_distal_regulome/tree/main/scripts
Trim Galore	https://doi.org/10.5281/zenodo.5127898	https://github.com/FelixKrueger/TrimGalore
Bowtie2	Langmead and Salzberg[82]	https://github.com/BenLangmead/bowtie2
deeptools	Ramírez et al.[83]	https://github.com/deeptools/deepTools
MACS2	Zhang et al.[83]	https://github.com/macs3-project/MACS
Bismark	Krueger and Andrews[84]	https://github.com/FelixKrueger/Bismark
HiC-PRO	Servant et al.[85]	https://github.com/nservant/HiC-Pro
FitHiChIP	Bhattacharyya et al.[52]	https://github.com/ay-lab/FitHiChIP
R	The R Foundation for Statistical Computing	https://www.r-project.org
GenomicInteractions	Li et al.[86]	https://github.com/ComputationalRegulatoryGenomicsICL/GenomicInteractions
STAR	Dobin et al.[87]	https://github.com/alexdobin/STAR
RSEM	Li and Dewey[88]	https://github.com/deweylab/RSEM
bedtools	Quinlan and Hall[89]	https://github.com/arq5x/bedtools2
liftoff	Shumate and Salzberg[90]	https://github.com/agshumate/Liftoff
DESeq2	Love et al.[91]	https://github.com/thelovelab/DESeq2
BWA-MEM	Li[92]	https://github.com/lh3/bwa
HiSat2	Kim et al.[93]	https://github.com/DaehwanKimLab/hisat2
CAGEr	Haberle et al.[94]	https://github.com/charles-plessy/CAGEr
ChIPseeker	Yu et al.[95]	https://github.com/YuLab-SMU/ChIPseeker
regioneR	Gel et al.[96]	https://github.com/bernatgel/regioneR
pairgenomealign	Plessy et al.[97]	https://nf-co.re/pairgenomealign/dev/
ROAST	Hou and Riemer[98]	https://anaconda.org/bioconda/multiz
PHAST	Hubisz et al.[99]	http://compgen.cshl.edu/phast/
ChromHMM	Ernst and Kellis[44]	https://github.com/jernst98/ChromHMM
RSAT	Santana-Garcia et al.[100]	https://github.com/rsa-tools/rsat-code
GOMAP	Wimalanathan and Lawrence-Dill[101]	https://github.com/Dill-PICL/GOMAP
clusterProfiler	Wu et al.[102]	https://github.com/YuLab-SMU/clusterProfiler
REVIGO	Supek et al.[103]	http://revigo.irb.hr/

EXPERIMENTAL MODEL AND SUBJECT DETAILS

Barley cv. Morex was grown in growth chambers (Weiss Gallenkamp, walk-in chambers) at 16/8 h light cycle, 16°C day/12°C night temperature. To obtain 4DAG seedlings, seeds were germinated on wet tissue paper at 20°C for four days before harvesting and removing remnants of seed coat and endosperm. The 8DAP and 24DAP embryos were staged according to their time of fertilization, size and phenotype and dissected as described previously.[104] For collecting leaf samples, plants were grown in growth chambers at 16/8 h light cycle, 20°C day/16°C night temperature for two weeks.

METHOD DETAILS

ATAC-seq

Barley embryos were fixed for 8 min in 1% Pierce methanol-free formaldehyde (Thermo Fisher Scientific, 28906) in PBS under vacuum. Fixation was stopped by 5-min incubation in 0.125 M glycine in PBS followed by thorough PBS washes. The tissues were pulverized by mortar and pestle in liquid nitrogen and nuclei were extracted in lysis buffer LB01[105] supplemented with cOmplete Mini,

EDTA-free Protease Inhibitor Cocktail (Roche, 11836170001). Twenty-five thousand G1-phase nuclei per sample, counterstained with DAPI, were purified by FACSAria II SORP flow cytometer and sorter (BD Bioscience) and processed using ATAC-seq kit (Active Motif, 53150) with a decrosslinking step included before DNA purification. Tagmented and amplified libraries were sequenced (2 × 150 bp reads) on the NovaSeq 6000 platform (Illumina) at the IEB Olomouc.

Fresh leaf samples from two-week-old seedlings were finely chopped using a razor blade in Nuclei Isolation Buffer (0.25 M sucrose, 10 mM Tris-HCl pH 8.0, 10 mM MgCl2, 1% Triton X-100, 5 mM β-Mercaptoethanol) supplemented with 1x Halt Protease Inhibitor Cocktail (Thermo Fisher Scientific, 78429). Seventy-five thousand nuclei per sample were purified by flow cytometry and processed using Tagment DNA TDE1 Enzyme (Illumina, 20034197). Tagmentation products were purified using the MinElute PCR Purification Kit (Qiagen, 28004), amplified using the NEBNext High-Fidelity 2x PCR Master Mix (NEB, M0541), and further purified using the VAHTS DNA Clean Beads (Vazyme, N411). The final libraries were sequenced on the NovaSeq 6000 platform at the IPK Gatersleben.

ChIP-seq

The ChIP experiment for leaf was performed according to a previously described protocol[106] with minor modifications. Fresh leaves (3 g) from two-week-old seedlings were fixed under vacuum in 1% formaldehyde (Sigma-Aldrich, 252549) for 15 min. Fixation was stopped by a 5-min incubation in 0.125 M glycine. The tissues were pulverized using a mortar and pestle in liquid nitrogen and nuclei were extracted. Nuclei samples were resuspended in Nuclei Lysis Buffer containing 0.1% SDS in a 1-mL sonication tube (Covaris). Chromatin was sonicated for 250 s in a Covaris S220 instrument (Covaris) with settings of peak power 175 W, cycles/burst 200, and duty factor 20%. The sonicated chromatin was cleaned by centrifugation, and the supernatant was diluted four times using ChIP Dilution Buffer. The diluted chromatin (800 μL) was incubated with the respective antibodies (anti-H3K4me3, Abcam, ab213224; anti-H3K27me3, Abcam, ab6002; anti-H3K9ac, Abcam, ab4441) at 4°C for 16 h. Washed Dynabeads Protein A (Invitrogen, 10001D), 40 μL per sample, were added to the antibody-bound chromatin and incubated at 4°C for 2 h. The collected beads were washed twice sequentially in Low Salt Buffer, High Salt Buffer, and TE Buffer. The bead-bound chromatin was purified using the iPure kit v2 (Diagenode, C03010015) according to the manufacturer's instructions. Purified DNA was quantified using the Qubit 1X dsDNA High Sensitivity Assay kit (Invitrogen, Q33230). ChIP-seq libraries were prepared using the NEBNext Ultra II DNA Library Prep Kit for Illumina (New England Biolabs, E7645S) and sequenced (2 × 150 bp reads) on the NovaSeq 6000 platform at the IPK Gatersleben.

BS-seq

DNA for preparation of whole-genome BS-seq (WGBS) libraries was isolated from three frozen 24DAP embryos using NucleoSpin Plant II kit (Macherey-Nagel, 740770) in two biological replicates. DNA was quantified using the Qubit 1X dsDNA High Sensitivity Assay kit. To assess the conversion efficiency, 100 ng input DNA was spiked with 1 ng of *E. coli* DNA. Both bisulfite conversion and WGBS library preparation were done using the Zymo-Seq WGBS Library kit (Zymo Research, D5465) following the manufacturer's instructions, modified by shortening the tagmentation step from 15 to 10 min. Paired-end 2 × 150bp reads were generated on the NovaSeq 6000 platform at the IEB Olomouc.

HiChIP

The 24DAP embryos were fixed under vacuum in 2% formaldehyde in PBS for 15 min. Fixation was stopped by 5-min incubation in 0.125 M glycine in PBS and subsequent PBS washes. The tissues were pulverized by mortar and pestle in liquid nitrogen and nuclei were released in lysis buffer LB01[105] supplemented with cOmplete Mini, EDTA-free Protease Inhibitor Cocktail. Five million G1-phase nuclei were purified by FACSAria II SORP into LB01 buffer supplemented with cOmplete Mini, EDTA-free Protease Inhibitor Cocktail. Digestion and proximal ligation were done using the Arima HiC+ kit (Arima Genomics, A101020). HiChIP was performed according to the Arima HiChIP protocol A160168 v00, followed by library preparation using the Accel-NGS 2S Plus DNA Library kit (Swift Biosciences, 210024) and protocol A160169 v00. For histone-modification enrichment, we used anti-H3K4me3 antibody 04–745 (Millipore) and anti-H3K27me3 antibody C15410195 (Diagenode), respectively. The HiChIP libraries were sequenced at 2 × 150 bp reads on the NovaSeq 6000 platform at the IEB Olomouc.

NET-CAGE

Two replicates of 4DAG embryos were collected in liquid nitrogen and grinded by mortar and pestle for nuclei isolation. To avoid transcriptional run-on, we added α-amanitin (Sigma-Aldrich, A2263), a potent inhibitor of transcription, and RNaseOUT Recombinant Ribonuclease Inhibitor (Invitrogen, 10777019) at each experimental step. We purified nuclei from 4DAG embryos by flow cytometry, followed by isolation of small RNAs using the PureLink miRNA Isolation kit (Invitrogen, K157001). The cap-trapping, library preparation and sequencing were performed by K.K. DNAFORM company. The libraries were sequenced on the NovaSeq 6000 platform at 2 × 150 bp.

QUANTIFICATION AND STATISTICAL ANALYSIS

ATAC-seq and ChIP-seq data analysis

All sequencing datasets were trimmed with Trim Galore (v0.6.4). The ATAC-seq data was analyzed according to.[107] The ATAC-seq and ChIP-seq reads were mapped (bowtie2 v2.4.2[82]) and normalized coverages were calculated using deeptools[83] bamCoverage

and bamCompare (v3.5.1), respectively. MACS2 (v2.2.7.1) program[84] with parameters –broad –nomodel was used to identify the peaks.

BS-seq data analysis

The BS-seq reads were trimmed using Trim Galore (v0.6.4) with –trim1 –paired options. Trimmed reads were aligned by the Bismark program (v0.19.0)[85] with the default settings, deduplicated and data for each methylation context were generated. Subsequently, we defined intergenic unmethylated regions (UMRs) using the approach described by.[12] We only considered cytosines with minimal coverage of 5 and defined the UMRs by setting a 1% methylation threshold in every context throughout 300-bp intergenic fragments. Using publicly available BS-seq data from leaf tissue,[42] we defined leaf UMRs using the same criteria.

HiChIP data analysis

Each replicate contained approximately 500 million reads, which were trimmed and processed by the HiC-Pro (v3.0.0) pipeline.[108] Replicates were merged after the deduplication step for further valid-pair processing. The resulting intra-chromosomal contact maps were further processed by FitHiChIP (Release 6.0)[52] for significant loop calling using previously generated ChIP-seq peaks[5] as a reference. In this process, 2-Mb distance was set as a maximum distance, nearby interactions were merged, peak-to-all interactions were considered and the FDR value limit was set to 0.05 or 0.1. GenomicInteractions package[86] was used for the genomic feature annotation of the loop anchors.

RNA-seq data processing

Trimmed RNA-seq reads from 8DAP, 24DAP, and 4DAG embryos, as well as leaf tissue, obtained from published studies,[53,55] were mapped to the MorexV3 genome[61] using STAR (version 2.7.6a),[87] followed by transcript quantification by RSEM (v1.3.3)[88] software package. Genomic coverages were calculated using deeptools bamCoverage (v3.5.1)[83] for visualization. To account for potentially unannotated genes, we have merged all the mapped RNA-seq data into a single bed file using bedtools bamtobed[89] and selected for intergenic regions that had TPM >1. The lncRNA coordinates[43] were converted by liftoff[90] software from MorexV1 to MorexV3 genome assembly. The expression data were analyzed by DESeq2[91] for differential expression. For visualization, the data were normalized using the vst function.

NET-CAGE data analysis

Sequencing reads were filtered to remove rRNA reads and mapped using BWA[92] with MAPQ<=20 and HiSat2[93] on MorexV3 genome and transcriptome.

Data from both replicas were processed and merged in the CAGEr pipeline (v2.6.1)[94] with the following settings: (removeFirstG = TRUE, correctSystematicG = FALSE, T = 1mil, alpha = 1.33, TPM threshold = 0.1, TPM singletons to filter <5). Sequences of chrUn and ribosomal DNA[4] were masked from the MorexV3 assembly and only intergenic regions were analyzed for possible eRNAs. The intergenic regions were defined by ChIPseeker (v1.36.0[95]) annotation as > 500 bp apart from an annotated gene.

Based on the observed intergenic expression (RNA-seq data) and lncRNA annotation, the NET-CAGE dataset was further pruned for potential unannotated genes and lncRNAs by removing all regions close to a significant RNA-seq signal (within 500-bp distance of a signal >1 TPM). Based on previous data from mammals about eRNA bi-directionality, we further searched for pairs of NET-CAGE signals transcribed from opposite strands. The clusters that had an antisense NET-CAGE signal of TPM >0.1 (not a singleton) within 600-bp distance were extended, forming bidirectional clusters delineated by the plus strand-dominant TSS and the minus strand-dominant TSS. This merged part of initially separated clusters into bidirectional clusters. The limits for balanced signals were set to log2FoldChange from 1 to −1. Association of the cCREs with NET-CTSSs was tested by a permutation test with 500 cycles using regioneR package.[96] Complete code for the NET-CAGE data analysis is provided in the GitHub repository.

Sequence conservation analysis

Sequences of *H. vulgare* MorexV3[61] were used as the reference for pairwise alignments with the sequences of *T. urartu* v2.0,[109] *S. cereale* Rye_Lo7_2018_v1,[110] *B. distachyon* v3.0,[111] and *Ae. tauschii* v4.0[81] using LAST aligner (v1608)[112] within the nf-core/pairgenomealign workflow (v2.0),[97] followed by processing through the following pipeline: axtChain, chainNet, netToAxt, and axtToMaf (UCSC Genome Browser Toolkit, Anaconda distribution). The resulting pairwise alignments were then combined into a multiple sequence alignment using aligner ROAST (v3).[98] PhastCons score was computed using the PhastCons program from the PHAST package (v1.6).[99] To obtain the neutral and conserved models for PhastCons, the phyloFit program (a part of the PHAST package) was used according to the manual (http://compgen.cshl.edu/phast/phastCons-HOWTO.html). Conservation scores for sets of sequences (cCREs or exons of high-confidence genes as defined in MorexV3) were calculated as averages from all per-base PhastCons scores included in each sequence. Moreover, for visualization in the genome browser, we performed MSA of five Triticeae genomes, including *H. vulgare* MorexV3, *Ae. tauschii* v4.0, *T. aestivum* v1.0,[113] *T. urartu* v2.0, and *S. cereale* Rye_Lo7_2018_v1, using the procedure described above.

ChromHMM chromatin state analysis

ChromHMM (v1.23)[44] was used according to the manual (https://compbio.mit.edu/ChromHMM/) to learn seven chromatin states for each of the developmental stages. Previously published (embryonic stages[5]) and newly generated (leaf) ChIP-seq and ATAC-seq mapped and deduplicated data were binarized from BAM files, while the 'coding potential' and UMRs were binarized from BED files using the '-peaks' option, all with a bin size of 200 bp. The binarized data were used to learn seven chromatin states, followed by Overlap Enrichment analysis.

To provide condensed information on the three analyzed histone modifications (H3K4me3, H3K27me3, and H3K9ac), leaf ChIP-seq data from[6] and this study were used alone to learn four chromatin states, complementing similar datasets previously generated for embryonic tissues.[5]

RSAT motif analysis

To determine core motifs in cCREs, stage-specific sets of robust cCREs were subjected to peak-motif position analysis by Regulatory Sequence Analysis Tools (RSAT) integrated with footprintDB.[100,114] The analysis was followed by motif-clustering to eliminate redundancy. Custom settings are documented in the rsat_analysis.txt deposited in the GitHub repository.

GO term analysis

Using our previously generated GOMAP GO annotation[5,101] and the 'enricher' function from the 'clusterProfiler'[102] package with default settings, we determined the enrichment of GO terms for interacting and non-interacting genes. To reduce the redundancy of the results, we utilized the REVIGO RESTful API.[103] From this pruned set, only the upper quartile of enriched GO terms was used for visualization.

Cell Genomics, Volume *6*

Supplemental information

Epigenome and interactome profiling uncovers principles of distal regulation in the barley genome

Pavla Navratilova, Simon Pavlu, Zihao Zhu, Zuzana Tulpova, Ondrej Kopecky, Petr Novak, Nils Stein, and Hana Simkova

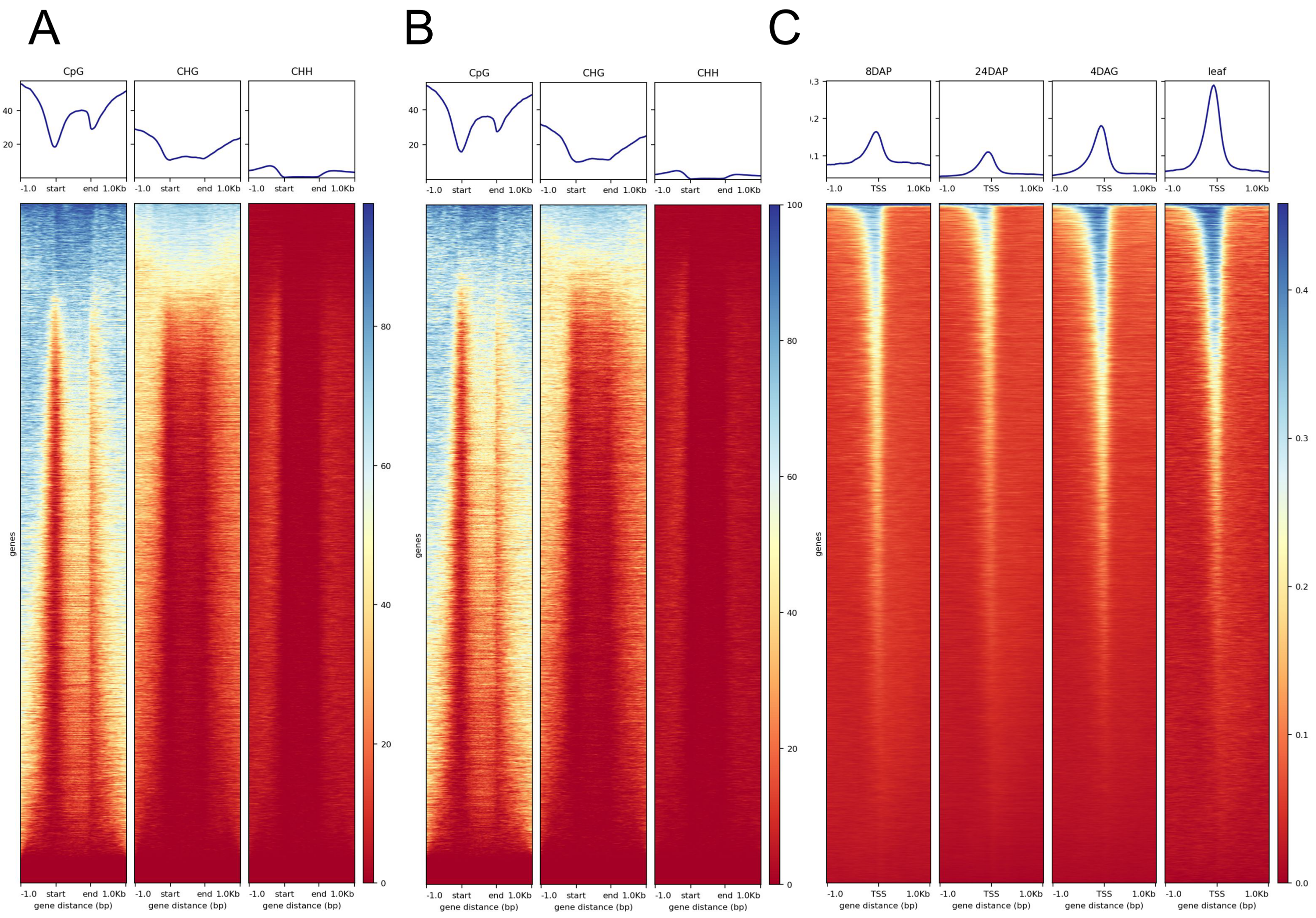

Figure S1. Profiles and heatmaps showing read coverages for two epigenetic features across MorexV3 high-confidence genes (related to Figure 1B). Data from bisulfite sequencing show cytosine methylation in three sequence contexts in (A) 24DAP embryo and (B) leaf. (C) Profiles of ATAC-seq coverages around transcription start sites (TSSs) in the four developmental stages used in the study.

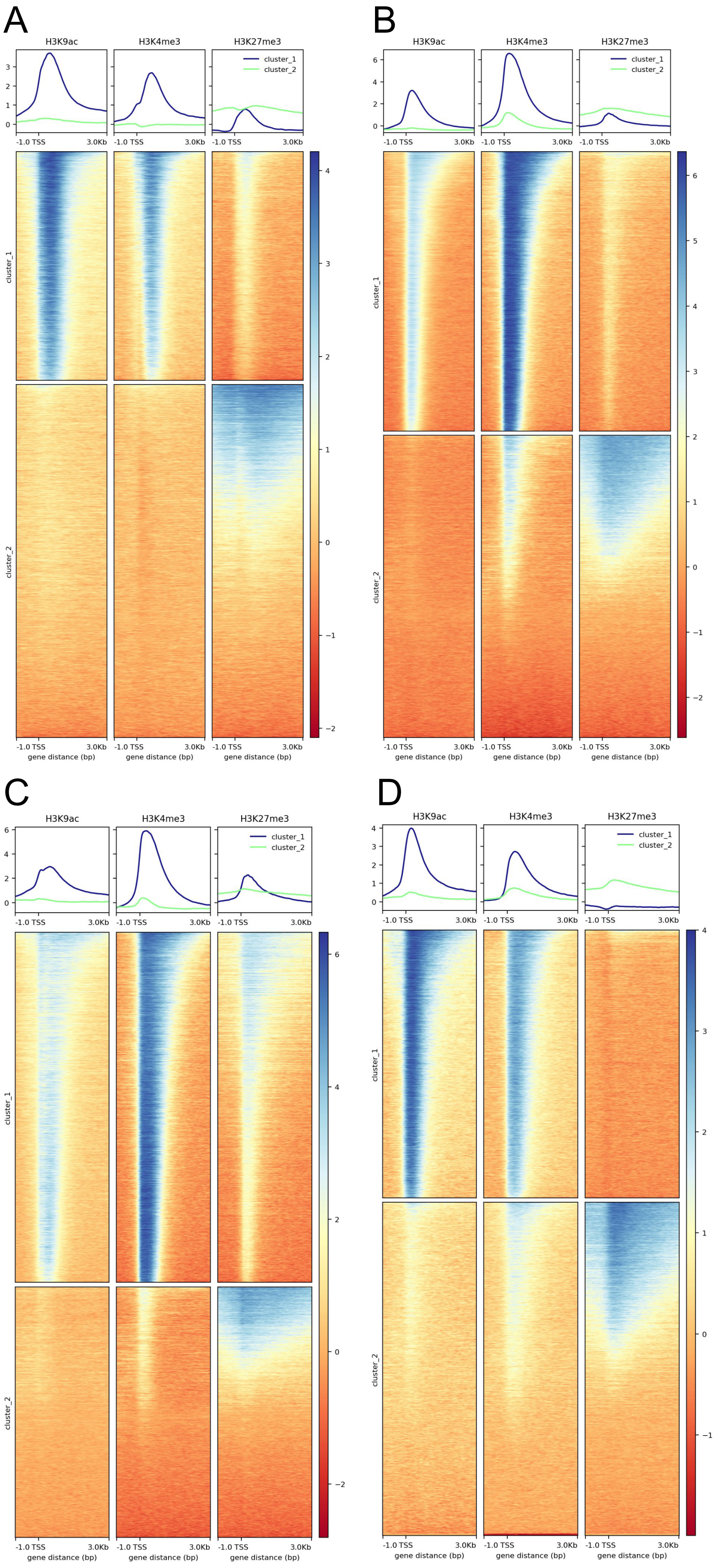

Figure S2. K-means clustered profiles of histone modifications around HC-gene TSSs from ChIP-seq (related to Figure 1B). (A) Profiles in 8DAP embryo. Cluster 1 (top) represents active genes while cluster 2 (bottom) shows profiles of silent genes. (B, C, D) Data from 24DAP (B), 4DAG (C) and leaf (D) samples. The k-means clustering separates two sub-profiles reflecting transcriptional activity.

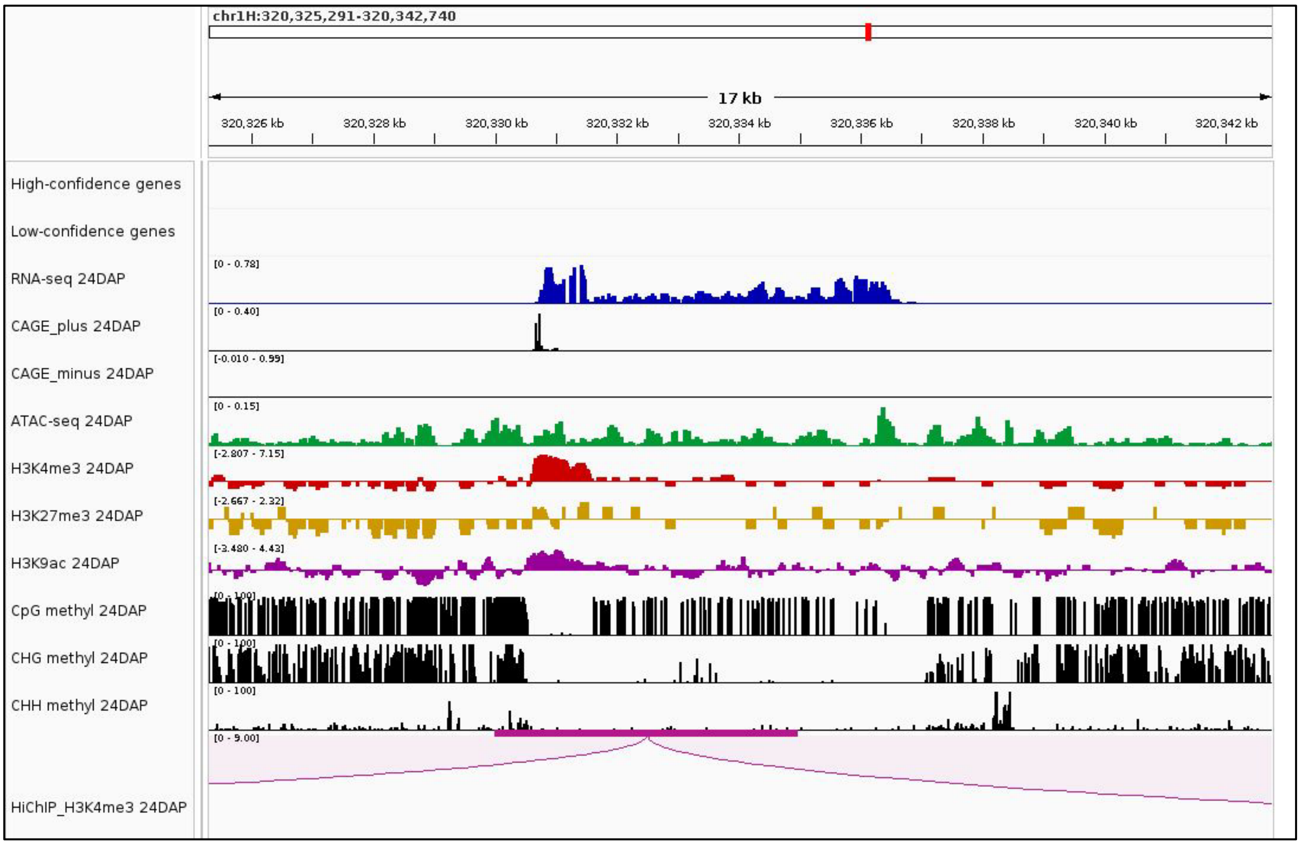

Figure S3. An example of a putative unannotated gene (related to Figure 1C). The occurrence of transcripts and features of active chromatin indicates the presence of an unannotated gene. To prevent misinterpretation of its promoter as an intergenic CRE, we excluded this and similar regions from the analysis of the non-coding genome.

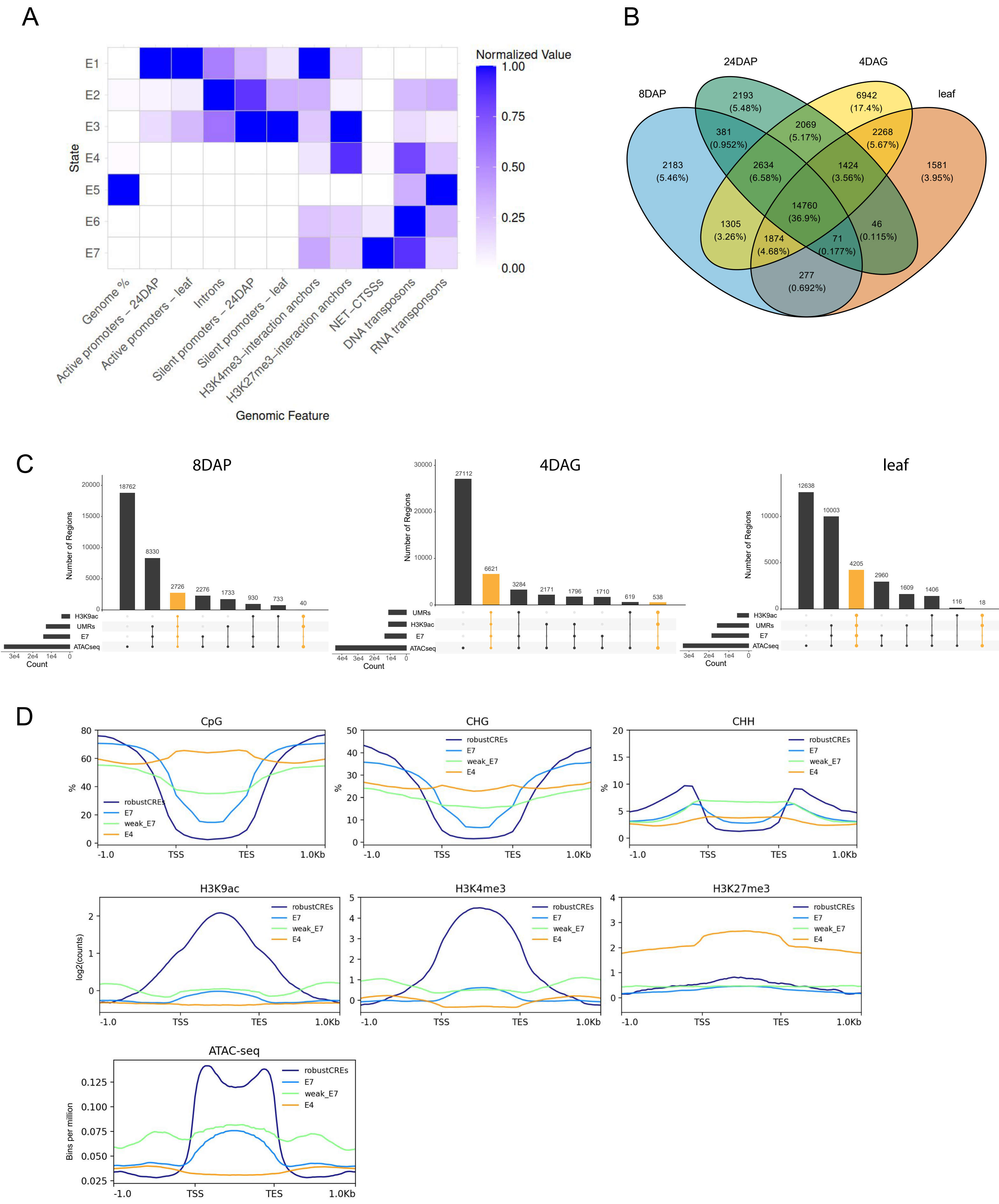

Figure S4. Chromatin state analysis is a sensitive method of cCREs detection (related to Figures 1C, D, and 2A, B). (A) An overlap enrichment of genomic features across 7 chromatin states in the leaf sample showing state dynamics following transcription dynamics between individual stages. (B) Dynamics of chromatin state-E4 segments, potentially comprising silenced cCREs. (C) The overlap of activating intergenic genomic features - peaks of ATAC-seq and H3K9ac and unmethylated regions (UMRs) - defines tissue-specific sets of robust cCREs (highlighted yellow), which shows a high overlap with E7 segments. 'Count' indicates the total number of peaks for ATAC-seq while for E7, H3K9ac and UMRs, overlaps of these datasets with ATAC-seq peaks are counted. (D) Comparison of main chromatin feature profiles across robust cCREs, states E4, E7 and the E7-state segments not overlapped with any of the epigenetic feature peaks (weak E7).

A

8DAP robust CRE motif	e-value	Transcription factor family
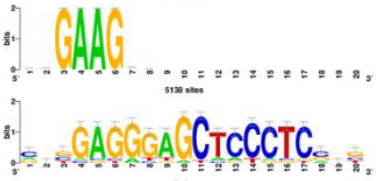	6.5e-20	BBR-BPC<br><br>Palindrome of the GAGA motif
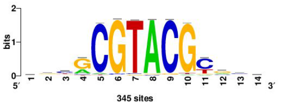	5.1e-07	SPL
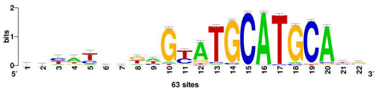	3.6e-06	B3 domain-containing

B

4DAG robust CRE motif	e-value	Transcription factor family
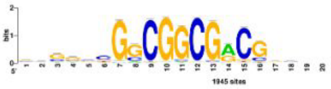	7.6e-101	ERF
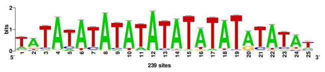	1.9e-63	ZFHD10-3
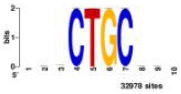	9.8e-07	NAC, ONAC
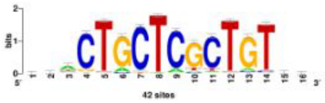	0	No hit

C

leaf robust CRE motif	e-value	Transcription factor family
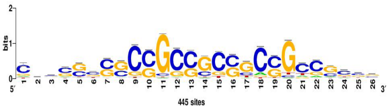	1e-300	ERF
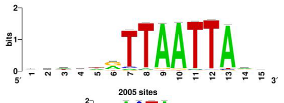	1.5e-32	WOX
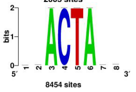	8.9e-04	AP2/ERF and B3 domain-containing

Figure S5. Transcription factor binding sites in robust cCREs (related to Figure 2D). Transcription factor binding sites enriched in (A) 8DAP, (B) 4DAG, and (C) leaf robust cCRE datasets.

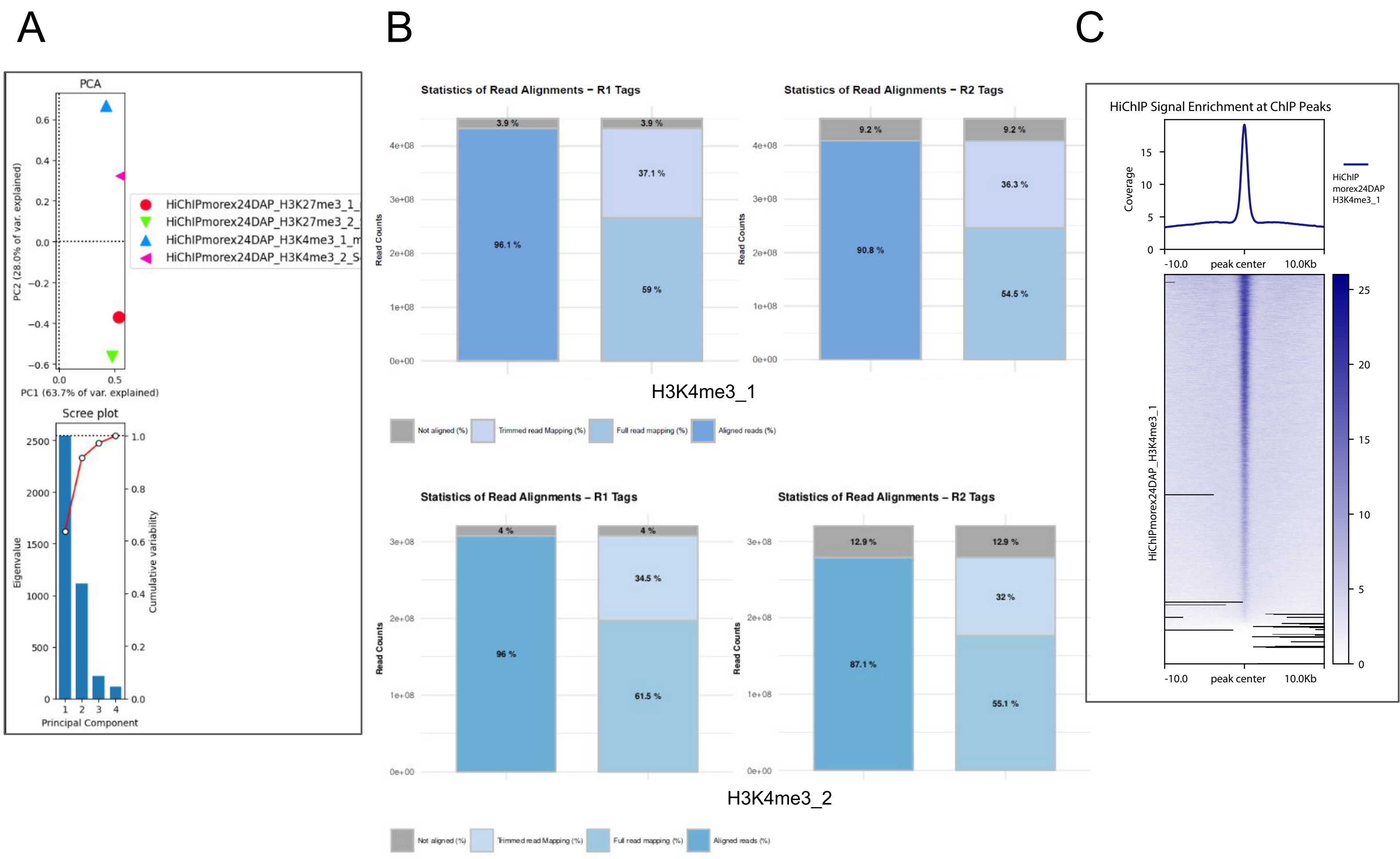

Figure S6. Quality Control of the 24DAP H3K4me3 HiChIP sequencing datasets (related to STAR Methods - HiChIP data analysis). (A) HiChIP replicate correlation of mapped data by Principal Component Analysis (PCA). (B) Mapping statistics of the two 24DAP H3K4me3 HiChIP replicates. (C) HiChIP signal enrichment at the H3K4me3 ChIP peaks as called from previously performed native ChIP-seq experiment.

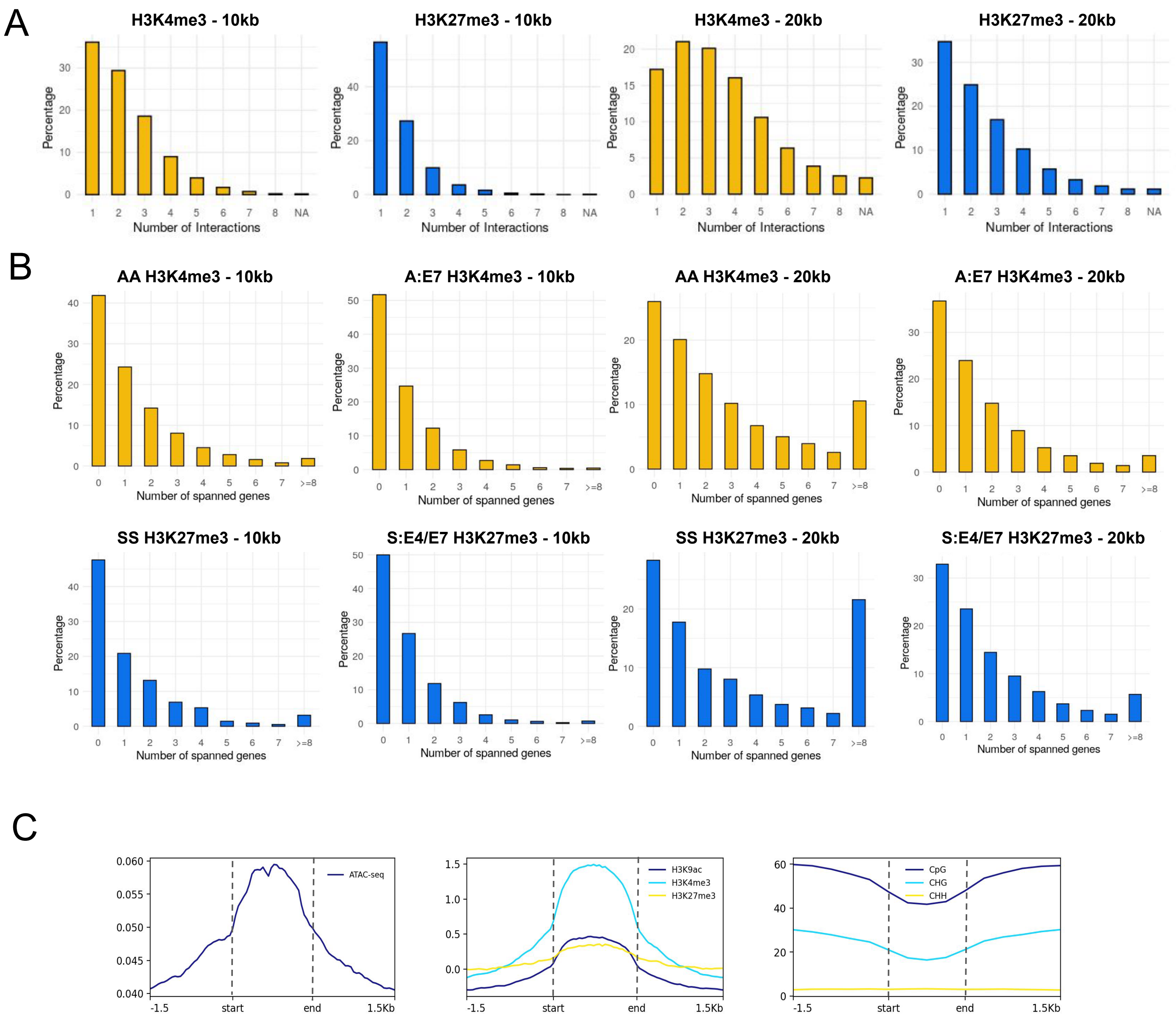

Figure S7. Analysis of the interactome in the 24DAP barley embryo (related to Figure 4). (A) Numbers of H3K4me3 and H3K27me3 HiChIP interactions per promoter at 10- and 20-kb resolution. (B) Numbers of genes spanned by H3K4me3 and H3K27me3 HiChIP interactions at 10- and 20-kb resolution. (C) Enrichments of selected epigenetic features across H3K4me3 HiChIP anchors at 5-kb resolution indicates that they are transcriptionally dense regions.

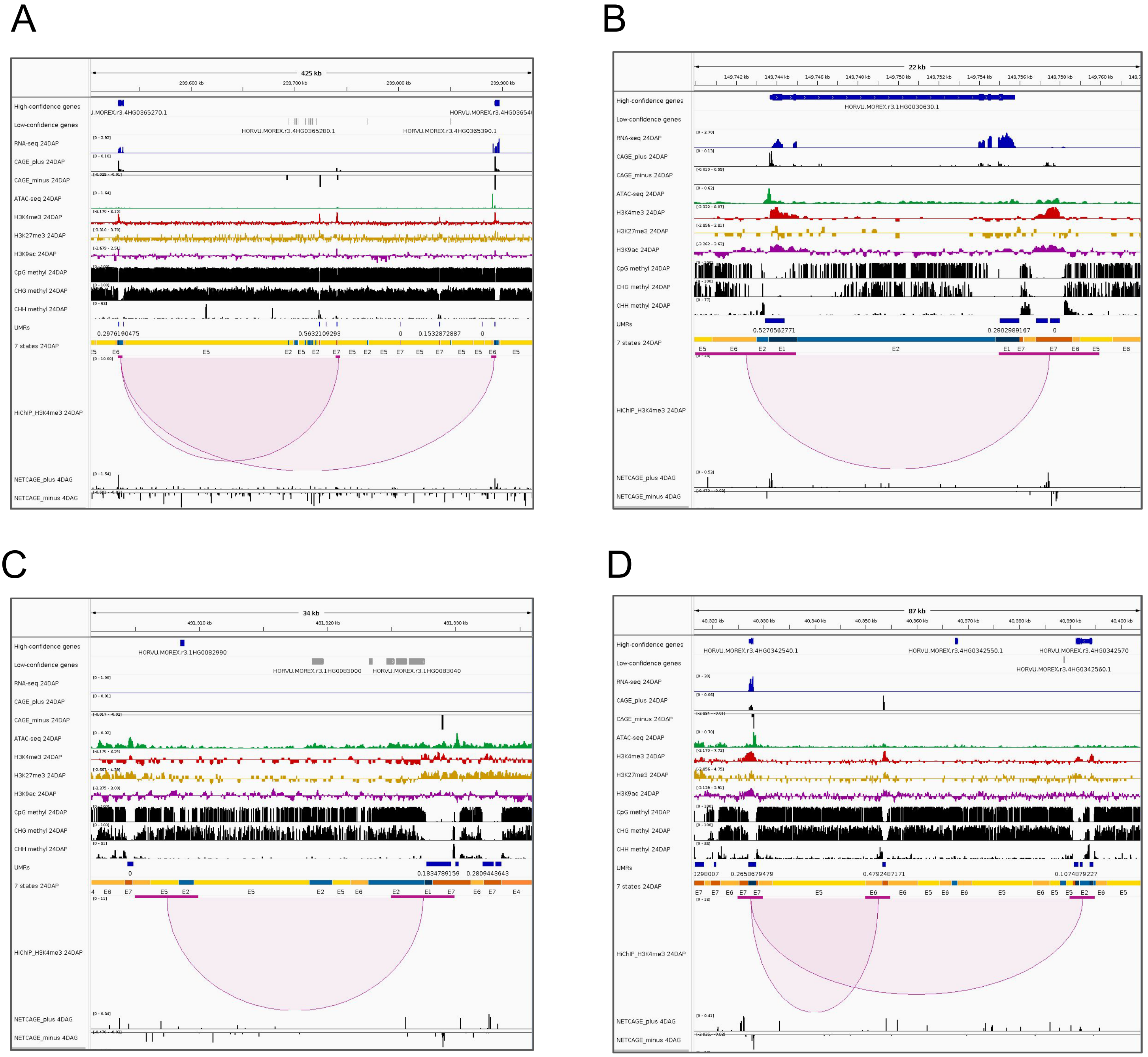

Figure S8. Examples of distinct interaction classes together with epigenomic features (related to Figure 4). (A) Active promoter-active promoter, (B) active self-gene loop, (C) silent promoter-CREc, (D) active promoter-CREc and active promoter-silent gene.

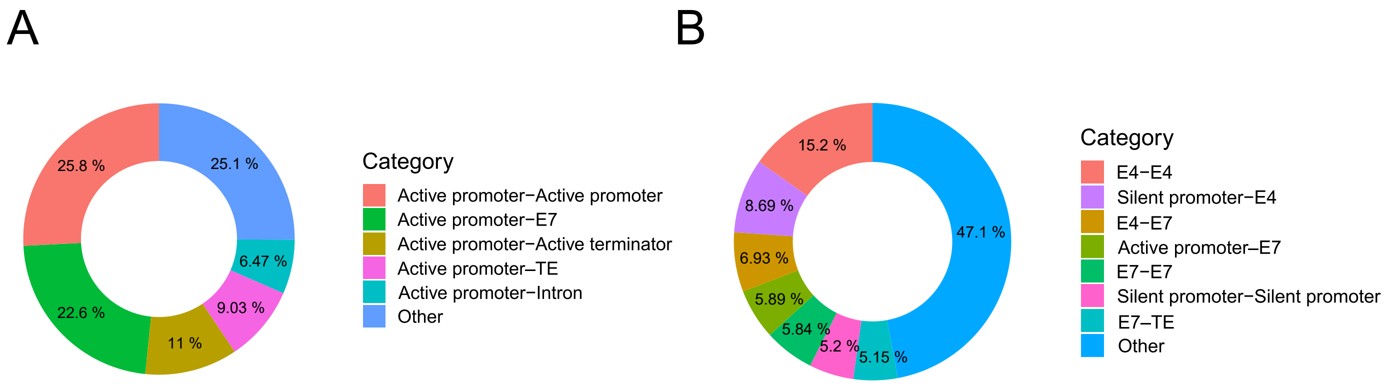

Figure S9. Proportions of interaction classes identified by HiChIP at 5-kb resolution in 24DAP embryo, focusing on high-confidence genes (related to Figure 5). Annotation of all significant interactions associated with (A) activating (H3K4me3) and (B) repressive (H3K27me3) histone mark. The category „Other“ includes all interaction classes with <5% frequency.

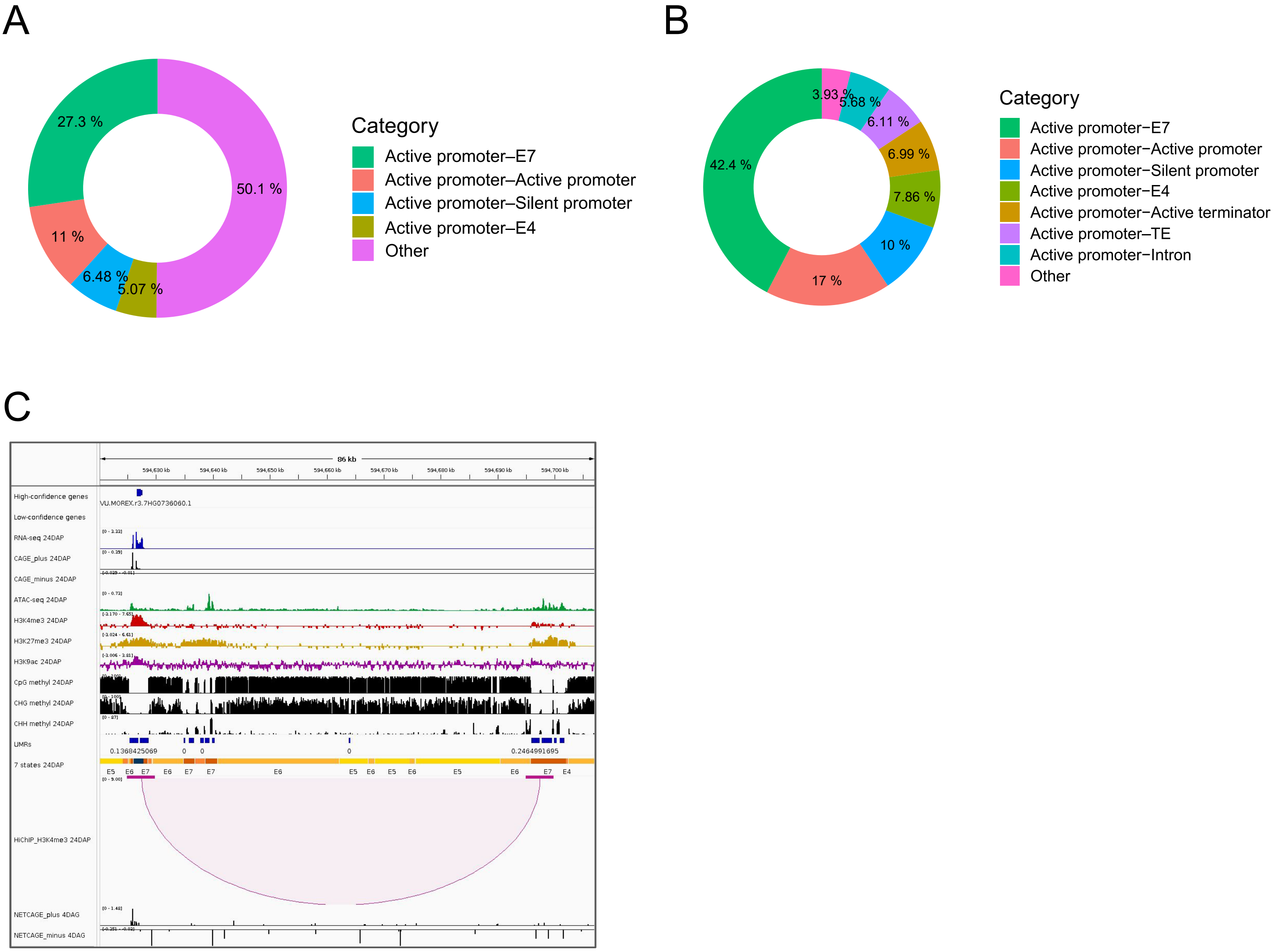

Figure S10. Features of bivalent interactions (related to Figure 5). Annotation of all bivalent interactions (A), and the same set from the active promoter-centric view (B). The category „Other“ includes all interaction classes with <5% frequency. (C) An example of a bivalent interaction.

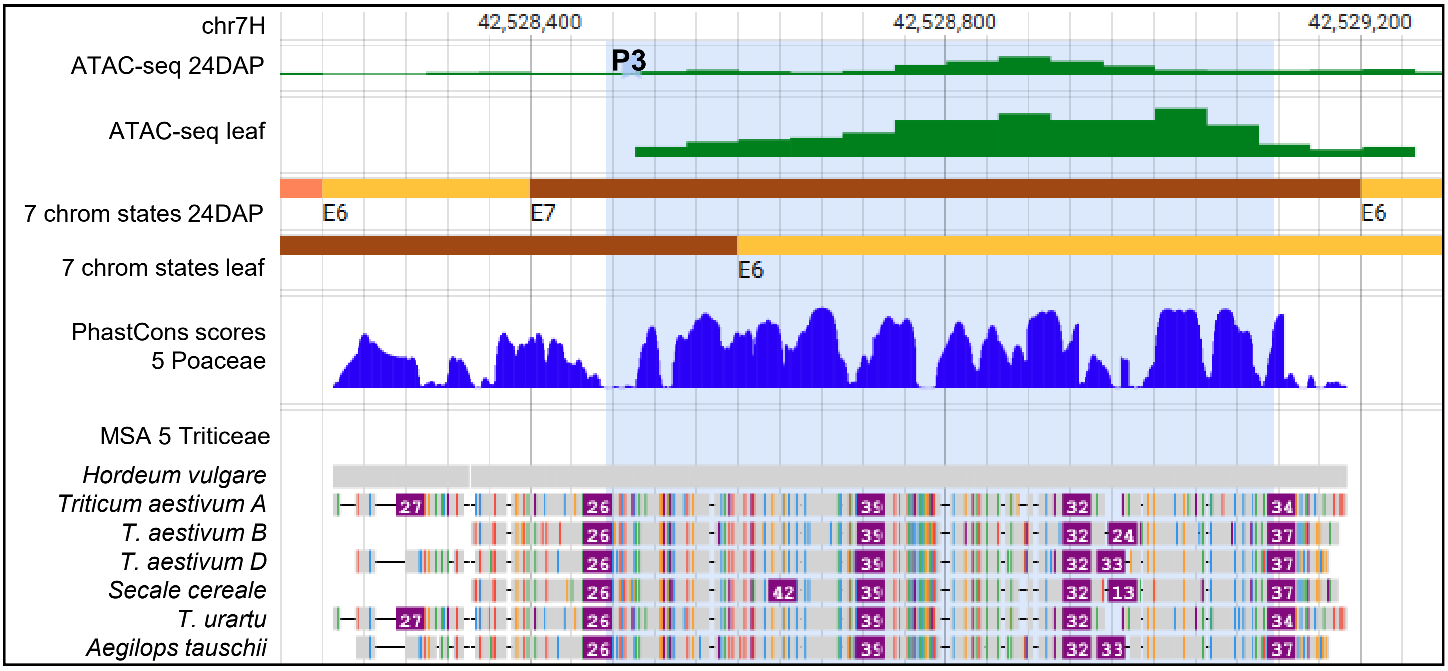

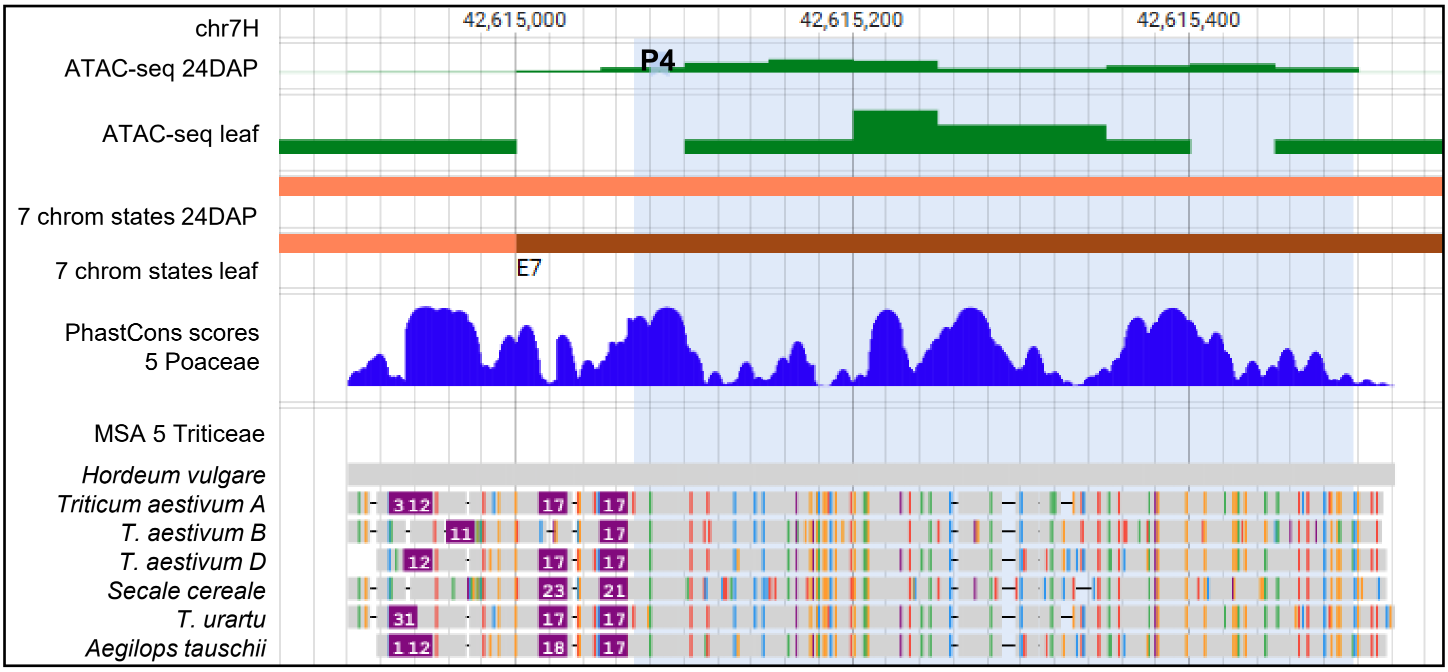

Figure S11. Regulome of the barley Vernalization 3 gene: evolutionary conservation (related to Figure 7). Regions of cCRE3 (top) and cCRE4 (bottom) with BLAST hits of bread wheat enhancers P3 and P4 (highlighted blue) show high sequence conservation (PhastCons scores, scale 0-1) among five *Poaceae* species (*H. vulgare*, *Triticum urartu*, *Aegilops tauschii*, *Secale cereale* and *Brachypodium distachyon*). Multiple sequence alignment (MSA) shows the conservation at single-base resolution.

Table S1. Peak numbers in barley embryo and leaf ChIP-seq and ATAC-seq datasets (related to STAR Methods - ATAC-seq and ChIP-seq data analysis)

ATAC-seq	8DAP
53303	Morex8DAP_ATACseq_1
55758	Morex8DAP_ATACseq_2
42899	Morex8DAP_ATACseq_intersect
66139	Morex8DAP_ATACseq_merged
	**24DAP**
145943	Morex24DAP_ATACseq_1
127166	Morex24DAP_ATACseq_2
93441	Morex24DAP_ATACseq_intersect
	**4DAG**
54098	Morex4DAG_ATACseq_1
29863	Morex4DAG_ATACseq_2
10627	Morex4DAG_ATACseq_intersect
73333	Morex4DAG_ATACseq_merged
	**Leaf**
64153	MorexLeaf_ATACseq_pooled

ChIP-seq	H3K9ac - 8DAP
22130	Morex8DAP_H3K9ac_1
26393	Morex8DAP_H3K9ac_2
15911	Morex8DAP_H3K9ac_intersect
32610	Morex8DAP_H3K9ac_merged
	**24DAP**
32119	Morex24DAG_H3K9ac_1
66104	Morex24DAG_H3K9ac_2
30159	Morex24DAG_H3K9ac_intersect
	**4DAG**
61885	Morex4DAG_H3K9ac_1
30178	Morex4DAG_H3K9ac_2
25994	Morex4DAG_H3K9ac_intersect
46140	Morex4DAG_H3K9ac_merged
	**Leaf**
35765	MorexLeaf_H3K9ac_intersect

ChIP-seq	H3K4me3 - 8DAP
12976	Morex8DAP_H3K4me3_1a
16198	Morex8DAP_H3K4me3_2
10075	Morex8DAP_H3K4me3_intersect
19099	Morex8DAP_H3K4me3_merged
	**24DAP**
47454	Morex24DAP_H3K4me3_1
51563	Morex24DAP_H3K4me3_2
46220	Morex24DAP_H3K4me3_intersect
	**4DAG**
53653	Morex4DAG_H3K4me3_1a
48396	Morex4DAG_H3K4me3_2a
46140	Morex4DAG_H3K4me3_intersect
	**Leaf**
19951	MorexLeaf_H3K4me3_pooled

ChIP-seq	H3K27me3 - 8DAP
46915	Morex8DAP_H3K27me3_1
43595	Morex8DAP_H3K27me3_2
38506	Morex8DAP_H3K27me3_intersect
	**24DAP**
59006	Morex24DAP_H3K27me3_1
51373	Morex24DAP_H3K27me3_2
48316	Morex24DAP_H3K27me3_intersect
	**4DAG**
71668	Morex4DAG_H3K27me3_1a
85421	Morex4DAG_H3K27me3_2a
70564	Morex4DAG_H3K27me3_intersect
	**Leaf**
37098	MorexLeaf_H3K9ac_intersect

Intergenic peaks Filtered using "Coding potential set of 77364 regions including HC and LC genes, lncRNA candidates and RNA-seq positive regions"

ATAC-seq	8DAP
35530	Intergenic_8DAP_ATACseq_intersect
	**24DAP**
55250	Intergenic_24DAP_ATACseq_intersect
	**4DAG**
43851	Intergenic_4DAG_ATACseq_merged
	**Leaf**
32955	Intergenic_Leaf_pooledReps

H3K9ac	8DAP
	8009
	**24DAP**
	14351
	**4DAG**
	27579
	**Leaf**
	8485

H3K4me3	8DAP
	4527
	**24DAP**
	11810
	**4DAG**
	11764
	**Leaf**
	1864

UMRs	
total	139283
intergenic	74614

Table S4. NET-CAGE statistics and quality control (related to STAR Methods - NET-CAGE data analysis)

Sample	Total	rRNA-filtered	BWA-Mapped		BWA-Others		Totally Mapped	Totally Used
				Total	HISAT2_mapped	HISAT2_Unique		
N.4DAG.1	49,565,707	49,565,707<br>(100.00%)	17,044,862<br>(34.39%)	35,287,860<br>(71.19%)	28,886,338<br>(81.86%)	4,349,102<br>(15.06%)	45,931,200<br>(92.67%)	21,393,964<br>(46.58%)
N.4DAG.2	42,976,826	42,976,826<br>(100.00%)	17,103,499<br>(39.80%)	31,220,523<br>(72.65%)	20,964,092<br>(67.15%)	3,143,385<br>(14.99%)	38,067,591<br>(88.58%)	20,246,884<br>(53.19%)

rRNA-filtered: the retained reads after the reads from rRNA were filtered.
BWA-Mapped: BWA-mapped read with MAPQ >= 20.
BWA_Others: the read with MAPQ < 20 in BWA mapping. These reads were mapped again by HISAT2.
HISAT2_Unique: HISAT2-mapped with the tag NH:i:1
Totally Mapped: sum of BWA-Mapped and HISAT2-mapped.
Totally Used: sum of BWA-Mapped and HISAT2_Unique. These reads were used for subsequent analysis.

Table S5. Numbers of interactions in all HiChIP samples as identified by FitHiChIP (related to STAR Methods - HiChIP data analysis)

Embryo stage	Histone modification	Resolution	FDR value	Interactions Counts	Single-feature Counts
24DAP	H3K27me3	20kb	0,05	46525	18588
24DAP	H3K27me3	20kb	0,1	47291	17811
24DAP	H3K27me3	10kb	0,1	19323	NA
24DAP	H3K27me3	5kb	0,05	1193	657
24DAP	H3K27me3	5kb	0,1	3751	2158
24DAP	H3K4me3	20kb	0,05	46213	13893
24DAP	H3K4me3	20kb	0,1	57476	18288
24DAP	H3K4me3	10kb	0,1	28578	NA
24DAP	H3K4me3	5kb	0,05	8038	4014
24DAP	H3K4me3	5kb	0,1	9991	5034

Table S6. Quantification of various types of interactions in the HiChIP significant-interaction set (related to Figure 5 and Figure S9)

(A) 24DAP_H3K4me3_Q0.1 interaction set at 5 kb resolution

**category**	**count**
TE-TE	124
TE-Active_promoter	902
TE-Active_terminator	112
TE-E7	337
TE-E4	22
TE-Intron	62
TE-Silent_promoter	56
TE-Silent_terminator	9
TE-Distal	4
Active_promoter-Active_promoter	2578
Active_promoter-Active_terminator	1104
Active_promoter-E7	2255
Active_promoter-E4	116
Active_promoter-Intron	646
Active_promoter-Silent_promoter	424
Active_promoter-Silent_terminator	72
Active_promoter-Distal	26
Active_terminator-Active_terminator	42
Active_terminator-E7	274
Active_terminator-E4	9
Active_terminator-Intron	47
Active_terminator-Silent_promoter	45
Active_terminator-Silent_terminator	6
Active_terminator-Distal	3
E7-E7	236
E7-E4	47
E7-Intron	135
E7-Silent_promoter	89
E7-Silent_terminator	16
E7-Distal	7
E4-E4	32
E4-Intron	10
E4-Silent_promoter	37
E4-Silent_terminator	4
E4-Distal	0
Intron-Intron	32
Intron-Silent_promoter	25
Intron-Silent_terminator	1
Intron-Distal	1
Silent_promoter-Silent_promoter	36
Silent_promoter-Silent_terminator	7
Silent_promoter-Distal	0
Silent_terminator-Silent_terminator	0
Silent_terminator-Distal	1
Distal-Distal	0

Table S6. Quantification of various types of interactions in the HiChIP significant-interaction set (related to Figure 5 and Figure S9)

(B) 24DAP_H3K27me3_Q0.1 interaction set at 5 kb resolution

**category**	**count**
Intron-Intron	45
Intron-Silent_promoter	59
Intron-Silent_terminator	21
Intron-E4	68
Intron-E7	77
Intron-Active_promoter	41
Intron-TE	30
Intron-Active_terminator	17
Intron-Distal	0
Silent_promoter-Silent_promoter	195
Silent_promoter-Silent_terminator	117
Silent_promoter-E4	326
Silent_promoter-E7	170
Silent_promoter-Active_promoter	108
Silent_promoter-TE	100
Silent_promoter-Active_terminator	33
Silent_promoter-Distal	0
Silent_terminator-Silent_terminator	3
Silent_terminator-E4	69
Silent_terminator-E7	28
Silent_terminator-Active_promoter	12
Silent_terminator-TE	13
Silent_terminator-Active_terminator	5
Silent_terminator-Distal	0
E4-E4	571
E4-E7	260
E4-Active_promoter	115
E4-TE	184
E4-Active_terminator	33
E4-Distal	3
E7-E7	219
E7-Active_promoter	221
E7-TE	193
E7-Active_terminator	78
E7-Distal	4
Active_promoter-Active_promoter	68
Active_promoter-TE	75
Active_promoter-Active_terminator	68
Active_promoter-Distal	1
TE-TE	86
TE-Active_terminator	23
TE-Distal	3
Active_terminator-Active_terminator	8
Active_terminator-Distal	1
Distal-Distal	0
